# Outcome Measures and ID‐COSM Reporting Standards in Alveolar Ridge Augmentation: A Systematic Review of Clinical Trials

**DOI:** 10.1111/jcpe.70176

**Published:** 2026-07-23

**Authors:** Khushboo Kalani, Hamoun Sabri, Maaham Lodhi, Pranav Kataria, Shivam Patel, Lucrezia Parma‐Benfenati, Muhammad H. A. Saleh

**Affiliations:** ^1^ Department of Periodontics and Oral Medicine University of Michigan School of Dentistry Ann Arbor Michigan USA; ^2^ Department of Periodontology University of Connecticut, School of Dental Medicine Farmington Connecticut USA

**Keywords:** alveolar ridge augmentation, clinical trial, dental implantsoutcome assessmentpatient‐reported outcome measurestreatment outcome

## Abstract

**Aim:**

To evaluate outcome measures in clinical studies on alveolar ridge augmentation (ARA) and map them to the Implant Dentistry Core Outcome Set and Measurement (ID‐COSM) framework and the Food and Drug Administration (FDA) terminology.

**Methods:**

A systematic search was carried out across four databases through June 2025. Randomised and non‐randomised clinical trials, prospective clinical studies, cohort studies and pilot clinical studies evaluating ARA performed before or simultaneously with implant placement were included. Extracted outcomes were mapped to ID‐COSM domains, subdomains and specific outcomes, and all were classified into FDA‐style outcome types. Risk of bias (RoB) was assessed using Domain 4 of RoB 2.0/ROBINS‐I, and associations between RoB and completeness of outcome reporting were analysed with Spearman's rank correlation and Kruskal–Wallis tests (α = 0.05).

**Results:**

Four hundred and twenty‐nine studies were included, predominantly randomised controlled trials (56.4%). Less than half of the studies fulfilled the mandatory ID‐COSM domains (47.6%), and none reported all nine subdomains. For mandatory outcomes, surgical complications (52.7%) and implant placement feasibility (48.3%) were more frequently reported than bone dimensional changes (33.8%), and peri‐implant bone stability was reported in only 11.0% of studies. For FDA categorisation, clinician‐reported outcomes (86.3%) and biomarkers (87.7%) predominated, while patient‐reported outcomes were reported in 31.9% of studies. Observer‐reported or performance outcomes were completely absent. Lower risk of bias was associated with more complete reporting of outcomes across domains, subdomains and specific outcomes (*p* = 0.032, 0.017 and 0.009, respectively).

**Conclusion:**

Outcome reporting for ARA trials is highly heterogeneous and poorly aligned with the standardised minimum outcomes recommended by ID‐COSM and FDA categorisation.

## 1 Introduction

Adequate alveolar bone volume is essential for optimal implant placement, yet it is often compromised by resorption, disease, trauma or congenital defects. To address this, alveolar ridge augmentation (ARA) is routinely employed in clinical practice. However, the evidence base evaluating ARA is highly heterogeneous, and studies have used a wide range of clinical, radiographic, histological and patient‐centred endpoints to characterise outcomes (Shi et al. 2023b, 2023a).

There is stark inconsistency in how outcomes are documented across studies, including measures like bone gain, implant success (Misch et al. 2008), microscopic analysis (Mtanis et al. 2023) and patient‐reported outcomes. (Avila‐Ortiz et al. 2023). Outcome selection for ARA often depends on prior studies or clinical judgement, where it remains heterogeneous, and a unified definition of treatment success is still evolving (Saleh, Sabri, et al. 2026). A review of 208 ARA studies found that complications and implant survival were commonly reported, whereas only 15.7% of studies used patient‐reported outcomes. Socioeconomic factors were rarely addressed (Shi et al. 2023b, 2023a).

Inconsistent reporting across studies undermines comparability and clinical relevance, complicating systematic reviews and increasing selective outcome reporting, which affects over 50% of trials in major dental journals (Chan et al. 2004; Sendyk et al. 2019; Wang et al. 2024). To address these issues, the Core Outcome Sets and Measures (COSM) framework has been proposed to standardise trial reporting by specifying the minimum set of outcomes that should be measured and reported in clinical trials within a specific healthcare area (Williamson et al. 2012). The Bone Augmentation Core Outcome Set and Measurements (BA‐COSM) identified mandatory domains, such as surgical complications, peri‐implant tissue health and patient satisfaction and comfort, applicable across bone augmentation trials (Tonetti et al. 2023).

While current dental research examines various outcomes, it remains dominated by clinician‐reported outcomes (ClinROs) and biomarkers, with limited use of patient‐reported outcomes (PROs), observer‐reported outcomes (ObsROs) and performance outcomes (PerfOs) (Saleh, Schwarz, et al. 2026). Biomarkers in the medical field (contrary to our usual terminology in dentistry) are any objective biological or instrument‐derived measures not dependent on patient motivation or rater interpretation (e.g., lab values or any quantitative radiographic imaging metrics).

The primary aim of the present systematic review is to assess the consistency of outcome reporting in clinical studies of ARA performed either prior to or simultaneously with implant placement by mapping the study's outcomes to BA‐COSM and quantifying adherence to these domains. The secondary aim is to determine and quantify the breadth of outcome‐type reporting in ARA studies by classifying each reported endpoint into FDA‐style categories (time‐to‐event/survival, ClinROs, PROs, ObsROs, PerfOs and biomarkers).

## 2 Methods

### 2.1 Protocol Development and Focused Question

This systematic review was conducted in accordance with the Preferred Reporting Items for Systematic Reviews and Meta‐Analyses (PRISMA) 2020 guidelines (Page et al. 2021). The review protocol was prospectively registered and amended in the International Prospective Register of Systematic Reviews (PROSPERO‐CRD420251017374), maintained by the Centre for Reviews and Dissemination at the University of York, United Kingdom.

Focused Question: *In patients requiring dental implant placement, how are outcomes reported and classified in clinical studies evaluating horizontal and/or vertical alveolar ridge augmentation procedures performed either prior to or simultaneously with implant placement, and to what extent do these outcomes align with established core outcome set domains (ID‐COSM and BA‐COSM) and FDA outcome‐type categories?*

#### 2.1.1 Eligibility Criteria

*Inclusion criteria (PICOS)*:

- Population (P): Patients requiring one or more dental implants
- Intervention (I): Bone augmentation procedures (horizontal or vertical ridge augmentation; simultaneous or staged)
- Comparator (C): No need for a comparative group
- Outcome (O): Any outcome that has been used to assess efficacy or performance
- Study design (S): Randomised clinical trials (RCTs), non‐controlled clinical trials (NCCTs), prospective clinical studies, cohort studies, pilot clinical studies.

*Exclusion criteria*:

- Procedures not intended for implant placement
- Immediate implant placement studies
- Sinus floor elevation studies
- Alveolar ridge preservation studies
- Animal/in vitro studies.

### 2.2 Outcome Classification (based on BA‐COSM)

Following the outcome classification system proposed by Tonetti et al., extracted data from the included studies were systematically mapped into a three‐level hierarchical framework (Tonetti et al. 2023). Each level represented a core domain of outcome reporting: (1) outcomes considered mandatory in all trials involving bone augmentation; (2) outcomes mandatory in specific trial types; and (3) outcomes considered important but optional. A domain or subdomain was considered fulfilled if at least one relevant outcome within it was reported (Table 1).

**TABLE 1:** Outcome classification framework based on ID‐COSM and BA‐COSM (Tonetti et al. 2023).

Domain	Subdomain	Identified Outcomes
Mandatory Outcomes (BA‐COSM)	Assessment of surgical complications and adverse events	Graft/membrane exposure; infection; injury to adjacent structures; wound dehiscence/failure; post‐operative pain; swelling; haemorrhage; days of impaired activity; analgesic use
	Bone dimensional changes	Linear measurements (probe or calliper); volumetric measurements (CBCT superimposition, 3D analysis)
	Implant placement feasibility	Ability to place implant in a prosthetically driven position; ridge dimensions at re‐entry; need for augmentation at re‐entry
Mandatory in specific types of trials	Peri‐implant bone stability	Bone thickness; interproximal bone levels
	Mid‐facial mucosal recession	Recession depth; papilla index; pink aesthetic score; buccal tissue thickness; patient‐reported aesthetic satisfaction; soft‐tissue evaluation
	Inclusion of ID‐COSM outcomes	Implant/prosthesis survival; peri‐implant tissue health; surgical complications; overall patient satisfaction and comfort; complication‐free survival
Important but optional	Histology	% new bone; % residual graft; % connective tissue/marrow; bone‐to‐implant contact; bone volume fraction; microradiography; micro‐CT/histomorphometry; immunohistochemistry (e.g., VEGF, Runx2); elemental analysis (e.g., Ca/P ratio)
	Bone defect resolution	% defect fill; linear bone gain; volumetric bone gain; residual defect dimensions; CBCT/CT scans; clinical probing; histologic sectioning
	Quality of life measures	OHIP‐14; SF‐36; EQ‐5D; HADS; functional limitation; physical pain; psychological discomfort; social disability; patient satisfaction scores; return to normal activities

### 2.3 Reported Outcomes

All outcomes reported across the included studies were first compiled into a master list, and a binary ‘yes/no’ was assigned for each outcome to indicate whether it was reported in a given study. Each listed outcome was then categorised according to the standardised FDA definitions described below. Outcome extraction and categorisation were performed independently by one investigator (K.K.), with disagreements resolved by discussion and, where necessary, adjudication by a second investigator (M.H.A.S.).

*A—Survival Outcomes*:

*Overall Survival*: The time from randomisation to death from any cause.

*Event‐Free Survival*: The time from randomisation until complication occurrence, disease recurrence or implant loss from any cause.

*B—Clinical outcome assessments (COA)*:

*Clinician‐Reported Outcomes (ClinROs)*: Reports from a trained healthcare professional regarding their interpretation of signs or behaviours that can be observed and related to a patient's disease or condition are considered clinician‐reported outcomes.

*Patient‐Reported Outcomes (PROs)*: Provide information about the patient's health condition as directly reported by the patient, without interpretation by others.

*Observer‐Reported Outcome (ObsROs)*: Assessments of observable signs, events or behaviours related to a patient's health condition as reported by individuals who observe the patient in daily life, such as parents or caregivers.

*Performance Outcomes (PerfOs)*: Outcomes derived from a patient completing a standardised task that is measured quantitatively by trained personnel, with minimal reliance on rater judgement in scoring.

*C—Biomarkers*: Objective outcome assessments derived from biological or instrument‐based measures that do not depend on patient motivation or subjective rater interpretation (e.g., laboratory values and quantitative imaging metrics).

### 2.4 |Information Sources and Search Strategy

#### 2.4.1 Electronic Search

Four electronic databases were used as sources in the search for studies published from 1988 to 2025 satisfying the inclusion criteria: (1) MEDLINE via PubMed; (2) Cochrane Library (including Cochrane Database for Systematic Reviews and Cochrane CENTRAL register for Clinical Trials); (3) Embase; and (4) Scopus. The search was limited to human subjects and studies reported in English. Reports in other languages were not considered. (Table S1).

#### 2.4.2 Manual Search

All reference lists of the selected studies and previously published systematic reviews were checked for cross‐references.

### 2.5 Screening Methods

Two reviewers (K.K. and M.L.) independently screened titles, abstracts and full texts of studies meeting the inclusion criteria. Disagreements were resolved by discussion with a third reviewer (M.H.A.S.). Inter‐reviewer reliability (kappa coefficient) was calculated (Figure S1).

### 2.6 Data Extraction

Three reviewers (K.K., P.K., S.P.) independently extracted data. Authors were contacted for clarification when information was missing; uncertain data were omitted pending resolution. For multi‐group studies, data were extracted only from groups relevant to the analysis.

### 2.7 Quality Assessment

Quality assessment was done to evaluate how the primary outcome was assessed. Domain 4 from RoB2 was used to describe the risk of bias of the outcome measures for all study types, as this domain is conceptually and operationally equivalent in both RoB 2.0 (for randomised trials) and ROBINS‐I (for non‐randomised studies) (Shi et al. 2023b, 2023a). Other domains of these tools were not applied, as the present review focused specifically on outcome measurement and reporting rather than on overall internal validity.

### 2.8 Data Analyses

Data were summarised descriptively. Outcomes reported in each included study were presented in tables by outcome domain and augmentation procedure type. All outcomes were combined to calculate the overall percentage distribution of each domain across the included studies. Additionally, when outcomes were explicitly identified within a paper, their frequency was calculated separately and stratified by intervention type.

### 2.9 Statistical Analysis

Associations between risk of bias (RoB; categorised as low, some concerns or high) and completeness of outcome reporting (domains, subdomains and specific outcomes fulfilled per ID‐COSM) were assessed using Spearman's rank correlation (*ρ*; RoB treated ordinally: low = 1, some concerns = 2, high = 3) and Kruskal–Wallis tests, with Bonferroni‐adjusted pairwise post hoc comparisons where significant. Associations between ID‐COSM inclusion and RoB were further evaluated using univariable ordinal logistic regression for total outcomes, subdomains and main domains, with odds ratios (ORs) and 95% confidence intervals (CIs) reported (OR < 1 indicating lower odds of higher RoB with greater ID‐COSM inclusion). Binary logistic regression comparing low versus non‐low RoB was performed as a sensitivity analysis. All tests were two‐tailed, with *p* < 0.05 considered statistically significant.

## 3 Results

### 3.1 Characteristics of the Included Studies

A total of 429 articles were included (Supporting Information File S1): RCTs (*n* = 242, 56.4%), prospective clinical studies (*n* = 78, 18.2%), NCCTs (*n* = 39, 9.1%), prospective case series (*n* = 24, 5.6%), prospective cohort studies (*n* = 21, 4.9%), pilot studies (*n* = 11, 2.6%), observational studies (*n* = 5, 1.2%), cross‐sectional studies (*n* = 4, 0.9%) and others (*n* = 5, 1.2%).

Figure S2a shows the annual distribution of these articles from 1988 to 2025. Early years showed minimal output (e.g., *N* = 1 in 1988), while a marked rise began in the 2010s, peaking in 2024 with *N* = 49 articles. Geographic distribution was led by Italy (*N* = 87), United States (*N* = 41), Switzerland (*N* = 38), Egypt (*N* = 34) and China (*N* = 26), with 13 multi‐centre studies (Figure S2b).

### 3.2 Classification According to the ID‐COSM Framework

When outcomes were mapped according to the three‐domain ID‐COSM framework, 47.6% of studies fulfilled all three domains, 40.8% fulfilled two domains, 10.5% fulfilled one domain and 1.2% of the studies reported no domain at all (Figure 1).

**FIGURE 1:**
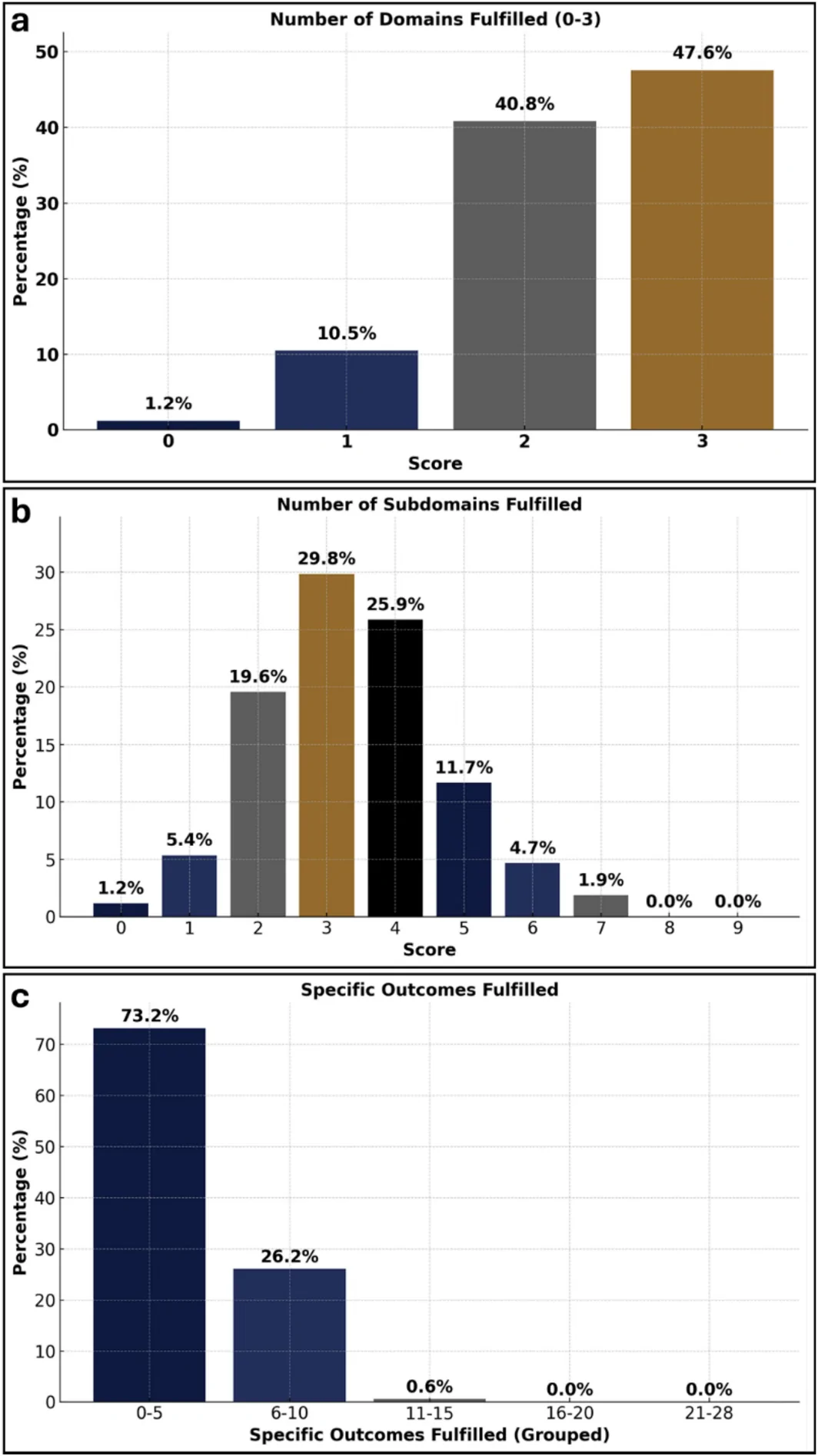
Evidence‐based classification of reported outcomes according to the ID‐COSM framework. (a) Number of domains fulfilled (0–3). Nearly half of the studies fulfilled all three domains. (b) Number of subdomains fulfilled (0–9). Most studies covered 2–4 subdomains, with very few achieving higher coverage. (c) Number of specific outcomes fulfilled (grouped ranges). The majority of studies reported 0–5 specific outcomes, with no study exceeding 15.

For subdomain‐level coverage (maximum nine subdomains), 29.8% of studies fulfilled three subdomains, 25.9% fulfilled four, 19.6% fulfilled two and 11.7% fulfilled five. A smaller proportion met only one subdomain (5.4%) or none of the subdomains (1.2%). Fulfilment of six, seven, eight or more subdomains was rare, with percentages of 4.7%, 1.9% and 0% respectively (Figure 1b).

When assessing the number of specific outcomes reported (maximum 28), nearly three‐quarters of studies (73.2%) reported between 0 and 5 outcomes, while 26.2% reported 6–10 outcomes. Only 0.6% of studies reported 11–15 outcomes, and none reported more than 15 outcomes (Figure 1c).

#### 3.2.1 Subdomain‐Level Assessment According to the ID‐COSM Framework

Outcome reporting varied across ID‐COSM domains (Figure 2). Among mandatory outcomes (Figure 2b), surgical complications were reported in 52.7% of studies, implant placement feasibility in 48.3% and bone dimensional changes in 33.8%. Outcomes mandatory for specific trial types (Figure 2b) showed uneven reporting, with inclusion of ID‐COSM outcomes reported in 80.4% of studies, mid‐facial mucosal recession in 44.6% and peri‐implant bone stability in 11.0%. Important but optional outcomes (Figure 2c) were dominated by bone defect resolution, reported in 82.3% of studies, while histology and quality‐of‐life measures were reported in 17.3% and 12.4%, respectively. The overall distribution of reporting depth is shown in Figure 2d.

**FIGURE 2:**
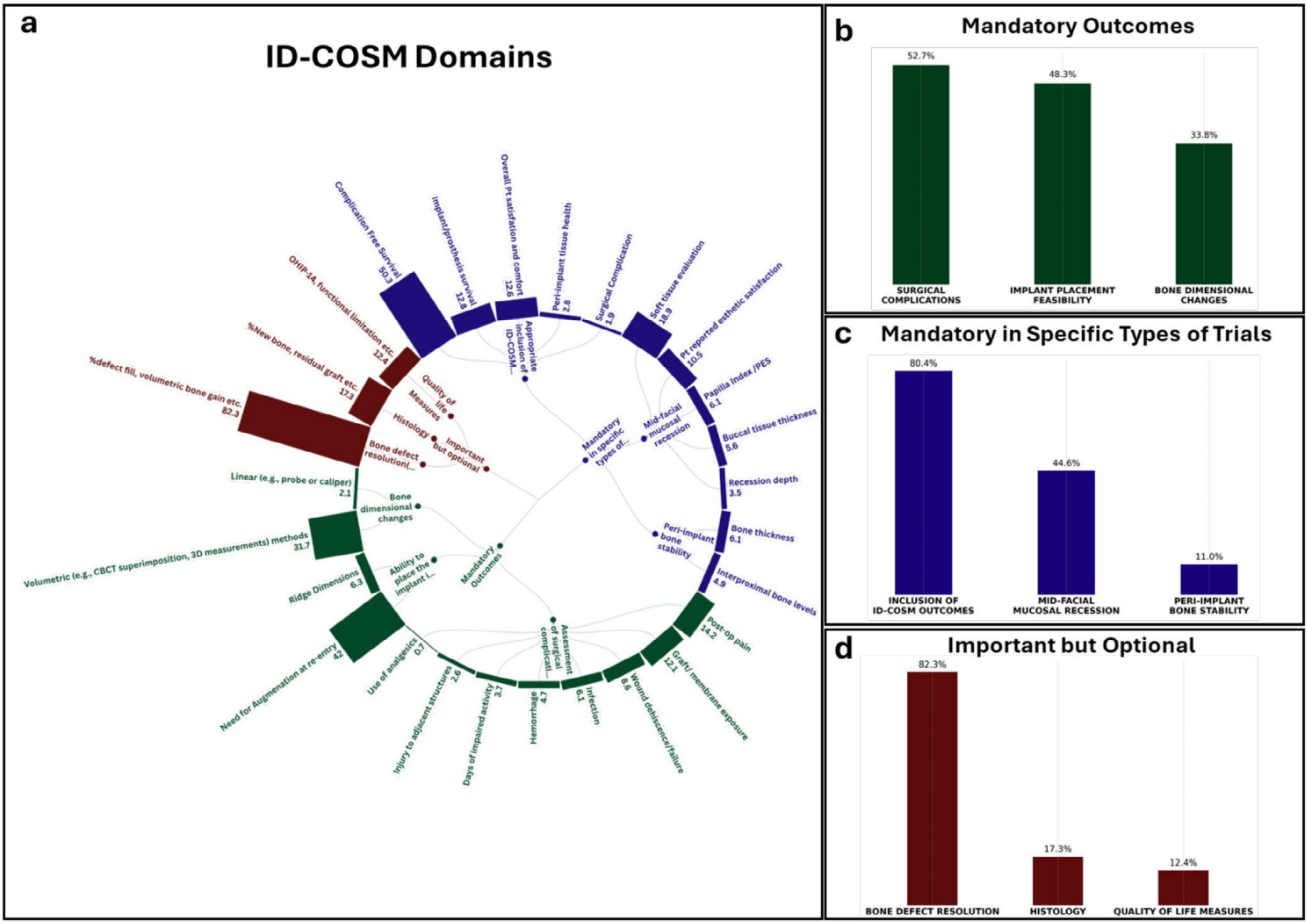
Subdomain‐level assessment of reported outcomes according to the ID‐COSM framework. The circular diagram maps reported outcomes to their respective domains and subdomains, colour‐coded as mandatory outcomes (green), mandatory in specific trial types (blue) and important but optional outcomes (red). The accompanying bar charts display the percentage of included studies reporting each subdomain.

#### 3.2.2 Specific Outcomes Within Each ID‐COSM Domain

##### 3.2.2.1 Mandatory Outcomes

Within the surgical complications and adverse events subdomain, the most frequently reported specific outcomes were postoperative pain (14.2%), graft/membrane exposure (12.1%) and wound dehiscence/failure (8.6%). Less frequently reported were infection (6.1%), haemorrhage (4.7%), days of impaired activity (3.7%), injury to adjacent structures (2.6%) and use of analgesics (0.7%) (Figure S3a–c).

For implant placement feasibility, the need for augmentation at re‐entry was documented in 42.0% of studies, while ridge dimension assessments were reported in only 6.3%.

In bone dimensional changes, volumetric methods—including CBCT superimposition and 3D measurements—were reported in 31.7% of studies, whereas simple linear measurements (e.g., probe or calliper) were reported in only 2.1%.

##### 3.2.2.2 Mandatory in Specific Trial Types

Within the ID‐COSM outcomes subdomain, complication‐free survival was the most common specific measure (50.3%), followed by implant/prosthesis survival (12.8%), overall patient satisfaction and comfort (12.6%), peri‐implant tissue health (2.8%) and surgical complication reporting (1.9%) (Figure S3d–f).

For mid‐facial mucosal recession, the most reported measure was soft‐tissue evaluation (18.9%), followed by patient‐reported aesthetic satisfaction (10.5%), papilla index/PES (6.1%), buccal tissue thickness (5.6%), and recession depth (3.5%).

In peri‐implant bone stability, bone thickness was documented in 6.1% of studies and interproximal bone levels in 4.9%.

##### 3.2.2.3 Important but Optional Outcomes

Here, bone defect resolution was the most frequently reported outcome, observed in 82.3% of studies. Histology was included in 17.3%, while quality‐of‐life measures were reported in only 12.4%, highlighting a significant under‐representation of patient‐centred endpoints in this category (Figure S3g).

#### 3.2.3 Association Between Risk of Bias and Completeness of Outcome Reporting

Higher risk of bias was significantly associated with lower outcome reporting completeness across all three levels of the ID‐COSM hierarchy (Tables 1, 2, Figure 3): domains (*ρ* = −0.104, *p* = 0.032), subdomains (*ρ* = −0.115, *p* = 0.017), and specific outcomes (*ρ* = −0.125, *p* = 0.009), with Kruskal–Wallis tests significant at the subdomain (*p* = 0.014) and specific‐outcome (*p* = 0.029) levels. Ordinal logistic regression confirmed that greater ID‐COSM inclusion was associated with lower odds of higher RoB for total outcomes (OR 0.85, 95% CI 0.75–0.96, *p* = 0.008), subdomains (OR 0.80, 0.68–0.94, *p* = 0.006) and main domains (OR 0.63, 0.48–0.84, *p* = 0.001). Sensitivity analyses using binary logistic regression yielded consistent results (Table 2).

**FIGURE 3:**
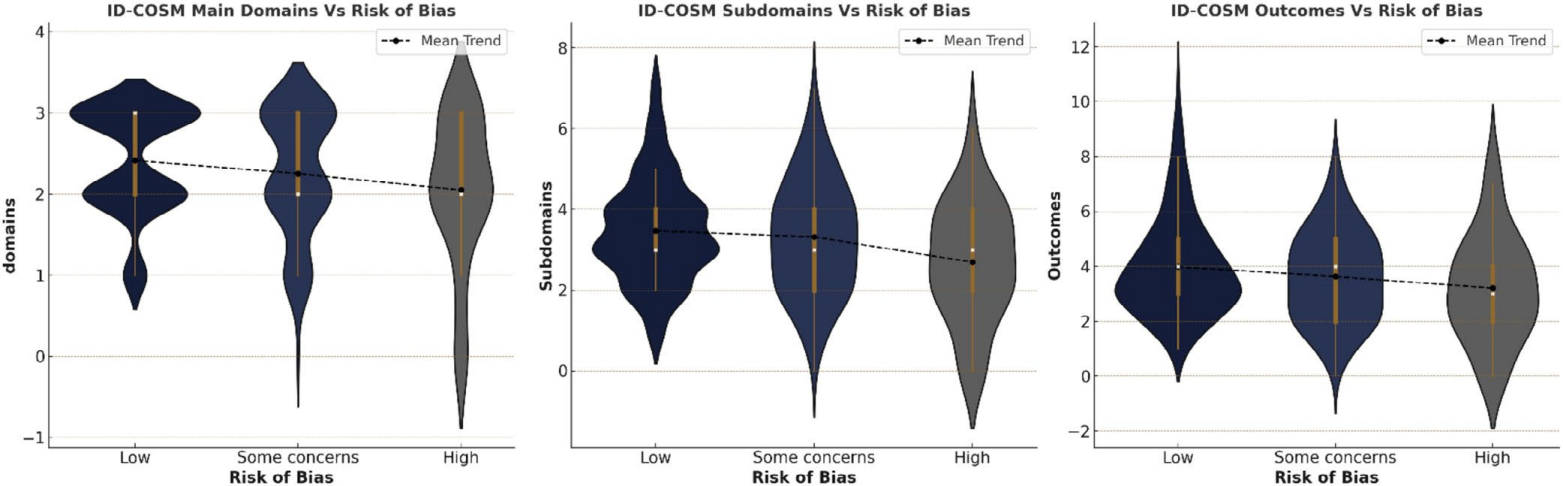
Violin plots displaying the distribution of reporting completeness across three ID‐COSM outcome metrics—main domains, subdomains and specific outcomes—stratified by risk of bias (RoB) levels (low, some concerns, high). Each plot includes an embedded boxplot (median and interquartile range shown in brown) and a dashed black line representing the mean trend across RoB levels. A downward trend indicates that higher RoB is associated with lower completeness of outcome reporting.

**TABLE 2:** Association between risk of bias and completeness of outcome reporting.

	Total Outcomes		Subdomains		Main Domains	
	Mean (SD)	Median	Mean (SD)	Median	Mean (SD)	Median
Part A. Descriptive statistics by risk of bias group						
Low	3.98 (1.85)	4	3.47 (1.29)	3	2.41 (0.65)	3
Some concerns	3.63 (1.67)	4	3.32 (1.43)	3	2.25 (0.77)	2
High	3.21 (1.96)	3	2.69 (1.47)	3	2.05 (0.92)	2

	Spearman's *ρ*/*p*‐value	Spearman's *ρ*/*p*‐value	Spearman's *ρ*/*p*‐value
Part B. Correlation and group difference analyses			
Spearman's *ρ*	−0.104, *p* = **0.032**	−0.115, *p* = **0.017**	−0.125, *p* = **0.009**
Kruskal–Wallis χ^2^	5.47, *p* = 0.064	8.52, *p* = **0.014**	7.12, *p* = **0.029**

Predictor	OR (95% CI)	*p*‐value
Part C. Ordinal logistic regression—OR for higher risk of bias		
Main domains	0.63 (0.48–0.84)	**0.001**
Subdomains	0.80 (0.68–0.94)	**0.006**
Total outcomes	0.85 (0.75–0.96)	**0.008**
Part D. Sensitivity analysis—binary logistic regression, OR for non‐low risk of bias^a^		
Main domains	0.65 (0.49–0.87)	**0.003**
Subdomains	0.83 (0.70–0.97)	**0.017**
Total outcomes	0.86 (0.76–0.97)	**0.014**

*Note:* Significant *p*‐values (*p* < 0.05) shown in bold, OR < 1 indicates greater ID‐COSM inclusion is associated with lower odds of higher risk of bias.

^a^ Sensitivity analysis compares low versus non‐low (some concerns + high) risk of bias.

### 3.3 Classification According to the FDA Terminology

#### 3.3.1 Clinical Outcome Assessments

COA measures were identified in most studies (78.5%) (Figure 4). These were further categorised based on the FDA taxonomy into four types: ClinROs, ObsROs, PROs and PerfOs.

**FIGURE 4:**
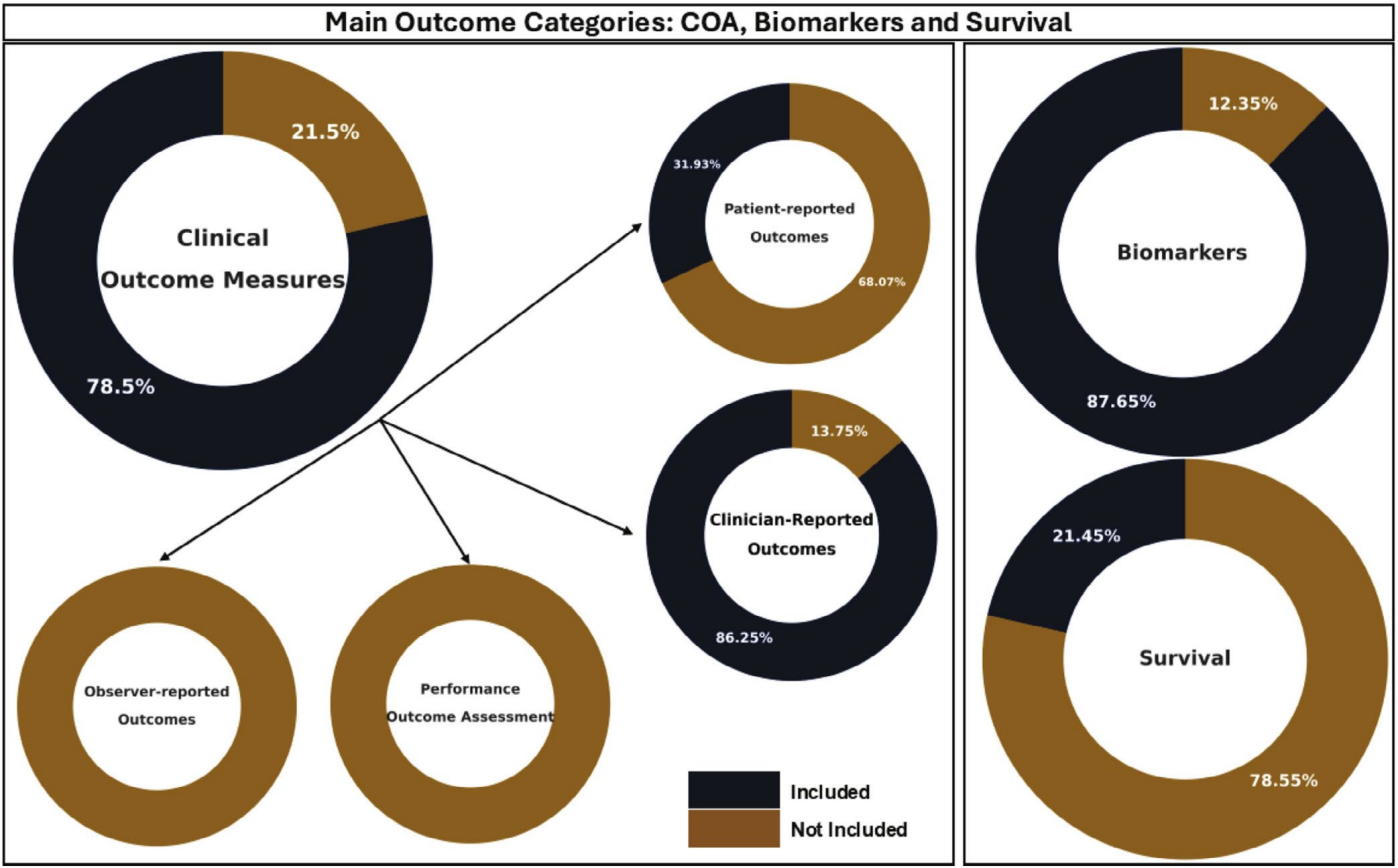
Distribution of the main outcome categories reported in the included studies: Clinical Outcome Assessment (COA) measures; Biomarkers; and Implant Survival. COAs are further subdivided into Clinician‐Reported Outcomes, Patient‐Reported Outcomes, Observer‐Reported Outcomes and Performance Outcome Assessments. Percentages indicate the proportion of studies that included each outcome type.

ObsROs and PerfOs were not represented in any of the reviewed literature.

##### 3.3.1.1 ClinROs

ClinROs were reported in 86.25% of the included studies (Figure 5a). The most frequently assessed ClinROs were clinical attachment level (CAL), reported in 36.6% of studies, followed by clinical bone gain (30.1%), bleeding index/full‐mouth bleeding index (BI/FMBI) (20.5%), crestal or marginal bone level changes assessed via PA or panoramic radiographs (20.1%), gingival index/full‐mouth gingival index (GI/FMGI) (19.8%), and clinical bone fill or healing (17.9%).

**FIGURE 5:**
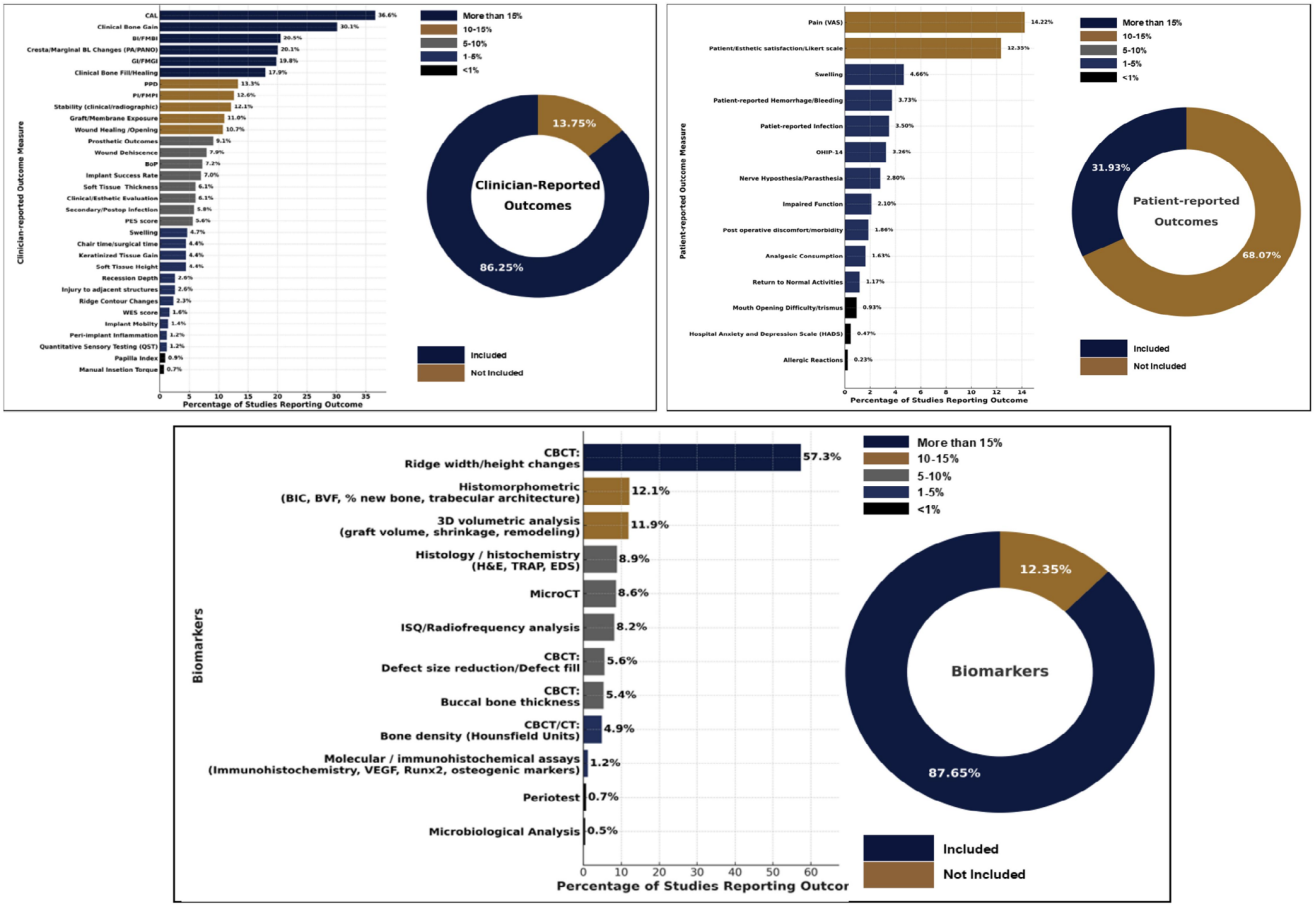
(a) Clinician‐reported outcome measures (ClinROs) in included bone augmentation studies. The donut chart illustrates the proportion of included studies that reported at least one ClinRO (86.25%) versus those that did not (13.75%). The horizontal bar chart shows the specific ClinROs and the percentage of all included studies that reported each measure. (b) Patient‐reported outcome measures (PROMs) in included bone augmentation studies. The donut chart shows the proportion of included studies that reported at least one PROM (31.93%) versus those that did not (68.07%). The horizontal bar chart presents the specific PROs and the percentage of all included studies reporting each measure. Pain was the most commonly reported PROM (14.2%) followed by patient/aesthetic satisfaction (12.35%). (c) Biomarker‐related outcome measures in included bone augmentation studies. The donut chart indicates the proportion of included studies reporting at least one biomarker‐related measure (87.65%) versus those that did not (12.35%). The bar chart details the specific biomarkers and the percentage of all included studies reporting each.

Multiple important outcomes were assessed in fewer studies, including prosthetic outcomes (9.1%), wound dehiscence (7.9%), implant success rate (7.0%), soft‐tissue thickness and clinical/aesthetic evaluation (6.1% each), secondary or postoperative infection (5.8%) and PES score (5.6%). Rarely assessed outcomes (< 2%) included quantitative sensory testing (QST) and papilla index.

##### 3.3.1.2 PROs

PRO measures (PROMs) were reported in 31.9% of the included studies (Figure 5b). The most frequently assessed PROM was based on the Visual Analogue Scale (VAS), reported in 14.2% of studies, followed by patient‐reported aesthetic satisfaction or Likert‐based satisfaction measures (12.3%). Other commonly assessed PROMs included patient‐reported haemorrhage/bleeding (3.7%), patient‐reported infection (3.5%) and OHIP‐14 total score (3.2%).

Less commonly reported PROMs included nerve hypoesthesia/paresthesia (2.8%), impaired function (2.1%), postoperative discomfort or morbidity (1.8%), analgesic consumption (1.6%) and return to normal activities (1.1%).

Rarely reported PROMs (< 1%) were mouth opening difficulty/trismus (0.9%), HADS (Hospital Anxiety and Depression Scale) (0.4%), and allergic reactions (0.2%).

#### 3.3.2 Biomarkers

Biomarker‐related outcome measures were reported in 87.65% of the included studies (Figure 5c). The most frequently evaluated biomarker was ridge width/height changes measured via CBCT, reported in 57.3% of those studies. Other frequently reported biomarkers included histomorphometric analysis (e.g., bone‐to‐implant contact, % new bone, trabecular architecture) at 12.1%, 3D volumetric analysis (e.g., graft volume, shrinkage, remodelling) at 11.9% and histology/histochemistry (e.g., H&E, TRAP, EDS staining) at 8.9%. MicroCT was used in 8.6% of studies. ISQ was reported in 8.2% of studies. Additional CBCT‐based measures included defect fill/reduction (5.6%) and bone density using Hounsfield units (4.9%).

Less commonly reported biomarkers (< 2%) included molecular/immunohistochemical assays (e.g., VEGF, Runx2 and osteogenic markers).

#### 3.3.3 Survival

Overall implant survival was reported in 21.5% of the included studies, typically assessed through follow‐up data on implant retention without failure (Figure 4, bottom right).

### 3.4 Outcomes Reporting Trend Before and After ID‐COSM Conception

RCTs published in 2023 or later showed increased ID‐COSM reporting across all three measures compared to studies published before 2023. The average total outcomes rose from 3.74 ± 1.79 pre‐2023 to 4.23 ± 1.99 in 2023 or later, although this difference was not statistically significant (*p* = 0.085). Similarly, the mean number of reported subdomains increased from 3.30 ± 1.35 to 3.65 ± 1.35 (*p* = 0.072). The number of main domains reported increased significantly following ID‐COSM adoption, from 2.30 ± 0.73 pre‐2023 to 2.53 ± 0.61 in 2023 or later, with the median rising from two to three domains (*p* = 0.011). Although reporting improved post‐2023 across ID‐COSM measures, statistical significance was achieved only for the main domains (Table S2).

## 4 Discussion

The present review found that outcome reporting had significant heterogeneity under the ID‐COSM framework. Mandatory outcomes were inconsistently addressed, and patient‐centred outcomes such as quality of life were infrequently included. In line with the FDA taxonomy for COAs, most outcomes were ClinROs, with less frequent inclusion of PROs and a complete absence of ObsROs and PerfOs. Biomarker‐based measures, especially imaging assessments, were widely used, whereas molecular and microbiological markers were seldom evaluated. Studies with lower bias risk showed significantly more complete reporting within both the ID‐COSM and FDA categories.

Recent methodological and consensus‐driven contributions in implant and periodontal research converge on the view that the field has outpaced its ability to evaluate itself meaningfully (Avila‐Ortiz et al. 2023; Shi et al. 2023b, 2023a; Tonetti et al. 2025; Tonetti et al. 2023). These do not question whether augmentation and implant procedures function technically; instead, they challenge how success is defined, measured and interpreted across clinical studies. The shared premise is that although implant dentistry generates a large volume of outcomes, these outcomes are inconsistently selected, variably defined and often poorly aligned with what constitutes meaningful benefit or harm. As a result, evidence accumulates quantitatively, yet inconsistencies in selected outcomes limit the synthesis and meaningful clinical translation of evidence (Avila‐Ortiz et al. 2023; Kirkham et al. 2016; Tonetti et al. 2023; Williamson et al. 2017). The current study addresses a complementary and implementation‐focused question: to what extent have these principles been adopted in clinical studies of ARA? Mapping our findings to the ID‐COSM framework showed a gradient in outcome reporting that aligns with, but does not fully implement, the ID‐COSM ‘onion’ hierarchy (Tonetti et al. 2023). Outcomes deemed ‘mandatory in all trials’ were reported most consistently, while ‘mandatory in specific trial types’ were assessed less often, with peri‐implant bone and mid‐facial mucosal stability, function and quality of life frequently omitted. This pattern indicates that outcome selection in ARA research predominantly aligns with core, universally applicable safety and feasibility outcomes, whereas condition‐specific domains that operationalise the pathophysiological and functional benefits of augmentation are under‐represented (Avila‐Ortiz et al. 2025). The gradient is most evident in specific outcomes: procedural endpoints such as surgical complications (52.7%) and implant placement feasibility (48.3%), which confirm the surgery was performed and tolerated, are reported more frequently than measures of the procedure's biological success, such as peri‐implant bone stability (11.0%) and quality of life (12.4%). This analysis confirms the procedure's safety but provides limited evidence of its long‐term structural and functional benefits, prompting clinicians and patients to rely more heavily on feasibility data when making treatment decisions.

Importantly, Outcome completeness was not randomly distributed across studies but associated with methodological rigour: studies at lower risk of bias consistently reported more domains, subdomains and specific outcomes, mirroring findings from core outcome set (COS) implementation research that link stronger methodological foundations to higher consensus‐framework adherence (Kirkham et al. 2017; Williamson et al. 2012). Outcome reporting patterns in ARA therefore reflect underlying study design and reporting culture, not reporting alone.

Beyond domain coverage, the FDA‐aligned classification of outcome types reveals a structural imbalance in the evaluation of ARA outcomes. Although clinician‐reported outcomes are essential for documenting biological response and technical success, they fail to capture broader patient‐incurred costs, including treatment‐related discomfort, functional limitations, aesthetic concerns, time burden and economic impact. The absence of PerfOs reflects the structural staging of implant therapy. The augmented ridge serves as an anatomical foundation for subsequent prosthetic rehabilitation rather than a functional endpoint, highlighting a missed opportunity to demonstrate that augmentation yields a functionally superior, not just radiographically better, outcome at the prosthetic stage. ObsROs, in turn, offer limited marginal informational value in adult dental research, where patients are the most direct observers of their own outcomes; for example, patient‐reported aesthetic satisfaction correlates more strongly with clinical outcomes than external indices such as the Pink Aesthetic Score.

Contemporary regulatory and consensus frameworks emphasise that evaluating implant‐related interventions should reflect patient experience and function over time, with clinician‐based and imaging measures serving as complementary, not standalone, indicators of benefit (Arunyanak et al. 2024; Powers 3rd et al. 2017; Tonetti et al. 2025). The core outcome set methodology was developed to address such imbalances by reducing outcome heterogeneity and promoting inclusion of outcomes that capture both benefit and burden (Chiarotto et al. 2017). Without systematic use of validated patient‐reported outcomes and long‐term stability measures, relying on clinician‐reported or surrogate endpoints can overstate success and mask trade‐offs vital for decision making (Shi et al. 2023b, 2023a; Tonetti et al. 2023).

The dual application of ID‐COSM and the FDA framework in this review reflects a deliberate epistemic choice. ID‐COSM and the recent Global Consensus on Clinical Guidelines (GCCG) define core outcomes based on prevailing reporting practices (Fiorellini et al. 2026; Sabri et al. 2026; Schwarz et al. 2026; Tonetti et al. 2025; Tonetti et al. 2023). As a structural consequence, dimensions that have not historically been measured—including patient‐burden measures such as treatment cost, total treatment time and number of clinical visits—remain absent from both consensus frameworks despite their direct relevance to patient decision making. The FDA outcome classification, by contrast, which was developed prescriptively in oncology after biomarker endpoints, most notably tumour response, proved inadequate proxies for benefit when patients experienced significant functional and quality‐of‐life decline despite ‘successful’ technical outcomes (Powers 3rd et al. 2017).

Periodontology offers an analogous cautionary precedent in the gingivectomy era, in which technically valid reductions in probing depth were achieved at the cost of aesthetic and functional consequences that ultimately led patients to refuse further treatment (Knowles et al. 1979). Applying both frameworks in parallel allows the present review to characterise ARA reporting against consensus expectation and against a patient‐benefit‐anchored architecture simultaneously, rather than awaiting a further consensus exercise to bridge the two. A recent 20‐year longitudinal analysis also demonstrated that surrogate periodontal endpoints showed only modest discrimination for the true endpoints: despite achieving initial ‘treatment success’, 91.5% of patients required re‐treatment (Saleh, Alrmali, et al. 2026). The same applies for ARA, with a recent review reporting that patients treated with bone augmentation were significantly less satisfied overall, and those with longer periods of missing tooth (due to staged augmentation timelines) reported lower satisfaction (Duong et al. 2022).

Several limitations should be acknowledged. Outcome mapping relied on published reports, making it difficult to distinguish between outcomes that were not assessed and those selectively unreported. Classification within COS frameworks required interpretive judgement when outcome definitions or measurement strategies were vague or overlapping, underscoring the need for clearer standardisation rather than undermining the frameworks themselves. The inclusion of studies spanning nearly four decades introduced temporal heterogeneity related to evolving surgical techniques, imaging modalities and expectations regarding complication and patient‐reported outcome reporting. A further limitation is that the present review focused on the outcomes themselves rather than the specific measures used to quantify them. Future ARA trials should prospectively align with COS‐based frameworks, define endpoints a priori, standardise measurement instruments and time points and integrate validated patient‐reported outcomes alongside long‐term biological stability measures as core components of treatment evaluation.

## 5 Conclusion

Outcome reporting in ARA research is highly inconsistent with the ID‐COSM and FDA frameworks, favouring procedural feasibility and clinician‐ or imaging‐based measures over long‐term biological stability and patient‐experienced outcomes. Systematic adoption of ID‐COSM and integration of patient‐centred and longitudinal outcomes are needed to improve the comparability, interpretability and clinical relevance of future ARA research.

## Author Contributions

M.H.A.S. and K.K.: conceptualisation and supervision. K.K., P.K., M.L. and S.P.: Data curation, investigation and screening. H.S.: Formal analysis and statistical analysis. K.K., M.L. and H.S.: Writing – original draft preparation. M.H.A.S.: Writing – review, editing and supervision.

## Funding

The authors have nothing to report.

## Conflicts of Interest

The authors declare no conflicts of interest.

## Supporting information


**Data S1:** jcpe70176‐sup‐0001‐Supinfo1.docx.

**Figure S1:** PRISMA flow chart showing the article selection process.
**Figure S2:** (a) Chronological distribution of the studies included. (b) Regional distribution of the studies.
**Figure S3:** Specific outcomes reported within each ID‐COSM domain. Mandatory outcomes (green) include detailed reporting on surgical complications, implant placement feasibility and bone dimensional changes. Mandatory in specific trial types (blue) encompass survival‐related metrics, soft‐tissue assessments and peri‐implant bone stability measures. Important but optional outcomes (red) highlight the predominance of defect resolution reporting, with fewer studies incorporating histology or quality of life measures.
**Table S1:** Search strategy.
**Table S2:** ID‐COSM reporting before versus 2023 or later (*p*‐values are from Mann–Whitney *U*‐tests comparing studies published before 2023 vs. 2023 or later).
**File S1:** Full bibliographic list of the 429 included studies analysed in this systematic review (provided as a separate pdf).

**Data S2:** jcpe70176‐sup‐0003‐Supinfo3.docx.

## Data Availability

The data that support the findings of this study are available from the corresponding author upon reasonable request.
