# Supplementary material for: Outcome Measures and ID‐COSM Reporting Standards in Alveolar Ridge Augmentation: A Systematic Review of Clinical Trials
Source: J Clin Periodontol. 2026 Jul 23;53(11):1638–49. doi: 10.1111/jcpe.70176 (PMC13658050; doi:10.1111/jcpe.70176)
Supplement: Supplementary file 1 [file JCPE-53-1638-s001.docx]

**Outcome Measures and ID-COSM Reporting Standards in Alveolar Ridge Augmentation: A Systematic Review of Clinical Trials.**

**Khushboo Kalani**^1^**, Hamoun Sabri**^1^**, Maaham Lodhi**^1^**, Pranav Kataria**^2^**, Shivam Patel**^1^**, Lucrezia Parma-Benfenati**^1^**, Muhammad H. A. Saleh**^1^

**Supplementary Material**

**Supplementary Figure 1. Prisma flow chart showing the article selection process**

Studies from databases/registers **(n = 13063)**

Scopus (n = 5073)

CENTRAL (n = 2996)

Embase (n = 368)

PUBMED (n = 4626)

References from other sources **(n = 0)**

Citation searching (n =0)

Grey literature (n =0)

**Identification**

Studies excluded **(n = 6148)**

Studies not retrieved **(n =176)**

Studies assessed for eligibility **(n = 1250)**

Studies sought for retrieval **(n = 1426)**

Studies screened **(n = 7597)**

Studies excluded **(n = 821)**

Wrong setting (n = 340)

Wrong intervention (n =481)

Wrong patient population (n = 0)

References removed **(n = 5464)**

Duplicates identified manually (n = 1)

Duplicates identified by Covidence (n = 5463)

Marked as ineligible by automation tools (n = 0)

Other reasons (n = )

**Screening**

Included studies ongoing **(n = 0)**

Studies awaiting classification **(n = 0)**

**Included**

Studies included in review **(n = 429)**

**Supplementary Figure 2. (a)** Chronological distribution of the studies included. **(b)** regional distribution of the studies.

**Supplementary Figure 3.** Specific outcomes reported within each ID-COSM domain. Mandatory outcomes (green) include detailed reporting on surgical complications, implant placement feasibility, and bone dimensional changes. Mandatory in specific trial types (blue) encompass survival-related metrics, soft tissue assessments, and peri-implant bone stability measures. Important but optional outcomes (red) highlight the predominance of defect resolution reporting, with fewer studies incorporating histology or quality of life measures.

**Supplementary Table 1**: Search strategy

| **Search strategies in PubMed:** | |
| --- | --- |
| #1 | dental implants[MeSH Terms] OR dental implants, single tooth[MeSH Terms] OR (dental implantation, endosseous[MeSH Terms] OR (dental implantation, osseointegrated[MeSH Terms] OR (dental implants[Title/Abstract] |
| #2 | augment*[Title/Abstract] OR graft*[Title/Abstract] OR regenerate*[Title/Abstract] OR onlay[Title/Abstract] OR (bone regenerations[MeSH Terms] OR bone replacement materials[MeSH Terms] |
| #3 | #1 AND #2 |
| #4 | [MeSH Terms] OR augmentations, alveolar ridge[MeSH Terms] OR maxillary sinus floor augmentation[MeSH Terms] OR augmentations, sinus floor[MeSH Terms] OR socket preservation[Title/Abstract] OR ridge augment*[Title/Abstract] OR sinus floor elevation[Title/Abstract] OR sinus floor augmentation[Title/Abstract] OR sinus lift[Title/Abstract] OR oral surgical procedures, preprosthetic[MeSH Terms] OR surgical procedure, oral, preprosthetic[MeSH Terms] OR preprosthetic oral surgical procedures[MeSH Terms] |
| #5 | #3 OR #4 |
| #6 | Cohort[Title/Abstract] OR Prospective[Title/Abstract] OR Retrospective[Title/Abstract] OR case series[Title/Abstract] OR randomised[Title] OR randomized[Title] OR trial*[Title] OR controlled[Title] OR Randomized Controlled Trial[Publication Type] |
| #7 | #5 AND #6 |
| #8 | #7 AND ("2011/01/01"[Date - Publication] : "2021/04/01"[Date - Publication]) |

| **Search strategies in Embase** | |
| --- | --- |
| #1 | augment*:ab,ti OR graft*:ab,ti OR regenerat*:ab,ti |
| #2 | 'tooth implant'/exp OR (dental AND implants) |
| #3 | #1 AND #2 |
| #4 | ((augmented:ab,ti OR augmentation:ab,ti) AND ('alveolar ridge':ab,ti)) |
| #5 | 'sinus floor augmentation':ab,ti OR 'sinus lift':ab,ti OR 'sinus floor elevation':ab,ti OR 'socket preservation':ab,ti |
| #6 | #3 OR #4 OR #5 |
| #7 | cohort:ab,ti OR prospective:ab,ti OR retrospective:ab,ti OR 'case series':ab,ti OR random*:ab,ti OR control*:ab,ti OR trial:ab,ti |
| #8 | #6 AND #7 AND (2011:py OR 2012:py OR 2013:py OR 2014:py OR 2015:py OR 2016:py OR 2017:py OR 2018:py OR 2019:py OR 2020:py OR 2021:py) |

| **Search strategies in Cochrane CENTRAL** | |
| --- | --- |
| #1 | [mh "dental implants"] OR ("dental implantation"):ti,ab,kw OR ("dental implant"):ti,ab,kw OR ("dental implantology"):ti,ab,kw OR ("dental implantologies"):ti,ab,kw (Word variations have been searched) |
| #2 | [mh ("guided bone regeneration"):ti,ab,kw OR ("bone regeneration"):ti,ab,kw OR (ridge augmentation):ti,ab,kw |
| #3 | #1 AND #2 AND “Publication date 01/01/2011-04/01/2021” |

| **Search strategies in SCOPUS** | |
| --- | --- |
| #1 | (TITLE-ABS-KEY("dental implantation" OR "dental implant" OR "dental implantology" OR "dental implantologies"))  AND (TITLE-ABS-KEY("guided bone regeneration" OR "bone regeneration" OR "ridge augmentation"))  AND (TITLE-ABS-KEY(evaluation OR outcomes)) |

**Supplementary Table 2.** ID-COSM Reporting Before vs 2023 or Later (P-values from Mann–Whitney U tests comparing studies published before 2023 vs 2023 or later.)

| ID-COSM measure | Before 2023, mean ± SD | 2023 or later, mean ± SD | Median before | Median 2023+ | p-value |
| --- | --- | --- | --- | --- | --- |
| Total outcomes | 3.74 ± 1.79 | 4.23 ± 1.99 | 3 | 4 | 0.085 |
| Subdomains | 3.30 ± 1.35 | 3.65 ± 1.35 | 3 | 3 | 0.072 |
| Main domains | 2.30 ± 0.73 | 2.53 ± 0.61 | 2 | 3 | **0.011** |

**Supplementary File 1:** Full bibliographic list of the 429 included studies analyzed in this systematic review, provided as a separate PDF.
