# Supplementary material for: Outcome Measures and ID‐COSM Reporting Standards in Alveolar Ridge Augmentation: A Systematic Review of Clinical Trials
Source: J Clin Periodontol. 2026 Jul 23;53(11):1638–49. doi: 10.1111/jcpe.70176 (PMC13658050; doi:10.1111/jcpe.70176)
Supplement: Supplementary file 3 [file JCPE-53-1638-s003.docx]

1. Aboelela, S. A. A., Atef, M., Shawky, M., & Fa5ouh, H. (2022). Ridge augmentaBon using autologous concentrated growth factors enriched bone graF matrix versus guided bone regeneraBon using naBve collagen membrane in horizontally deﬁcient maxilla: A randomized clinical trial. Clin Implant Dent Relat Res, 24(5), 569-

579. h5ps://doi.org/10.1111/cid.13121

2. Ahmet, S., Alper Gultekin, B., Karabuda, Z. C., & Olgac, V. (2016). Two Composite Bone GraF SubsBtutes for Maxillary Sinus Floor AugmentaBon: Histological, Histomorphometric, and Radiographic Analyses. Implant Dent, 25(3), 313-321. h5ps://doi.org/10.1097/id.0000000000000378
3. Al-Dubai, S. A. S., Abdel-Rahman, F. H., Ahmed, W., & Tawﬁk, M. A. (2022). Comparison between Modiﬁed Bone-spliang Technique and DistracBon Osteogenesis in Horizontal Alveolar Ridge Expansion: Randomized Clinical Study. J Contemp Dent Pract, 23(10), 1008-1015. h5ps://doi.org/10.5005/jp-journals-10024-3423
4. Aludden, H., Starch-Jensen, T., Dahlin, C., Sdik, J., Cederlund, A., & Mordenfeld, A. (2024). Histological and radiological outcome aFer horizontal guided bone regeneraBon with bovine bone mineral alone or in combinaBon with bone in edentulous atrophic maxilla: A randomized controlled trial. Clin Oral Implants Res, 35(4), 396-406. h5ps://doi.org/10.1111/clr.14235
5. Amorﬁni, L., MiglioraB, M., Signori, A., Silvestrini-BiavaB, A., & BenedicenB, S. (2014). Block allograF technique versus standard guided bone regeneraBon: a randomized clinical trial. Clin Implant Dent Relat Res, 16(5), 655-667. h5ps://doi.org/10.1111/cid.12040
6. Angelo, T., Marcel, W., Andreas, K., & Izabela, S. (2015). Biomechanical Stability of Dental Implants in Augmented Maxillary Sites: Results of a Randomized Clinical Study with Four Diﬀerent Biomaterials and PRF and a Biological View on Guided Bone RegeneraBon. Biomed Res Int, 2015, 850340. h5ps://doi.org/10.1155/2015/850340
7. Anis, M., Abdelrahman, A. R., Aaa, R., & Zahran, A. (2024). Tomographic assessment of bone changes in atrophic maxilla treated by split-crest technique and dental implants with platelet-rich ﬁbrin and NanoBone(®) versus platelet-rich ﬁbrin alone: Randomized controlled trial. BMC Oral Health, 24(1), 691. h5ps://doi.org/10.1186/s12903-024-04420-5
8. Antoun, H., Sitbon, J. M., MarBnez, H., & Missika, P. (2001). A prospecBve randomized study comparing two techniques of bone augmentaBon: onlay graF alone or associated with a membrane. Clin Oral Implants Res, 12(6), 632-639. h5ps://doi.org/10.1034/j.1600-0501.2001.120612.x
9. Arenaz-Búa, J., Luaces-Rey, R., Sironvalle-Soliva, S., Otero-Rico, A., Charro-Huerga, E., PaBño-Seijas, B., García-Rozado, A., Ferreras-Granados, J., Vázquez-Mahía, I., Lorenzo-Franco, F., Maron-Sastre, R., & López-Cedrún, J. L. (2010). A comparaBve study of platelet-rich plasma, hydroxyapaBte, demineralized bone matrix and autologous bone to promote bone regeneraBon aFer mandibular impacted third molar extracBon. Med Oral Patol Oral Cir Bucal, 15(3), e483-489. h5ps://doi.org/10.4317/medoral.15.e483
10. ArRejaie, A., Al-Harbi, F., Alagl, A. S., & Hassan, K. S. (2016). Platelet-Rich Plasma Gel Combined with Bovine-Derived XenograF for the Treatment of Dehiscence Around Immediately Placed ConvenBonally Loaded Dental Implants in Humans: Cone Beam Computed Tomography and Three-Dimensional Image EvaluaBon. Int J Oral Maxillofac Implants, 31(2), 431-438. h5ps://doi.org/10.11607/jomi.3859
11. Arunjaroensuk, S., Panmekiate, S., & Pimkhaokham, A. (2018). The Stability of Augmented Bone Between Two Diﬀerent Membranes Used for Guided Bone RegeneraBon Simultaneous with Dental Implant Placement in the EstheBc Zone. Int J Oral Maxillofac Implants, 33(1), 206–216. h5ps://doi.org/10.11607/jomi.5492
12. Arunjaroensuk, S., Thunyakitpisal, P., Nampuksa, K., Monmaturapoj, N., Ma5heos, N., & Pimkhaokham, A. (2023). Stability of guided bone regeneraBon with two raBos of biphasic calcium phosphate at implant sites in the estheBc zone: A randomized controlled clinical trial. Clin Oral Implants Res, 34(8), 850-862. h5ps://doi.org/10.1111/clr.14113
13. Assenza, B., Pia5elli, M., Scarano, A., Lezzi, G., Petrone, G., & Pia5elli, A. (2001). Localized ridge augmentaBon using Btanium micromesh. J Oral Implantol, 27(6), 287-292. h5ps://doi.org/10.1563/1548-1336(2001)027<0287:Lrautm>2.3.Co;2
14. Atef, M., Osman, A. H., & Hakam, M. (2019). Autogenous interposiBonal block graF vs onlay graF for horizontal ridge augmentaBon in the mandible. Clin Implant Dent Relat Res, 21(4), 678-685. h5ps://doi.org/10.1111/cid.12809
15. Atef, M., Tarek, A., Shaheen, M., Alarawi, R. M., & Askar, N. (2020). Horizontal ridge augmentaBon using naBve collagen membrane vs Btanium mesh in atrophic maxillary ridges: Randomized clinical trial. Clin Implant Dent Relat Res, 22(2), 156-166. h5ps://doi.org/10.1111/cid.12892
16. Babbush, C. A. (2003). Histologic evaluaBon of human biopsies aFer dental augmentaBon with a demineralized bone matrix pu5y. Implant Dent, 12(4), 325-332. h5ps://doi.org/10.1097/01.id.0000095466.18042.31
17. Bahaa, S., Diab, N., Zazou, N., Darhous, M., El Arab, A. E., & ElNahass, H. (2023). EvaluaBon of bone gain in horizontal ridge augmentaBon using Btanium mesh in combinaBon with diﬀerent ﬂap advancement techniques: a randomized clinical trial. Int J Oral Maxillofac Surg, 52(3), 379-387. h5ps://doi.org/10.1016/j.ijom.2022.07.005
18. Barone, A., & Covani, U. (2007). Maxillary alveolar ridge reconstrucBon with nonvascularized autogenous block bone: clinical results. J Oral Maxillofac Surg, 65(10), 2039-2046. h5ps://doi.org/10.1016/j.joms.2007.05.017
19. Barone, A., Orlando, B., Cingano, L., Marconcini, S., Derchi, G., & Covani, U. (2012). A randomized clinical trial to evaluate and compare implants placed in augmented versus non-augmented extracBon sockets: 3-year results. J Periodontol, 83(7), 836-846. h5ps://doi.org/10.1902/jop.2011.110205
20. Bartols, A., Kasprzyk, S., Walther, W., & Korsch, M. (2018). Lateral alveolar ridge augmentaBon with autogenous block graFs ﬁxed at a distance versus resorbable Poly-D-L-LacBde foil ﬁxed at a distance: A single-blind, randomized, controlled trial. Clin Oral Implants Res, 29(8), 843-854. h5ps://doi.org/10.1111/clr.13303
21. Basler, T., Naenni, N., Schneider, D., Hämmerle, C. H. F., Jung, R. E., & Thoma, D. S. (2018). Randomized controlled clinical study assessing two membranes for guided bone regeneraBon of peri-implant bone defects: 3-year results. Clin Oral Implants Res, 29(5), 499-507. h5ps://doi.org/10.1111/clr.13147
22. Basma, H. S., Saleh, M. H. A., Geurs, N. C., Li, P., Ravidà, A., Wang, H. L., & Abou-Arraj, R. V. (2023). The eﬀect of bone parBcle size on the histomorphometric and clinical outcomes following lateral ridge augmentaBon procedures: A randomized double-blinded controlled trial. *J Periodontol*, 94(2), 163–173. h5ps://doi.org/10.1002/JPER.22-0212
23. Bechara, S., Kubilius, R., Veronesi, G., Pires, J. T., Shibli, J. A., & Mangano, F. G. (2017). Short (6-mm) dental implants versus sinus ﬂoor elevaBon and placement of longer (≥10-mm) dental implants: a randomized controlled trial with a 3-year follow-up. Clin Oral Implants Res, 28(9), 1097-1107. h5ps://doi.org/10.1111/clr.12923
24. Becker, W., Dahlin, C., Lekholm, U., Bergstrom, C., van Steenberghe, D., Higuchi, K., & Becker, B. E. (1999). Five-year evaluaBon of implants placed at extracBon and with dehiscences and fenestraBon defects augmented with ePTFE membranes: results from a prospecBve mulBcenter study. Clin Implant Dent Relat Res, 1(1), 27-32. h5ps://doi.org/10.1111/j.1708-8208.1999.tb00088.x
25. Benic, G. I., Bienz, S. P., Song, Y. W., Cha, J. K., Hämmerle, C. H. F., Jung, U. W., & Jung, R. E. (2022). Randomized controlled clinical trial comparing guided bone regeneraBon of peri-implant defects with soF-type block versus parBculate bone subsBtutes: Six-month results of hard-Bssue changes. J Clin Periodontol, 49(5), 480-495. h5ps://doi.org/10.1111/jcpe.13606
26. Bhatavadekar, N., Gandhi, Y., & Padhye, N. (2022). ComparaBve Assessment of Bovine Versus Porcine XenograF for AugmentaBon: A Randomized ProspecBve Cohort Study. Int J PeriodonBcs RestoraBve Dent, 42(6), 789-796. h5ps://doi.org/10.11607/prd.6025
27. Bienz, S. P., Payer, M., Hjerppe, J., Hüsler, J., Jakse, N., Schmidlin, P. R., Hämmerle, C. H. F., Jung, R. E., & Thoma,

D. S. (2021). Primary bone augmentaBon leads to equally stable marginal Bssue condiBons comparing the use of xenograF blocks infused with BMP-2 and autogenous bone blocks: A 3-year 3D analysis. *Clin Oral Implants Res*, 32(12), 1433–1443. h5ps://doi.org/10.1111/clr.13843

28. Bi5ner, N., Planzos, L., Volchonok, A., Tarnow, D., & Schulze-Späte, U. (2020). EvaluaBon of Horizontal and VerBcal Buccal Ridge Dimensional Changes AFer Immediate Implant Placement and Immediate TemporizaBon With and Without Bone AugmentaBon Procedures: Short-Term, 1-Year Results. A Randomized Controlled Clinical Trial. Int J PeriodonBcs RestoraBve Dent, 40(1), 83-93. h5ps://doi.org/10.11607/prd.4152
29. Bolle, C., Felice, P., Barausse, C., PisBlli, V., Trullenque-Eriksson, A., & Esposito, M. (2018). 4 mm long vs longer implants in augmented bone in posterior atrophic jaws: 1-year post-loading results from a mulBcentre randomised controlled trial. Eur J Oral Implantol, 11(1), 31-47.
30. Bouckaert, E., De Bruyckere, T., Eghbali, A., Younes, F., Wessels, R., & Cosyn, J. (2022). A randomized controlled trial comparing guided bone regeneraBon to connecBve Bssue graF to re-establish buccal convexity at dental implant sites: Three-year results. Clin Oral Implants Res, 33(5), 461-471. h5ps://doi.org/10.1111/clr.13906
31. Byun, S. H., Kim, S. Y., Lee, H., Lim, H. K., Kim, J. W., Lee, U. L., Lee, J. B., Park, S. H., Kim, S. J., Song, J. D., Jang, I.

S., Kim, M. K., & Kim, J. W. (2020). SoF Bssue expander for verBcally atrophied alveolar ridges: ProspecBve, mulBcenter, randomized controlled trial. Clin Oral Implants Res, 31(7), 585-594. h5ps://doi.org/10.1111/clr.13595

32. Canullo, L., & SisB, A. (2010). Early implant loading aFer verBcal ridge augmentaBon (VRA) using e-PTFE Btanium-reinforced membrane and nano-structured hydroxyapaBte: 2-year prospecBve study. Eur J Oral Implantol, 3(1), 59-69.
33. Carpio, L., Loza, J., Lynch, S., & Genco, R. (2000). Guided bone regeneraBon around endosseous implants with anorganic bovine bone mineral. A randomized controlled trial comparing bioabsorbable versus non-resorbable barriers. J Periodontol, 71(11), 1743-1749. h5ps://doi.org/10.1902/jop.2000.71.11.1743
34. Castagna, L., Polido, W. D., Soares, L. G., & Tinoco, E. M. (2013). Tomographic evaluaBon of iliac crest bone graFing and the use of immediate temporary implants to the atrophic maxilla. Int J Oral Maxillofac Surg, 42(9), 1067-1072. h5ps://doi.org/10.1016/j.ijom.2013.04.020
35. Cha, J. K., Jung, U. W., Montero-Solis, E., Sanz-Sánchez, I., & Sanz-Alonso, M. (2024). Guided bone regeneraBon at dehiscence comparing syntheBc bone subsBtute versus bovine bone mineral: A mulBcenter, noninferiority, randomized trial. Clin Implant Dent Relat Res, 26(6), 1233-1244. h5ps://doi.org/10.1111/cid.13386
36. Chiapasco, M., AbaB, S., Ramundo, G., Rossi, A., Romeo, E., & Vogel, G. (2000). Behavior of implants in bone graFs or free ﬂaps aFer tumor resecBon. Clin Oral Implants Res, 11(1), 66-75. h5ps://doi.org/10.1034/j.1600-0501.2000.011001066.x
37. Chiapasco, M., Romeo, E., CasenBni, P., & Rimondini, L. (2004). Alveolar distracBon osteogenesis vs. verBcal guided bone regeneraBon for the correcBon of verBcally deﬁcient edentulous ridges: a 1-3-year prospecBve study on humans. Clin Oral Implants Res, 15(1), 82-95. h5ps://doi.org/10.1111/j.1600-0501.2004.00999.x
38. Chiapasco, M., Zaniboni, M., & Rimondini, L. (2007). Autogenous onlay bone graFs vs. alveolar distracBon osteogenesis for the correcBon of verBcally deﬁcient edentulous ridges: a 2-4-year prospecBve study on humans. Clin Oral Implants Res, 18(4), 432-440. h5ps://doi.org/10.1111/j.1600-0501.2007.01351.x
39. Christensen, D. K., Karoussis, I. K., Joss, A., Hämmerle, C. H., & Lang, N. P. (2003). Simultaneous or staged installaBon with guided bone augmentaBon of transmucosal Btanium implants. A 3-year prospecBve cohort study. Clin Oral Implants Res, 14(6), 680-686. h5ps://doi.org/10.1046/j.0905-7161.2003.00963.x
40. Cockerham, B. L., 3rd, Patel, A., Greenwell, H., Hill, M., Shumway, B., & Hsu, H. T. (2020). Ridge AugmentaBon Comparing a Cancellous Block AllograF to an OsteoinducBve Demineralized Bone Matrix AllograF: A Randomized, Controlled, Blinded Clinical Trial. Int J PeriodonBcs RestoraBve Dent, 40(4), 571-578. h5ps://doi.org/10.11607/prd.4324
41. Cordaro, L., Torsello, F., Morcavallo, S., & di Torresanto, V. M. (2011). Eﬀect of bovine bone and collagen membranes on healing of mandibular bone blocks: a prospecBve randomized controlled study. Clin Oral Implants Res, 22(10), 1145-1150. h5ps://doi.org/10.1111/j.1600-0501.2010.02093.x
42. Cornelini, R., Cangini, F., Martuscelli, G., & Wennström, J. (2004). Deproteinized bovine bone and biodegradable barrier membranes to support healing following immediate placement of transmucosal implants: a short-term controlled clinical trial. Int J PeriodonBcs RestoraBve Dent, 24(6), 555-563.
43. Covani, U., Cornelini, R., & Barone, A. (2008). Buccal bone augmentaBon around immediate implants with and without ﬂap elevaBon: a modiﬁed approach. Int J Oral Maxillofac Implants, 23(5), 841-846.
44. Cucchi, A., Beani, S., Ghensi, P., Fiorino, A., & Corinaldesi, G. (2023). VerBcal ridge augmentaBon with Ti-reinforced dense polytetraﬂuoroethylene (d-PTFE) membranes or Ti-meshes and collagen membranes: 3-year results of a randomized clinical trial. Clin Implant Dent Relat Res, 25(2), 352-369. h5ps://doi.org/10.1111/cid.13173
45. Cucchi, A., Marchiori, G., Sartori, M., Fini, M., Fiorino, A., DonaB, R., Corinaldesi, G., & Maglio, M. (2024). A 3D micro-CT assessment of composiBon and structure of bone Bssue aFer verBcal and horizontal alveolar ridge augmentaBon using CAD/CAM-customized Btanium mesh. Clin Oral Implants Res, 35(12), 1546-1559. h5ps://doi.org/10.1111/clr.14342
46. Cucchi, A., Sartori, M., Aldini, N. N., Vignudelli, E., & Corinaldesi, G. (2019). A Proposal of Pseudo-periosteum ClassiﬁcaBon AFer GBR by Means of Titanium-Reinforced d-PTFE Membranes or Titanium Meshes Plus Cross-Linked Collagen Membranes. Int J PeriodonBcs RestoraBve Dent, 39(4), e157-e165. h5ps://doi.org/10.11607/prd.e3598
47. Cucchi, A., Vignudelli, E., Fiorino, A., Pellegrino, G., & Corinaldesi, G. (2021). VerBcal ridge augmentaBon (VRA) with Ti-reinforced d-PTFE membranes or Ti meshes and collagen membranes: 1-year results of a randomized clinical trial. Clin Oral Implants Res, 32(1), 1-14. h5ps://doi.org/10.1111/clr.13673
48. Cucchi, A., Vignudelli, E., Franceschi, D., Randellini, E., Lizio, G., Fiorino, A., & Corinaldesi, G. (2021). VerBcal and horizontal ridge augmentaBon using customized CAD/CAM Btanium mesh with versus without resorbable membranes. A randomized clinical trial. Clin Oral Implants Res, 32(12), 1411-1424. h5ps://doi.org/10.1111/clr.13841
49. Cucchi, A., Vignudelli, E., Napolitano, A., Marchea, C., & Corinaldesi, G. (2017). EvaluaBon of complicaBon rates and verBcal bone gain aFer guided bone regeneraBon with non-resorbable membranes versus Btanium meshes and resorbable membranes. A randomized clinical trial. Clin Implant Dent Relat Res, 19(5), 821-832. h5ps://doi.org/10.1111/cid.12520
50. Cucchi, A., Vignudelli, E., Sartori, M., Parrilli, A., Aldini, N. N., & Corinaldesi, G. (2021). A microcomputed tomography analysis of bone Bssue aFer verBcal ridge augmentaBon with non-resorbable membranes versus resorbable membranes and Btanium mesh in humans. Int J Oral Implantol (Berl), 14(1), 25-38.
51. Davó, R., Felice, P., PisBlli, R., Barausse, C., MarB-Pages, C., Ferrer-Fuertes, A., Ippolito, D. R., & Esposito, M. (2018). Immediately loaded zygomaBc implants vs convenBonal dental implants in augmented atrophic maxillae: 1-year post-loading results from a mulBcentre randomised controlled trial. Eur J Oral Implantol, 11(2), 145-161.
52. De Angelis, N., Felice, P., Pellegrino, G., CamuraB, A., Gambino, P., & Esposito, M. (2011). Guided bone regeneraBon with and without a bone subsBtute at single post-extracBve implants: 1-year post-loading results from a pragmaBc mulBcentre randomised controlled trial. Eur J Oral Implantol, 4(4), 313-325.
53. De Bruyckere, T., Cosyn, J., Younes, F., Hellyn, J., Bekx, J., Cleymaet, R., & Eghbali, A. (2020). A randomized controlled study comparing guided bone regeneraBon with connecBve Bssue graF to re-establish buccal convexity: One-year aestheBc and paBent-reported outcomes. Clin Oral Implants Res, 31(6), 507-516. h5ps://doi.org/10.1111/clr.13587
54. De Bruyckere, T., Eeckhout, C., Eghbali, A., Younes, F., Vandekerckhove, P., Cleymaet, R., & Cosyn, J. (2018). A randomized controlled study comparing guided bone regeneraBon with connecBve Bssue graF to re-establish convexity at the buccal aspect of single implants: A one-year CBCT analysis. J Clin Periodontol, 45(11), 1375-1387. h5ps://doi.org/10.1111/jcpe.13006
55. de Campos, C. G., Francischone, C. E., Souza Picorelli Assis, N. M., Devito, K. L., & So5o-Maior, B. S. (2019). Neurosensory FuncBon and Implant Survival Rate Following Implant Placement With or Without an Interposed Bone GraF Between the Implant and Nerve: ProspecBve Clinical Trial. Int J Oral Maxillofac Implants, 33(6), 1450-1456. h5ps://doi.org/10.11607/jomi.7543
56. Deesricharoenkiat, N., Jansisyanont, P., Chuenchompoonut, V., Ma5heos, N., & Thunyakitpisal, P. (2022). The eﬀect of acemannan in implant placement with simultaneous guided bone regeneraBon in the aestheBc zone: a randomized controlled trial. Int J Oral Maxillofac Surg, 51(4), 535-544. h5ps://doi.org/10.1016/j.ijom.2021.07.017
57. Degidi, M., Artese, L., Rubini, C., Perroa, V., Iezzi, G., & Pia5elli, A. (2007). Microvessel density in sinus augmentaBon procedures using anorganic bovine bone and autologous bone: 3 months results. Implant Dent, 16(3), 317-325. h5ps://doi.org/10.1097/ID.0b013e3180de4c5f
58. Dowlatshahi, S., Chen, C. Y., Zigdon-Giladi, H., Horwitz, J., Ahn, C., Kim, D. M., & Machtei, E. E. (2022). Volumetric assessment of changes in the alveolar ridge dimension following guided bone regeneraBon using a

combinaBon freeze-dried bone allograF with collagen membrane or novel resorbable scaﬀold: A prospecBve two-center clinical trial. J Periodontol, 93(3), 343-353. h5ps://doi.org/10.1002/jper.21-0006

59. El Ebiary, S. O., Atef, M., Abdelaziz, M. S., & Khashaba, M. (2023). Guided immediate implant with and without using a mixture of autogenous and xeno bone graFs in the dental estheBc zone. A randomized clinical trial. BMC Res Notes, 16(1), 331. h5ps://doi.org/10.1186/s13104-023-06612-8
60. El Hadidy, M. S., Mounir, M., Abou-Elfetouh, A., & Barakat, A. (2018). Assessment of verBcal ridge augmentaBon and labial prominence using buccal versus palatal approaches for maxillary segmental sandwich osteotomy (inlay technique): A randomized clinical trial. Clin Implant Dent Relat Res, 20(5), 722-728. h5ps://doi.org/10.1111/cid.12653
61. Elaskary, A., Abdelrahman, H., Elsabagh, H. H., & El-Kimary, G. I. (2022). Does GraFing the Jumping Gap in Immediately Placed Anterior Implants Using VesBbular Socket Therapy Inﬂuence the Labial Bone Thickness? J Oral Maxillofac Surg, 80(8), 1398-1407. h5ps://doi.org/10.1016/j.joms.2022.05.001
62. ElAskary, A., Elfana, A., Meabed, M., Abd-ElWahab Radi, I., Akram, M., & Fawzy El-Sayed, K. (2022). Immediate implant placement uBlizing vesBbular socket therapy versus early implant placement with contour augmentaBon for rehabilitaBon of compromised extracBon sockets in the estheBc zone: A randomized controlled clinical trial. Clin Implant Dent Relat Res, 24(5), 559-568. h5ps://doi.org/10.1111/cid.13120
63. Elraee, L., Abdel Gaber, H. K., Elsayed, H. H., & Adel-Kha5ab, D. (2022). Autogenous denBn block versus bone block for horizontal alveolar ridge augmentaBon and staged implant placement: A randomized controlled clinical trial including histologic assessment. Clin Oral Implants Res, 33(7), 723-734. h5ps://doi.org/10.1111/clr.13936
64. Elsayed, A. O., Abdel-Rahman, F. H., Ahmed, W. M., & Tawﬁk, M. A. (2024). Clinical and Radiographic Outcomes of Autogenous Inlay GraF vs Autogenous Onlay GraF for Anterior Maxillary Horizontal Ridge AugmentaBon: A Randomized Control Clinical Study. J Contemp Dent Pract, 25(2), 107-113. h5ps://doi.org/10.5005/jp-journals-10024-3634
65. Elsheikh, H. A., Abdelsameaa, S. E., Elbahnasi, A. A., & Abdel-Rahman, F. H. (2023). Comparison between platelet rich ﬁbrin as space ﬁlling material versus xenograF and alloplasBc bone graFing materials in immediate implant placement: a randomized clinical trial. BMC Oral Health, 23(1), 977. h5ps://doi.org/10.1186/s12903-023-03678-5
66. Emam, H., Beheiri, G., Elsalanty, M., & Sharawy, M. (2011). Microcomputed tomographic and histologic analysis of anorganic bone matrix coupled with cell-binding pepBde suspended in sodium hyaluronate carrier aFer sinus augmentaBon: a clinical study. Int J Oral Maxillofac Implants, 26(3), 561-570.
67. Esposito, M., Barausse, C., PisBlli, R., SammarBno, G., Grandi, G., & Felice, P. (2015). Short implants versus bone augmentaBon for placing longer implants in atrophic maxillae: One-year post-loading results of a pilot randomised controlled trial. Eur J Oral Implantol, 8(3), 257-268.
68. Esposito, M., Cannizarro, G., Soardi, E., Pellegrino, G., PisBlli, R., & Felice, P. (2011). A 3-year post-loading report of a randomised controlled trial on the rehabilitaBon of posterior atrophic mandibles: short implants or longer implants in verBcally augmented bone? Eur J Oral Implantol, 4(4), 301-311.
69. Esposito, M., Davó, R., MarB-Pages, C., Ferrer-Fuertes, A., Barausse, C., PisBlli, R., Ippolito, D. R., & Felice, P. (2018). Immediately loaded zygomaBc implants vs convenBonal dental implants in augmented atrophic maxillae: 4 months post-loading results from a mulBcentre randomised controlled trial. Eur J Oral Implantol, 11(1), 11-28.
70. F, G. F. T., Cortes, A. R. G., Hernandez Vallejo, G., Cabrejos-Azama, J., Tamimi, F., & Torres, J. (2019). Clinical and radiographic outcomes of allogeneic block graFs for maxillary lateral ridge augmentaBon: A randomized clinical trial. Clin Implant Dent Relat Res, 21(5), 1087-1098. h5ps://doi.org/10.1111/cid.12834
71. Felice, P., Barausse, C., PisBlli, V., Pia5elli, M., Ippolito, D. R., & Esposito, M. (2018). Posterior atrophic jaws rehabilitated with prostheses supported by 6 mm long × 4 mm wide implants or by longer implants in augmented bone. 3-year post-loading results from a randomised controlled trial. Eur J Oral Implantol, 11(2), 175-187.
72. Felice, P., Cannizzaro, G., Checchi, V., Marchea, C., Pellegrino, G., Censi, P., & Esposito, M. (2009). VerBcal bone augmentaBon versus 7-mm-long implants in posterior atrophic mandibles. Results of a randomised controlled clinical trial of up to 4 months aFer loading. Eur J Oral Implantol, 2(1), 7-20.
73. Felice, P., Checchi, V., PisBlli, R., Scarano, A., Pellegrino, G., & Esposito, M. (2009). Bone augmentaBon versus 5-mm dental implants in posterior atrophic jaws. Four-month post-loading results from a randomised controlled clinical trial. Eur J Oral Implantol, 2(4), 267-281.
74. Felice, P., PisBlli, R., Pia5elli, M., Soardi, E., Corvino, V., & Esposito, M. (2012). Posterior atrophic jaws rehabilitated with prostheses supported by 5 x 5 mm implants with a novel nanostructured calcium-

incorporated Btanium surface or by longer implants in augmented bone. Preliminary results from a randomised controlled trial. Eur J Oral Implantol, 5(2), 149-161.

75. Felice, P., Soardi, E., Pellegrino, G., PisBlli, R., Marchea, C., Gessaroli, M., & Esposito, M. (2011). Treatment of the atrophic edentulous maxilla: short implants versus bone augmentaBon for placing longer implants. Five-month post-loading results of a pilot randomised controlled trial. Eur J Oral Implantol, 4(3), 191-202.
76. Ferrea, C., & RipamonB, U. (2002). Human segmental mandibular defects treated with naturally derived bone morphogeneBc proteins. J Craniofac Surg, 13(3), 434-444. h5ps://doi.org/10.1097/00001665-200205000-00014
77. Fontana, F., Santoro, F., Maiorana, C., Iezzi, G., Pia5elli, A., & Simion, M. (2008). Clinical and histologic evaluaBon of allogeneic bone matrix versus autogenous bone chips associated with Btanium-reinforced e-PTFE membrane for verBcal ridge augmentaBon: a prospecBve pilot study. Int J Oral Maxillofac Implants, 23(6), 1003-1012.
78. Fouad, W., Osman, A., Atef, M., & Hakam, M. (2018). Guided maxillary sinus ﬂoor elevaBon using deproteinized bovine bone versus graFless Schneiderian membrane elevaBon with simultaneous implant placement: Randomized clinical trial. Clin Implant Dent Relat Res, 20(3), 424-433. h5ps://doi.org/10.1111/cid.12601
79. Friedmann, A., Gissel, K., Soudan, M., Kleber, B. M., Pitaru, S., & Dietrich, T. (2011). Randomized controlled trial on lateral augmentaBon using two collagen membranes: morphometric results on mineralized Bssue compound. J Clin Periodontol, 38(7), 677-685. h5ps://doi.org/10.1111/j.1600-051X.2011.01738.x
80. Froum, S., Cho, S. C., Rosenberg, E., Rohrer, M., & Tarnow, D. (2002). Histological comparison of healing extracBon sockets implanted with bioacBve glass or demineralized freeze-dried bone allograF: a pilot study. J Periodontol, 73(1), 94-102. h5ps://doi.org/10.1902/jop.2002.73.1.94
81. Fu, J. H., Oh, T. J., Benavides, E., Rudek, I., & Wang, H. L. (2014). A randomized clinical trial evaluaBng the eﬃcacy of the sandwich bone augmentaBon technique in increasing buccal bone thickness during implant placement surgery: I. Clinical and radiographic parameters. Clin Oral Implants Res, 25(4), 458-467. h5ps://doi.org/10.1111/clr.12171
82. Fu, J. H., Rios, H., Al-Hezaimi, K., Oh, T. J., Benavides, E., & Wang, H. L. (2015). A randomized clinical trial evaluaBng the eﬃcacy of the sandwich bone augmentaBon technique in increasing buccal bone thickness

during implant placement. II. Tomographic, histologic, immunohistochemical, and RNA analyses. Clin Oral Implants Res, 26(10), 1150-1157. h5ps://doi.org/10.1111/clr.12481

83. Gaballah, O., & Abd-Elwahab Radi, I. (2019). Limited Evidence Suggests Guided Bone RegeneraBon With or Without Autogenous Bone GraFs are Equivalently EﬀecBve in Horizontal Bone Gain. J Evid Based Dent Pract, 19(4), 101351. h5ps://doi.org/10.1016/j.jebdp.2019.101351
84. Gaggl, A., Schultes, G., & Kärcher, H. (2000). VerBcal alveolar ridge distracBon with prostheBc treatable distractors: a clinical invesBgaBon. Int J Oral Maxillofac Implants, 15(5), 701-710.
85. Gallo, P., Díaz-Báez, D., Perdomo, S., Aloise, A. C., Ta5an, M., Saleh, M. H. A., Pelegrine, A. A., Ravidà, A., & Wang, H. L. (2022). ComparaBve analysis of two biomaterials mixed with autogenous bone graF for verBcal ridge augmentaBon: A histomorphometric study in humans. Clin Implant Dent Relat Res, 24(5), 709-719. h5ps://doi.org/10.1111/cid.13124
86. Garoushi, I. H., Elbeialy, R. R., Gibaly, A., & Atef, M. (2021). EvaluaBon of the eﬀect of the lateralized inferior alveolar nerve isolaBon and bone graFing on the nerve funcBon and implant stability. (Randomized Clinical Trial). Clin Implant Dent Relat Res, 23(3), 423-431. h5ps://doi.org/10.1111/cid.13003
87. Gher, M. E., Quintero, G., Assad, D., Monaco, E., & Richardson, A. C. (1994). Bone graFing and guided bone regeneraBon for immediate dental implants in humans. J Periodontol, 65(9), 881-891. h5ps://doi.org/10.1902/jop.1994.65.9.881
88. Gher, M. E., Quintero, G., Sandifer, J. B., Tabacco, M., & Richardson, A. C. (1994). Combined dental implant and guided Bssue regeneraBon therapy in humans. Int J PeriodonBcs RestoraBve Dent, 14(4), 332-347.
89. Giragosyan, K., Chenchev, I., & Ivanova, V. (2024). Linear bone gain and healing complicaBon rate comparaBve outcomes following ridge augmentaBon with custom 3D printed Btanium mesh vs Ti-reinforced dPTFE. A randomized clinical trial. Folia Med (Plovdiv), 66(4), 505-514. h5ps://doi.org/10.3897/folmed.66.e123766
90. Gjerde, C., Mustafa, K., Hellem, S., Rojewski, M., Gjengedal, H., Yassin, M. A., Feng, X., Skaale, S., Berge, T., Rosen, A., Shi, X. Q., Ahmed, A. B., Gjertsen, B. T., Schrezenmeier, H., & Layrolle, P. (2018). Cell therapy induced regeneraBon of severely atrophied mandibular bone in a clinical trial. Stem Cell Res Ther, 9(1), 213. h5ps://doi.org/10.1186/s13287-018-0951-9
91. Gunne, J., Nyström, E., & Kahnberg, K. E. (1995). Bone graFs and implants in the treatment of the severely resorbed maxillae: a 3-year follow-up of the prostheBc restoraBon. Int J Prosthodont, 8(1), 38-45.
92. Hanser, T., & Doliveux, R. (2018). MicroSaw and Piezosurgery in HarvesBng Mandibular Bone Blocks from the Retromolar Region: A Randomized Split-Mouth ProspecBve Clinical Trial. Int J Oral Maxillofac Implants, 33(2), 365-372. h5ps://doi.org/10.11607/jomi.4416
93. Hartlev, J., Schou, S., Isidor, F., & Nørholt, S. E. (2021). A clinical and radiographic study of implants placed in autogenous bone graFs covered by either a platelet-rich ﬁbrin membrane or deproteinised bovine bone mineral and a collagen membrane: a pilot randomised controlled clinical trial with a 2-year follow-up. Int J Implant Dent, 7(1), 8. h5ps://doi.org/10.1186/s40729-021-00289-z
94. Hartlev, J., Spin-Neto, R., Schou, S., Isidor, F., & Nørholt, S. E. (2019). Cone beam computed tomography evaluaBon of staged lateral ridge augmentaBon using platelet-rich ﬁbrin or resorbable collagen membranes in a randomized controlled clinical trial. Clin Oral Implants Res, 30(3), 277-284. h5ps://doi.org/10.1111/clr.13413
95. Hashemipoor, M., Asghari, N., Mohammadi, M., Kalantari, M., Arabsolghar, M., & Ranjbar, H. (2020). Radiological and histological evaluaBon of horizontal ridge augmentaBon using corBcocancellous freeze-dried bone allograF with and without autogenous bone: A randomized controlled clinical trial. Clin Implant Dent Relat Res, 22(5), 582-592. h5ps://doi.org/10.1111/cid.12935
96. Hassan, K. S. (2009). Autogenous bone graF combined with polylacBc polyglycolic acid polymer for treatment of dehiscence around immediate dental implants. Oral Surg Oral Med Oral Pathol Oral Radiol Endod, 108(5), e19-25. h5ps://doi.org/10.1016/j.tripleo.2009.07.023
97. Hassan, K. S., Kassim, A., & Al Ogaly, A. U. (2008). A comparaBve evaluaBon of immediate dental implant with autogenous versus syntheBc guided bone regeneraBon. Oral Surg Oral Med Oral Pathol Oral Radiol Endod, 106(5), e8-e15. h5ps://doi.org/10.1016/j.tripleo.2008.07.015
98. Hazzaa, H. H. A., El-Kilani, N. S., Elsayed, S. A., & Abd El Massieh, P. M. (2019). EvaluaBon of Immediate Implants Augmented with Autogenous Bone/Melatonin Composite GraF in the EstheBc Zone: A Randomized Controlled Trial. J Prosthodont, 28(2), e637-e642. h5ps://doi.org/10.1111/jopr.12631
99. Hernández-Alfaro, F., Pages, C. M., García, E., Corchero, G., & Arranz, C. (2005). Palatal core graF for alveolar reconstrucBon: a new donor site. Int J Oral Maxillofac Implants, 20(5), 777-783.
100. Işık, G., Özden Yüce, M., Koçak-Topbaş, N., & Günbay, T. (2021). Guided bone regeneraBon simultaneous with implant placement using bovine-derived xenograF with and without liquid platelet-rich ﬁbrin: a randomized controlled clinical trial. Clin Oral InvesBg, 25(9), 5563-5575. h5ps://doi.org/10.1007/s00784-021-03987-5
101. Issa, D. R., Elamrousy, W., & Gamal, A. Y. (2024). Alveolar ridge spliang and simvastaBn loaded xenograF for guided bone regeneraBon and simultaneous implant placement: randomized controlled clinical trial. Clin Oral InvesBg, 28(1), 71. h5ps://doi.org/10.1007/s00784-023-05427-y
102. Johansson, B., Bäck, T., & Hirsch, J. M. (2004). Cuang torque measurements in conjuncBon with implant placement in graFed and nongraFed maxillas as an objecBve evaluaBon of bone density: a possible method for idenBfying early implant failures? Clin Implant Dent Relat Res, 6(1), 9-15. h5ps://doi.org/10.1111/j.1708-8208.2004.tb00022.x
103. Jonker, B. P., Wolvius, E. B., van der Tas, J. T., & Pijpe, J. (2018). The eﬀect of resorbable membranes on one-stage ridge augmentaBon in anterior single-tooth replacement: A randomized, controlled clinical trial. Clin Oral Implants Res, 29(2), 235-247. h5ps://doi.org/10.1111/clr.13106
104. Jonker, B. P., Wolvius, E. B., van der Tas, J. T., Tahmaseb, A., & Pijpe, J. (2020). EstheBcs and PaBent-Reported Outcomes of Implants Placed with Guided Bone RegeneraBon and Complete NaBve Bone: A ProspecBve Controlled Clinical Trial. Int J Oral Maxillofac Implants, 35(2), 406-414. h5ps://doi.org/10.11607/jomi.7751
105. Jung, R. E., Benic, G. I., Scherrer, D., & Hämmerle, C. H. (2015). Cone beam computed tomography evaluaBon of regenerated buccal bone 5 years aFer simultaneous implant placement and guided bone regeneraBon procedures--a randomized, controlled clinical trial. Clin Oral Implants Res, 26(1), 28-34. h5ps://doi.org/10.1111/clr.12296
106. Jung, R. E., Brügger, L. V., Bienz, S. P., Hüsler, J., Hämmerle, C. H. F., & Zitzmann, N. U. (2021). Clinical and radiographical performance of implants placed with simultaneous guided bone regeneraBon using resorbable and nonresorbable membranes aFer 22-24 years, a prospecBve, controlled clinical trial. Clin Oral Implants Res, 32(12), 1455-1465. h5ps://doi.org/10.1111/clr.13845
107. Jung, R. E., Hälg, G. A., Thoma, D. S., & Hämmerle, C. H. (2009). A randomized, controlled clinical trial to evaluate a new membrane for guided bone regeneraBon around dental implants. Clin Oral Implants Res, 20(2), 162-168. h5ps://doi.org/10.1111/j.1600-0501.2008.01634.x
108. Jung, R. E., Herzog, M., Wolleb, K., Ramel, C. F., Thoma, D. S., & Hämmerle, C. H. (2017). A randomized controlled clinical trial comparing small buccal dehiscence defects around dental implants treated with guided bone regeneraBon or leF for spontaneous healing. Clin Oral Implants Res, 28(3), 348-354. h5ps://doi.org/10.1111/clr.12806
109. Jung, R. E., Kovacs, M. N., Thoma, D. S., & Hämmerle, C. H. F. (2022). InformaBve Btle: Guided bone regeneraBon with and without rhBMP-2: 17-year results of a randomized controlled clinical trial. Clin Oral Implants Res, 33(3), 302-312. h5ps://doi.org/10.1111/clr.13889
110. Jung, R. E., Windisch, S. I., Eggenschwiler, A. M., Thoma, D. S., Weber, F. E., & Hämmerle, C. H. (2009). A randomized-controlled clinical trial evaluaBng clinical and radiological outcomes aFer 3 and 5 years of dental implants placed in bone regenerated by means of GBR techniques with or without the addiBon of BMP-2. Clin Oral Implants Res, 20(7), 660-666. h5ps://doi.org/10.1111/j.1600-0501.2008.01648.x
111. Kaner, D., & Friedmann, A. (2011). SoF Bssue expansion with self-ﬁlling osmoBc Bssue expanders before verBcal ridge augmentaBon: a proof of principle study. J Clin Periodontol, 38(1), 95-101. h5ps://doi.org/10.1111/j.1600-051X.2010.01630.x
112. Kaamani, V. S., Chakravarthi, S. P., Neelima Devi, K. N., Sridhar, M. S., & Prasad, L. K. (2014). ComparaBve evaluaBon of bovine derived hydroxyapaBte and syntheBc hydroxyapaBte graF in bone regeneraBon of human maxillary cysBc defects: a clinico-radiological study. Indian J Dent Res, 25(5), 594-601. h5ps://doi.org/10.4103/0970-9290.147100
113. Kim, N. H., Yang, B. E., On, S. W., Kwon, I. J., Ahn, K. M., Lee, J. H., & Byun, S. H. (2024). Customized three-dimensional printed ceramic bone graFs for osseous defects: a prospecBve randomized study. Sci Rep, 14(1), 3397. h5ps://doi.org/10.1038/s41598-024-53686-w
114. Kohal, R. J., Straub, L. M., Wolkewitz, M., Bächle, M., & Patzelt, S. B. (2015). EvaluaBon of Guided Bone RegeneraBon around Oral Implants over Diﬀerent Healing Times Using Two Diﬀerent Bovine Bone Materials: A Randomized, Controlled Clinical and Histological InvesBgaBon. Clin Implant Dent Relat Res, 17(5), 957-971. h5ps://doi.org/10.1111/cid.12206
115. Korsch, M., Kasprzyk, S., Walther, W., & Bartols, A. (2019). Lateral alveolar ridge augmentaBon with autogenous block graFs ﬁxed at a distance vs resorbable poly-D-L-lacBde foil ﬁxed at a distance: 5-year results of a single-blind, randomised controlled trial. Int J Oral Implantol (Berl), 12(3), 299-312.
116. Kramer, F. J., Dempf, R., & Bremer, B. (2005). Eﬃcacy of dental implants placed into ﬁbula-free ﬂaps for orofacial reconstrucBon. Clin Oral Implants Res, 16(1), 80-88. h5ps://doi.org/10.1111/j.1600-0501.2004.01040.x
117. Krasny, M., Krasny, K., Fiedor, P., Zadurska, M., & Kamiński, A. (2015). Long-term outcomes of the use of allogeneic, radiaBon-sterilised bone blocks in reconstrucBon of the atrophied alveolar ridge in the maxilla and mandible. Cell Tissue Bank, 16(4), 631-638. h5ps://doi.org/10.1007/s10561-015-9512-y
118. Leong, D. J., Oh, T. J., Benavides, E., Al-Hezaimi, K., Misch, C. E., & Wang, H. L. (2015). Comparison between sandwich bone augmentaBon and allogenic block graF for verBcal ridge augmentaBon in the posterior mandible. Implant Dent, 24(1), 4-12. h5ps://doi.org/10.1097/id.0000000000000180
119. Li, P., Zhu, H., & Huang, D. (2018). Autogenous DDM versus Bio-Oss granules in GBR for immediate implantaBon in periodontal postextracBon sites: A prospecBve clinical study. Clin Implant Dent Relat Res, 20(6), 923-928. h5ps://doi.org/10.1111/cid.12667
120. Lima, R. G., Lima, T. G., Francischone, C. E., Turssi, C., Souza Picorelli Assis, N. M., & So5o-Maior, B. S. (2018). Bone Volume Dynamics and Implant Placement Torque in Horizontal Bone Defects Reconstructed with Autologous or Xenogeneic Block Bone: A Randomized, Controlled, Split-Mouth, ProspecBve Clinical Trial. Int J Oral Maxillofac Implants, 33(4), 888-894. h5ps://doi.org/10.11607/jomi.6288
121. Lumea, S., Consolo, U., Galli, C., MulBnu, A., PiersanB, L., Bellini, P., Manfredi, E., Corinaldesi, G., Zaﬀe, D., Macaluso, G. M., & Marchea, C. (2014). Fresh-frozen bone blocks for horizontal ridge augmentaBon in the upper maxilla: 6-month outcomes of a randomized controlled trial. Clin Implant Dent Relat Res, 16(1), 116-123. h5ps://doi.org/10.1111/j.1708-8208.2012.00458.x
122. Lundgren, S., Rasmusson, L., Sjöström, M., & Sennerby, L. (1999). Simultaneous or delayed placement of Btanium implants in free autogenous iliac bone graFs. Histological analysis of the bone graF-Btanium interface in 10 consecuBve paBents. Int J Oral Maxillofac Surg, 28(1), 31-37.
123. Mahmoud, Z. T., Wainwright, M., & Troedhan, A. (2020). Flapless Piezotome Crest Split Achieves Comparable Outcomes to Autologous Onlay GraFs With Signiﬁcant Less PaBent Morbidity and ComplicaBons-A Randomized Clinical Study. J Oral Maxillofac Surg, 78(11), 1953-1964. h5ps://doi.org/10.1016/j.joms.2020.06.008
124. Maiorana, C., Bere5a, M., Salina, S., & Santoro, F. (2005). ReducBon of autogenous bone graF resorpBon by means of bio-oss coverage: a prospecBve study. Int J PeriodonBcs RestoraBve Dent, 25(1), 19-25.
125. Mastrangelo, F., Gastaldi, G., Vinci, R., Troiano, G., Te5amanB, L., Gherlone, E., & Lo Muzio, L. (2018). Immediate PostextracBve Implants With and Without Bone GraF: 3-year Follow-up Results From a MulBcenter Controlled Randomized Trial. Implant Dent, 27(6), 638-645. h5ps://doi.org/10.1097/id.0000000000000816
126. Mau, J. L., Grodin, E., Lin, J. J., Chen, M. C., Ho, C. H., & Cochran, D. (2019). A comparaBve, randomized, prospecBve, two-center clinical study to evaluate the clinical and estheBc outcomes of two diﬀerent bone graFing techniques in early implant placement. J Periodontol, 90(3), 247-255. h5ps://doi.org/10.1002/jper.17-0491
127. Mazzocco, F., Nart, J., Cheung, W. S., & Griﬃn, T. J. (2011). ProspecBve evaluaBon of the use of motorized ridge expanders in guided bone regeneraBon for future implant sites. Int J PeriodonBcs RestoraBve Dent, 31(5), 547-

554. \

128. Mehlisch, D. R. (1989). Collagen/hydroxylapaBte implant for augmenBng deﬁcient alveolar ridges: a 24-month clinical and histologic summary. Oral Surg Oral Med Oral Pathol, 68(4 Pt 2), 505-514, discussion 514. h5ps://doi.org/10.1016/0030-4220(89)90230-2
129. Mehlisch, D. R., Taylor, T. D., Leibold, D. G., Hia5, R., Waite, D. E., Waite, P. D., Laskin, D. M., & Smith, S. T. (1988). Collagen/hydroxylapaBte implant for augmenBng deﬁcient alveolar ridges: twelve-month clinical data. J Oral Maxillofac Surg, 46(10), 839-843. h5ps://doi.org/10.1016/0278-2391(88)90046-8
130. Meijndert, L., Meijer, H. J., Stellingsma, K., Stegenga, B., & Raghoebar, G. M. (2007). EvaluaBon of aestheBcs of implant-supported single-tooth replacements using diﬀerent bone augmentaBon procedures: a prospecBve randomized clinical study. Clin Oral Implants Res, 18(6), 715-719. h5ps://doi.org/10.1111/j.1600-0501.2007.01415.x
131. Meijndert, L., Raghoebar, G. M., Meijer, H. J., & Vissink, A. (2008). Clinical and radiographic characterisBcs of single-tooth replacements preceded by local ridge augmentaBon: a prospecBve randomized clinical trial. Clin Oral Implants Res, 19(12), 1295-1303. h5ps://doi.org/10.1111/j.1600-0501.2008.01523.x
132. Meloni, S. M., Jovanovic, S. A., Urban, I., Canullo, L., Pisano, M., & Tallarico, M. (2017). Horizontal Ridge AugmentaBon using GBR with a NaBve Collagen Membrane and 1:1 RaBo of ParBculated XenograF and

Autologous Bone: A 1-Year ProspecBve Clinical Study. Clin Implant Dent Relat Res, 19(1), 38-45. h5ps://doi.org/10.1111/cid.12429

133. Mendoza-Azpur, G., de la Fuente, A., Chavez, E., Valdivia, E., & Khouly, I. (2019). Horizontal ridge augmentaBon with guided bone regeneraBon using parBculate xenogenic bone subsBtutes with or without autogenous block graFs: A randomized controlled trial. Clin Implant Dent Relat Res, 21(4), 521-530. h5ps://doi.org/10.1111/cid.12740
134. Merli, M., Lombardini, F., & Esposito, M. (2010). VerBcal ridge augmentaBon with autogenous bone graFs 3 years aFer loading: resorbable barriers versus Btanium-reinforced barriers. A randomized controlled clinical trial. Int J Oral Maxillofac Implants, 25(4), 801-807.
135. Merli, M., Moscatelli, M., Marioa, G., Pagliaro, U., Breschi, L., Mazzoni, A., & Nieri, M. (2015). Membranes and Bone SubsBtutes in a One-Stage Procedure for Horizontal Bone AugmentaBon: A Histologic Double-Blind Parallel Randomized Controlled Trial. Int J PeriodonBcs RestoraBve Dent, 35(4), 463-471. h5ps://doi.org/10.11607/prd.2418
136. Merli, M., Moscatelli, M., Marioa, G., Pagliaro, U., Raﬀaelli, E., & Nieri, M. (2015). Comparing membranes and bone subsBtutes in a one-stage procedure for horizontal bone augmentaBon. A double-blind randomised controlled trial. Eur J Oral Implantol, 8(3), 271-281.
137. Merli, M., Moscatelli, M., Marioa, G., Pagliaro, U., Raﬀaelli, E., & Nieri, M. (2018). Comparing membranes and bone subsBtutes in a one-stage procedure for horizontal bone augmentaBon. Three-year post-loading results of a double-blind randomised controlled trial. Eur J Oral Implantol, 11(4), 441-452.
138. Merli, M., Moscatelli, M., Marioa, G., Rotundo, R., Bernardelli, F., & Nieri, M. (2014). Bone level variaBon aFer verBcal ridge augmentaBon: resorbable barriers versus Btanium-reinforced barriers. A 6-year double-blind randomized clinical trial. Int J Oral Maxillofac Implants, 29(4), 905-913. h5ps://doi.org/10.11607/jomi.3203
139. Merli, M., Nieri, M., Marioa, G., Merli, M., Franchi, L., & Quiroga Souki, B. (2020). The fence technique: Autogenous bone graF versus 50% deproteinized bovine bone matrix / 50% autogenous bone graF-A clinical double-blind randomized controlled trial. Clin Oral Implants Res, 31(12), 1223-1231. h5ps://doi.org/10.1111/clr.13670
140. Morad, G., & Khojasteh, A. (2013). CorBcal tenBng technique versus onlay layered technique for verBcal augmentaBon of atrophic posterior mandibles: a split-mouth pilot study. Implant Dent, 22(6), 566-571. h5ps://doi.org/10.1097/01.id.0000433590.33926.af
141. Mordenfeld, A., Johansson, C. B., Albrektsson, T., & Hallman, M. (2014). A randomized and controlled clinical trial of two diﬀerent composiBons of deproteinized bovine bone and autogenous bone used for lateral ridge augmentaBon. Clin Oral Implants Res, 25(3), 310-320. h5ps://doi.org/10.1111/clr.12143
142. Mounir, M., Morsy, O. A. E., Amer, H., Mounir, S., & Gibaly, A. (2021). Assessment of bone quality using buccal and palatal autogenous corBcal shells harvested from two diﬀerent mandibular donor sites for maxillary alveolar ridge augmentaBon: a histomorphometric randomized clinical trial. Oral Maxillofac Surg, 25(2), 263-

269. h5ps://doi.org/10.1007/s10006-020-00924-w

143. Mounir, M., Mounir, S., Abou-Elfetouh, A., & Shaker, M. A. (2017). Assessment of verBcal ridge augmentaBon in anterior aestheBc zone using onlay xenograFs with Btanium mesh versus the inlay bone graFing technique: A randomized clinical trial. Int J Oral Maxillofac Surg, 46(11), 1458-1465. h5ps://doi.org/10.1016/j.ijom.2017.04.021
144. Naenni, N., Stucki, L., Hüsler, J., Schneider, D., Hämmerle, C. H. F., Jung, R. E., & Thoma, D. S. (2021). Implants sites with concomitant bone regeneraBon using a resorbable or non-resorbable membrane result in stable marginal bone levels and similar proﬁlometric outcomes over 5 years. Clin Oral Implants Res, 32(8), 893-904. h5ps://doi.org/10.1111/clr.13764
145. Nagata, M., Hoshina, H., Li, M., Arasawa, M., Uematsu, K., Ogawa, S., Yamada, K., Kawase, T., Suzuki, K., Ogose, A., Fuse, I., Okuda, K., Uoshima, K., Nakata, K., Yoshie, H., & Takagi, R. (2012). A clinical study of alveolar bone Bssue engineering with cultured autogenous periosteal cells: coordinated acBvaBon of bone formaBon and resorpBon. Bone, 50(5), 1123-1129. h5ps://doi.org/10.1016/j.bone.2012.02.631
146. Nam, J. W., Khureltogtokh, S., Choi, H. M., Lee, A. R., Park, Y. B., & Kim, H. J. (2017). Randomised controlled clinical trial of augmentaBon of the alveolar ridge using recombinant human bone morphogeneBc protein 2 with hydroxyapaBte and bovine-derived xenograFs: comparison of changes in volume. Br J Oral Maxillofac Surg, 55(8), 822-829. h5ps://doi.org/10.1016/j.bjoms.2017.07.017
147. Nemcovsky, C. E., & Artzi, Z. (2002). ComparaBve study of buccal dehiscence defects in immediate, delayed, and late maxillary implant placement with collagen membranes: clinical healing between placement and second-stage surgery. J Periodontol, 73(7), 754-761. h5ps://doi.org/10.1902/jop.2002.73.7.754
148. Nissan, J., Gross, O., Mardinger, O., Ghelfan, O., Sacco, R., & Chaushu, G. (2011). Post-traumaBc implant-supported restoraBon of the anterior maxillary teeth using cancellous bone block allograFs. J Oral Maxillofac Surg, 69(12), e513-518. h5ps://doi.org/10.1016/j.joms.2011.08.002
149. Oguić, M., Čandrlić, M., Tomas, M., Vidaković, B., Blašković, M., Jerbić RadeBć, A. T., Zoričić Cvek, S., Kuiš, D., & Cvijanović Peloza, O. (2023). Osteogenic PotenBal of Autologous DenBn GraF Compared with Bovine XenograF Mixed with Autologous Bone in the EstheBc Zone: Radiographic, Histologic and Immunohistochemical EvaluaBon. Int J Mol Sci, 24(7). h5ps://doi.org/10.3390/ijms24076440
150. OrBz-Vigón, A., Suarez, I., Maronez-Villa, S., Sanz-Maron, I., Bollain, J., & Sanz, M. (2018). Safety and performance of a novel collagenated xenogeneic bone block for lateral alveolar crest augmentaBon for staged implant placement. Clin Oral Implants Res, 29(1), 36-45. h5ps://doi.org/10.1111/clr.13036
151. Ouyang, L., Li, J., Dong, Y., Li, J., Jin, F., Luo, Y., Wang, R., & Wang, S. (2024). Comparison of clinical eﬃcacy between autologous parBally demineralized denBn matrix and deproteinized bovine bone mineral for bone augmentaBon in orthodonBc paBents with alveolar bone deﬁciency: a randomized controlled clinical trial. BMC Oral Health, 24(1), 984. h5ps://doi.org/10.1186/s12903-024-04730-8
152. Özkan, Y., Özcan, M., Varol, A., Akoglu, B., Ucankale, M., & Basa, S. (2007). Resonance frequency analysis assessment of implant stability in labial onlay graFed posterior mandibles: A pilot clinical study. *Int J Oral Maxillofac Implants*, 22(2), 235–242. h5ps://doi.org/10.1053/jomi.2007.22.2.235
153. Park, S. H., & Wang, H. L. (2007). Clinical signiﬁcance of incision locaBon on guided bone regeneraBon: human study. J Periodontol, 78(1), 47-51. h5ps://doi.org/10.1902/jop.2007.060125
154. Patel, A., Greenwell, H., Hill, M., Shumway, B., & Radmall, A. (2019). Ridge AugmentaBon Comparing an AllograF Plus Autogenous Bone Chips to an OsteoinducBve Demineralized Bone Matrix: A Clinical and Histologic Study in Humans. Implant Dent, 28(6), 613-620. h5ps://doi.org/10.1097/id.0000000000000925
155. Pia5elli, A., Degidi, M., Di Stefano, D. A., Rubini, C., Fioroni, M., & Strocchi, R. (2002). Microvessel density in alveolar ridge regeneraBon with autologous and alloplasBc bone. Implant Dent, 11(4), 370-375. h5ps://doi.org/10.1097/00008505-200211040-00017
156. Pia5elli, M., Scarano, A., & Pia5elli, A. (1996). Histological evaluaBon of freeze-dried dura mater (FDDMA) used in guided bone regeneraBon (GBR): a Bme course study in man. Biomaterials, 17(24), 2319-2323. h5ps://doi.org/10.1016/s0142-9612(96)00068-3
157. Pieri, F., Corinaldesi, G., Fini, M., Aldini, N. N., Giardino, R., & Marchea, C. (2008). Alveolar ridge augmentaBon with Btanium mesh and a combinaBon of autogenous bone and anorganic bovine bone: a 2-year prospecBve study. J Periodontol, 79(11), 2093-2103. h5ps://doi.org/10.1902/jop.2008.080061
158. Pinholt, E. M. (2003). Brånemark and ITI dental implants in the human bone-graFed maxilla: a comparaBve evaluaBon. Clin Oral Implants Res, 14(5), 584-592. h5ps://doi.org/10.1034/j.1600-0501.2003.140508.x
159. PisBlli, R., Felice, P., Piatelli, M., Nisii, A., Barausse, C., & Esposito, M. (2014). Blocks of autogenous bone versus xenograFs for the rehabilitaBon of atrophic jaws with dental implants: preliminary data from a pilot randomised controlled trial. Eur J Oral Implantol, 7(2), 153-171.
160. Proussaefs, P., Lozada, J., Kleinman, A., & Rohrer, M. D. (2002). The use of ramus autogenous block graFs for verBcal alveolar ridge augmentaBon and implant placement: a pilot study. Int J Oral Maxillofac Implants, 17(2), 238-248.
161. Qiu, L., & Yu, H. (2018). Onlay graFing with bovine bone mineral block for horizontal reconstrucBon of severely atrophic alveolar ridges in anterior maxillae: A 6-year prospecBve study. J Craniomaxillofac Surg, 46(8), 1199-1204. h5ps://doi.org/10.1016/j.jcms.2018.04.004
162. Ragab Mahmoud, A. E., Mohammad Yassin, S., Ali Hassan, S., & Sayed Abdelmoneim, H. (2024). VerBcal alveolar bone augmentaBon of atrophied posterior mandibular regions with simultaneous dental implant placement using allogeneic bone rings vs autogenous bone rings: a randomized controlled clinical trial. Quintessence Int, 55(3), 232-243. h5ps://doi.org/10.3290/j.qi.b4867849
163. Ramel, C. F., Wismeijer, D. A., Hämmerle, C. H., & Jung, R. E. (2012). A randomized, controlled clinical evaluaBon of a syntheBc gel membrane for guided bone regeneraBon around dental implants: clinical and radiologic 1- and 3-year results. Int J Oral Maxillofac Implants, 27(2), 435-441.
164. Rasmusson, L., Thor, A., & Sennerby, L. (2012). Stability evaluaBon of implants integrated in graFed and nongraFed maxillary bone: a clinical study from implant placement to abutment connecBon. Clin Implant Dent Relat Res, 14(1), 61-66. h5ps://doi.org/10.1111/j.1708-8208.2010.00239.x
165. Rocchie5a, I., Simion, M., Hoﬀmann, M., Trisciuoglio, D., Benigni, M., & Dahlin, C. (2016). VerBcal Bone AugmentaBon with an Autogenous Block or ParBcles in CombinaBon with Guided Bone RegeneraBon: A Clinical and Histological Preliminary Study in Humans. Clin Implant Dent Relat Res, 18(1), 19-29. h5ps://doi.org/10.1111/cid.12267
166. Romito, G. A., Fonseca, M. A., Soares, H. H., de Oliveira Lazarin, R., Sapata, V. M., Nishyama, R., Conde, M. C., Hammerle, C. H. F., Schwarz, F., & Villar, C. C. (2024). Clinical outcomes following atrophic alveolar ridge reconstrucBon using collagenated xenogeneic bone block or autogenous bone block: One-year follow-up of a randomized controlled clinical. J Clin Periodontol, 51(1), 14-23. h5ps://doi.org/10.1111/jcpe.13891
167. Romito, G. A., Soares, H. H., do Amaral, G., Fonseca, M. A., Sapata, V. M., Conde, M. C., Hammerle, C. H. F., Schwarz, F., & Villar, C. C. (2023). Radiographic outcomes of ridge reconstrucBon with autogenous bone block versus collagenated xenogeneic bone block: A randomized clinical trial. Clin Oral Implants Res, 34(8), 863-871. h5ps://doi.org/10.1111/clr.14114
168. Ronda, M., Rebaudi, A., Torelli, L., & Stacchi, C. (2014). Expanded vs. dense polytetraﬂuoroethylene membranes in verBcal ridge augmentaBon around dental implants: a prospecBve randomized controlled clinical trial. Clin Oral Implants Res, 25(7), 859-866. h5ps://doi.org/10.1111/clr.12157
169. Santana, R. B., & Santana, C. M. (2015). A clinical comparison of guided bone regeneraBon with platelet-derived growth factor-enhanced bone ceramic versus autogenous bone block graFing. Int J Oral Maxillofac Implants, 30(3), 700-706. h5ps://doi.org/10.11607/jomi.3529
170. Schneider, D., Grunder, U., Ender, A., Hämmerle, C. H., & Jung, R. E. (2011). Volume gain and stability of peri-implant Bssue following bone and soF Bssue augmentaBon: 1-year results from a prospecBve cohort study. Clin Oral Implants Res, 22(1), 28-37. h5ps://doi.org/10.1111/j.1600-0501.2010.01987.x
171. Schneider, D., Weber, F. E., Grunder, U., Andreoni, C., Burkhardt, R., & Jung, R. E. (2014). A randomized controlled clinical mulBcenter trial comparing the clinical and histological performance of a new, modiﬁed polylacBde-co-glycolide acid membrane to an expanded polytetraﬂuorethylene membrane in guided bone regeneraBon procedures. Clin Oral Implants Res, 25(2), 150-158. h5ps://doi.org/10.1111/clr.12132
172. Schwarz, F., Hegewald, A., Sahm, N., & Becker, J. (2014). Long-term follow-up of simultaneous guided bone regeneraBon using naBve and cross-linked collagen membranes over 6 years. Clin Oral Implants Res, 25(9), 1010-1015. h5ps://doi.org/10.1111/clr.12220
173. Schwarz, F., Mihatovic, I., GhanaaB, S., & Becker, J. (2017). Performance and safety of collagenated xenogeneic bone block for lateral alveolar ridge augmentaBon and staged implant placement. A monocenter, prospecBve single-arm clinical study. Clin Oral Implants Res, 28(8), 954-960. h5ps://doi.org/10.1111/clr.12902
174. Schwarz, F., Obreja, K., Mayer, S., Ramanauskaite, A., Sader, R., & Parvini, P. (2022). Eﬃcacy of autogenous tooth roots for a combined verBcal and horizontal alveolar ridge augmentaBon and staged implant placement. A prospecBve controlled clinical study. J Clin Periodontol, 49(5), 496-505. h5ps://doi.org/10.1111/jcpe.13609
175. Schwarz, F., Sahm, N., & Becker, J. (2012). Impact of the outcome of guided bone regeneraBon in dehiscence-type defects on the long-term stability of peri-implant health: clinical observaBons at 4 years. Clin Oral Implants Res, 23(2), 191-196. h5ps://doi.org/10.1111/j.1600-0501.2011.02214.x
176. Schwarz, F., Schmucker, A., & Becker, J. (2017). Long-term outcomes of simultaneous guided bone regeneraBon using naBve and cross-linked collagen membranes aFer 8 years. Clin Oral Implants Res, 28(7), 779-784. h5ps://doi.org/10.1111/clr.12881
177. Sethi, A., & Kaus, T. (2000). Maxillary ridge expansion with simultaneous implant placement: 5-year results of an ongoing clinical study. Int J Oral Maxillofac Implants, 15(4), 491-499.
178. Simion, M., Misitano, U., Gionso, L., & Salvato, A. (1997). Treatment of dehiscences and fenestraBons around dental implants using resorbable and nonresorbable membranes associated with bone autograFs: a comparaBve clinical study. Int J Oral Maxillofac Implants, 12(2), 159-167.
179. Sjöström, M., Lundgren, S., Nilson, H., & Sennerby, L. (2005). Monitoring of implant stability in graFed bone using resonance frequency analysis. A clinical study from implant placement to 6 months of loading. Int J Oral Maxillofac Surg, 34(1), 45-51. h5ps://doi.org/10.1016/j.ijom.2004.03.007
180. Solakoglu, Ö., Götz, W., Heydecke, G., & Schwarzenbach, H. (2019). Histological and immunohistochemical comparison of two diﬀerent allogeneic bone graFing materials for alveolar ridge reconstrucBon: A prospecBve randomized trial in humans. Clin Implant Dent Relat Res, 21(5), 1002-1016. h5ps://doi.org/10.1111/cid.12824
181. Song, Y. W., Bienz, S. P., Benic, G. I., Cha, J. K., Hämmerle, C. H. F., Jung, U. W., & Jung, R. E. (2023). SoF-Bssue dimensional change following guided bone regeneraBon on peri-implant defects using soF-type block or parBculate bone subsBtutes: 1-year outcomes of a randomized controlled clinical trial. J Clin Periodontol, 50(2), 147-157. h5ps://doi.org/10.1111/jcpe.13738
182. Sterio, T. W., Katancik, J. A., Blanchard, S. B., Xenoudi, P., & Mealey, B. L. (2013). A prospecBve, mulBcenter study of bovine pericardium membrane with cancellous parBculate allograF for localized alveolar ridge augmentaBon. Int J PeriodonBcs RestoraBve Dent, 33(4), 499-507. h5ps://doi.org/10.11607/prd.1704
183. Strietzel, F. P., Reichart, P. A., & Graf, H. L. (2007). Lateral alveolar ridge augmentaBon using a syntheBc nano-crystalline hydroxyapaBte bone subsBtuBon material (OsBm): preliminary clinical and histological results. Clin Oral Implants Res, 18(6), 743-751. h5ps://doi.org/10.1111/j.1600-0501.2007.01416.x
184. Sugar, A. W., Thielens, P., Staﬀord, G. D., & Willins, M. J. (1995). AugmentaBon of the atrophic maxillary alveolar ridge with hydroxyapaBte granules in a Vicryl (polyglacBn 910) kni5ed tube and simultaneous open vesBbuloplasty. Br J Oral Maxillofac Surg, 33(2), 93-97. h5ps://doi.org/10.1016/0266-4356(95)90207-4
185. Temmerman, A., Cortellini, S., Van Dessel, J., De Greef, A., Jacobs, R., Dhondt, R., Teughels, W., & Quirynen, M. (2020). Bovine-derived xenograF in combinaBon with autogenous bone chips versus xenograF alone for the augmentaBon of bony dehiscences around oral implants: A randomized, controlled, split-mouth clinical trial. J Clin Periodontol, 47(1), 110-119. h5ps://doi.org/10.1111/jcpe.13209
186. Thoma, D. S., Payer, M., Jakse, N., Bienz, S. P., Hüsler, J., Schmidlin, P. R., Jung, U. W., Hämmerle, C. H. F., & Jung,

R. E. (2018). Randomized, controlled clinical two-centre study using xenogeneic block graFs loaded with recombinant human bone morphogeneBc protein-2 or autogenous bone blocks for lateral ridge augmentaBon. J Clin Periodontol, 45(2), 265-276. h5ps://doi.org/10.1111/jcpe.12841

187. Thor, A., Wannfors, K., Sennerby, L., & Rasmusson, L. (2005). ReconstrucBon of the severely resorbed maxilla with autogenous bone, platelet-rich plasma, and implants: 1-year results of a controlled prospecBve 5-year study. Clin Implant Dent Relat Res, 7(4), 209-220. h5ps://doi.org/10.1111/j.1708-8208.2005.tb00066.x
188. Thuaksuban, N., Nuntanaranont, T., & Pripatnanont, P. (2010). A comparison of autogenous bone graF combined with deproteinized bovine bone and autogenous bone graF alone for treatment of alveolar cleF. Int J Oral Maxillofac Surg, 39(12), 1175-1180. h5ps://doi.org/10.1016/j.ijom.2010.07.008
189. Torres, J., Tamimi, F., Alkhraisat, M. H., Manchón, A., Linares, R., Prados-Frutos, J. C., Hernández, G., & López Cabarcos, E. (2010). Platelet-rich plasma may prevent Btanium-mesh exposure in alveolar ridge augmentaBon with anorganic bovine bone. J Clin Periodontol, 37(10), 943-951. h5ps://doi.org/10.1111/j.1600-051X.2010.01615.x
190. Tresguerres, F. G. F., Tresguerres, I. F., Iglesias, O., Leco, I., Tamimi, F., & Torres, J. (2021). The role of corBcal perforaBons in allogeneic block graFing for lateral augmentaBon in maxilla: A randomized clinical trial. Clin Implant Dent Relat Res, 23(4), 530-542. h5ps://doi.org/10.1111/cid.12996
191. Tsai, Y. L., Tsao, J. P., Wang, C. L., Grodin, E., Lin, J. J., Chen, C. J., Ho, C. H., Cochran, D., & Mau, J. L. P. (2022).

Stability of contour augmentaBon of implant-supported single crowns in the estheBc zone: One-year cone-beam computed tomography results of a comparaBve, randomized, prospecBve, two-center clinical study using two diﬀerent bone graFing techniques in early implant placement. J Periodontol, 93(11), 1661-1670. h5ps://doi.org/10.1002/jper.21-0588

192. Van Assche, N., Michels, S., Naert, I., & Quirynen, M. (2013). Randomized controlled trial to compare two bone subsBtutes in the treatment of bony dehiscences. Clin Implant Dent Relat Res, 15(4), 558-568. h5ps://doi.org/10.1111/j.1708-8208.2011.00408.x
193. Van der Zee, E., Oosterveld, P., & Van Waas, M. A. (2004). Eﬀect of GBR and ﬁxture installaBon on gingiva and bone levels at adjacent teeth. Clin Oral Implants Res, 15(1), 62-65. h5ps://doi.org/10.1111/j.1600-0501.2004.01005.x
194. Veis, A. A., Tsirlis, A. T., & Parisis, N. A. (2004). Eﬀect of autogenous harvest site locaBon on the outcome of ridge augmentaBon for implant dehiscences. Int J PeriodonBcs RestoraBve Dent, 24(2), 155-163.
195. Velázquez Ó, I., Tresguerres, F. G. F., Berrocal, I. L., Tresguerres, I. F., López-Pintor, R. M., Carballido, J., López-Quiles, J., & Torres, J. (2021). Split bone block technique: 4-month results of a randomised clinical trial comparing clinical and radiographic outcomes between autogenous and xenogeneic corBcal plates. Int J Oral Implantol (Berl), 14(1), 41-52.
196. Waller, T., Herzog, M., Thoma, D. S., Hüsler, J., Hämmerle, C. H. F., & Jung, R. E. (2020). Long-term clinical and radiographic results aFer treatment or no treatment of small buccal bone dehiscences at posterior dental implants: A randomized, controlled clinical trial. Clin Oral Implants Res, 31(6), 517-525. h5ps://doi.org/10.1111/clr.13588
197. Wang, M., Li, Y., Su, Z., & Mo, A. (2023). Clinical and radiographic outcomes of customized allogeneic bone block versus autogenous bone block for ridge augmentaBon: 6 Month results of a randomized controlled clinical trial. J Clin Periodontol, 50(1), 22-35. h5ps://doi.org/10.1111/jcpe.13714
198. Wessing, B., Urban, I., Montero, E., Zechner, W., Hof, M., Alández Chamorro, J., Alández MarBn, N., Polizzi, G., Meloni, S., & Sanz, M. (2017). A mulBcenter randomized controlled clinical trial using a new resorbable non-cross-linked collagen membrane for guided bone regeneraBon at dehisced single implant sites: interim results of a bone augmentaBon procedure. Clin Oral Implants Res, 28(11), e218-e226. h5ps://doi.org/10.1111/clr.12995
199. Wu, Q., Yang, B., Gao, S., Gong, P., Xiang, L., Man, Y., & Qu, Y. (2019). Apical U-shape spliang technique for undercut areas of the anterior alveolar ridge: a prospecBve non-randomized controlled study. Int J Oral Maxillofac Surg, 48(3), 388-394. h5ps://doi.org/10.1016/j.ijom.2018.10.004
200. Xiao, W. L., Zhang, D. Z., Chen, X. J., Yuan, C., & Xue, L. F. (2016). Osteogenesis eﬀect of guided bone regeneraBon combined with alveolar cleF graFing: assessment by cone beam computed tomography. Int J Oral Maxillofac Surg, 45(6), 683-687. h5ps://doi.org/10.1016/j.ijom.2016.01.013
201. Xie, Y., Qin, Y., Wei, M., & Niu, W. (2024). ApplicaBon of sBcky bone combined with concentrated growth factor (CGF) for horizontal alveolar ridge augmentaBon of anterior teeth: a randomized controlled clinical study. BMC Oral Health, 24(1), 431. h5ps://doi.org/10.1186/s12903-024-04229-2
202. Zahedi, L., Mohammadi, M., Kalantari, M., Arabsolghar, M., & Ranjbar, H. (2024). Horizontal ridge augmentaBon with parBculate corBco-cancellous freeze-dried bone allograF alone or combined with injectable-platelet rich ﬁbrin in a randomized clinical trial. Clin Implant Dent Relat Res, 26(1), 127-137. h5ps://doi.org/10.1111/cid.13295
203. Zhao, X., Zou, L., Chen, Y., & Tang, Z. (2020). Staged horizontal bone augmentaBon for dental implants in aestheBc zones: A prospecBve randomized controlled clinical trial comparing a half-columnar bone block harvested from the ramus versus a rectangular bone block from the symphysis. Int J Oral Maxillofac Surg, 49(10), 1326-1334. h5ps://doi.org/10.1016/j.ijom.2019.12.010
204. Zitzmann, N. U., Naef, R., & Schärer, P. (1997). Resorbable versus nonresorbable membranes in combinaBon with Bio-Oss for guided bone regeneraBon. Int J Oral Maxillofac Implants, 12(6), 844-852.
205. Zuﬀea, F., Esposito, M., Capelli, M., Galli, F., Testori, T., & Del Fabbro, M. (2013). Socket graFing with or without buccal augmentaBon with anorganic bovine bone at immediate post-extracBve implants: 6-month aFer loading results from a mulBcenter randomised controlled clinical trial. Eur J Oral Implantol, 6(3), 239-250.
206. Abo-Elkheir, H. F., Moustafa, A. A. M., & Khalil, A. A. (2024). An Analogy between Two Bio-Membranes (CGF-PRGF) Mixed with Xenogeneic Bone GraF to Achieve Frugal Management of Labial Dehiscence amidst Immediate ImplantaBon in EstheBc Zone: A Randomized Clinical Trial. Open DenBstry Journal, 18. h5ps://doi.org/10.2174/0118742106295168240507162754
207. Annen, B. M., Ramel, C. F., Hämmerle, C. H., & Jung, R. E. (2011). Use of a new cross-linked collagen membrane for the treatment of peri-implant dehiscence defects: a randomised controlled double-blinded clinical trial. Eur J Oral Implantol, 4(2), 87-100.
208. Baghel, A., Sahoo, P., Sharma, J., Almutairi, F., Adhyaru, H., Chhatbar, R., & Bajoria, A. (2023). Assessment of biograF-HT with I-PRF graF in immediate dental implant placement. Journal of Pharmacy and Bioallied Sciences, 15(6), S1168-S1170. h5ps://doi.org/10.4103/jpbs.jpbs_178_23
209. Benic, G. I., Eisner, B. M., Jung, R. E., Basler, T., Schneider, D., & Hämmerle, C. H. F. (2019). Hard Bssue changes aFer guided bone regeneraBon of peri-implant defects comparing block versus parBculate bone subsBtutes: 6-month results of a randomized controlled clinical trial. Clinical Oral Implants Research, 30(10), 1016-1026. h5ps://doi.org/10.1111/clr.13515
210. Brägger, U., Hämmerle, C. H., & Lang, N. P. (1996). Immediate transmucosal implants using the principle of guided Bssue regeneraBon (II). A cross-secBonal study comparing the clinical outcome 1 year aFer immediate to standard implant placement. Clin Oral Implants Res, 7(3), 268-276. h5ps://doi.org/10.1034/j.1600-0501.1996.070309.x
211. Casse5a, M., Ricci, L., Iezzi, G., Dell'Aquila, D., Pia5elli, A., & Perroa, V. (2012). Resonance frequency analysis of implants inserted with a simultaneous graFing procedure: a 5-year follow-up study in man. InternaBonal journal of periodonBcs & restoraBve denBstry, 32(5), 581-589. h5ps://[www.cochranelibrary.com/central/doi/10.1002/central/CN-00839770/full](http://www.cochranelibrary.com/central/doi/10.1002/central/CN-00839770/full)
212. Chang, Y. C., Zhu, N., Liu, J., Gao, X., Chen, G., & Zhang, Y. (2025). EvaluaBng the Eﬀects of Dynamic NavigaBon on the Accuracy and Outcomes of the Autogenous Bone Ring Technique for VerBcal Ridge AugmentaBon: A Pilot Randomized Controlled Trial. Clinical Oral Implants Research. h5ps://doi.org/10.1111/clr.14412
213. Choudhary, D., Girdhar, G., Kumar, S., She5y, A., She5y, N., & Sinha, S. (2023). Flap versus ﬂapless immediate implants with bone augmentaBon: A novel study. Journal of Pharmacy and Bioallied Sciences, 15(6), S1082-S1085. h5ps://doi.org/10.4103/jpbs.jpbs_211_23
214. Cucchi, A., Beani, S., & Corinaldesi, G. (2022). A novel technique for digitalisaBon and customisaBon of reinforced polytetraﬂuoroethylene meshes: Preliminary results of a clinical trial. Int J Oral Implantol (Berl), 15(2), 129-146.
215. Cucchi, A., Beani, S., Fiorino, A., Maglio, M., Marchiori, G., Corinaldesi, G., & Sartori, M. (2024). Histological and histomorphometric analysis of bone Bssue using customized Btanium meshes with or without resorbable membranes: A randomized clinical trial. Clinical Oral Implants Research, 35(1), 114-130. h5ps://doi.org/10.1111/clr.14202
216. Cucchi, A., Beani, S., Tedeschi, L., Urban, I., Franceschi, D., Fiorino, A., & Corinaldesi, G. (2024). ComplicaBon, verBcal bone gain, volumetric changes aFer verBcal ridge augmentaBon using customized reinforced PTFE mesh or Ti-mesh. A non-inferiority randomized clinical trial. Clin Oral Implants Res, 35(12), 1616-1639. h5ps://doi.org/10.1111/clr.14350
217. D'Elia, C., Baldini, N., Cagidiaco, E. F., Nofri, G., Goracci, C., & de SancBs, M. (2017). Peri-implant SoF Tissue Stability AFer Single Implant RestoraBons Using Either Guided Bone RegeneraBon or a ConnecBve Tissue GraF: A Randomized Clinical Trial. The InternaBonal journal of periodonBcs & restoraBve denBstry, 37(3), 413-421. h5ps://doi.org/10.11607/prd.2747
218. De Bruyckere, T., Cabeza, R. G., Eghbali, A., Younes, F., Cleymaet, R., & Cosyn, J. (2020). A randomized controlled study comparing guided bone regeneraBon with connecBve Bssue graF to reestablish buccal convexity at implant sites: A 1-year volumetric analysis. Clin Implant Dent Relat Res, 22(4), 468-476. h5ps://doi.org/10.1111/cid.12934
219. El Zahwy, M., Taha, S., Mounir, R., & Mounir, M. (2019). Assessment of verBcal ridge augmentaBon and marginal bone loss using autogenous onlay vs inlay graFing techniques with simultaneous implant placement in the anterior maxillary estheBc zone: a randomized clinical trial. Clinical Implant DenBstry and Related Research, 21(6), 1140-1147. h5ps://doi.org/10.1111/cid.12849
220. Elbrashy, A., Osman, A. H., Shawky, M., Askar, N., & Atef, M. (2022). Immediate implant placement with platelet rich ﬁbrin as space ﬁlling material versus deproteinized bovine bone in maxillary premolars: A randomized clinical trial. Clinical Implant DenBstry and Related Research, 24(3), 320-328. h5ps://doi.org/10.1111/cid.13075
221. Felice, P., PisBlli, R., Pellegrino, G., Bonifazi, L., Tayeb, S., Simion, M., & Barausse, C. (2024). A randomised controlled trial comparing the eﬀecBveness of guided bone regeneraBon with polytetraﬂuoroethylene Btanium-reinforced membranes, CAD/CAM semi-occlusive Btanium meshes and CAD/CAM occlusive Btanium foils in parBally atrophic arches. Int J Oral Implantol (Berl), 17(3), 285-296.
222. Fe5ouh, A. I. A., Ghallab, N. A., Ghaﬀar, K. A., Mina, N. A., Abdelmalak, M. S., Abdelrahman, A. A. G., & Shemais, N. M. (2023). Bone dimensional changes aFer ﬂapless immediate implant placement with and

without bone graFing: Randomized clinical trial. Clinical Implant DenBstry and Related Research, 25(2), 271-

283. h5ps://doi.org/10.1111/cid.13178

223. Garcia-Sanchez, R., Mardas, N., BuB, J., OrBz Ruiz, A. J., & Pardo Zamora, G. (2021). Immediate implant placement in fresh alveolar sockets with a minimal split-thickness envelope ﬂap: A randomised controlled clinical trial. Clin Oral Implants Res, 32(9), 1115-1126. h5ps://doi.org/10.1111/clr.13806
224. Ghanem, M., Heikal, L., Abdel Fa5ah, H., El Ashwah, A., & Fliefel, R. (2022). The Eﬀect of Coenzyme Q10/Collagen Hydrogel on Bone RegeneraBon in ExtracBon Socket Prior to Implant Placement in Type II DiabeBc PaBents: A Randomized Controlled Clinical Trial. Journal of Clinical Medicine, 11(11). h5ps://doi.org/10.3390/jcm11113059
225. Girlanda, F. F., Feng, H. S., Corrêa, M. G., CasaB, M. Z., Pimentel, S. P., Ribeiro, F. V., & Cirano, F. R. (2019). Deproteinized bovine bone derived with collagen improves soF and bone Bssue outcomes in ﬂapless immediate implant approach and immediate provisionalizaBon: a randomized clinical trial. Clin Oral InvesBg, 23(10), 3885-3893. h5ps://doi.org/10.1007/s00784-019-02819-x
226. Gurbuz, E., & Ceylan, E. (2025). Comparison of a non-graFed socket shield technique with guided bone regeneraBon in immediate implant placement: a randomized clinical trial. Int J Oral Maxillofac Surg, 54(4), 356-

364. h5ps://doi.org/10.1016/j.ijom.2024.11.009

227. Hamed, M. M., El-Tonsy, M. M., Elaskary, A., Abdelaziz, G. O., Saeed, S. S., & Elfahl, B. N. (2023). Eﬀect of three diﬀerent graFing materials on immediate implant placement using vesBbular socket therapy in class II extracBon sockets in the maxillary estheBc zone: a randomized controlled clinical trial. BMC Oral Health, 23(1), 623. h5ps://doi.org/10.1186/s12903-023-03345-9
228. Hamzah, B., Mounir, R., Ali, S., & Mounir, M. (2021). Maxillary horizontal alveolar ridge augmentaBon using computer guided ridge spliang with simultaneous implant placement versus convenBonal technique: A randomized clinical trial. Clinical Implant DenBstry and Related Research, 23(4), 555-561. h5ps://doi.org/10.1111/cid.13015
229. Hartmann, A., Welte-Jzyk, C., Seiler, M., & Daublander, M. (2017). Neurophysiological changes associated with implant-associated augmentaBon procedures in the lower jaw. Clinical Implant DenBstry and Related Research, 19(4), 725-732. h5ps://doi.org/10.1111/cid.12500
230. Hsu, A., Seong, W. J., Wolﬀ, R., Zhang, L., Hodges, J., Olin, P. S., & Hinrichs, J. E. (2016). Comparison of IniBal Implant Stability of Implants Placed Using BicorBcal FixaBon, Indirect Sinus ElevaBon, and UnicorBcal FixaBon. The InternaBonal journal of oral & maxillofacial implants, 31(2), 459-468. h5ps://doi.org/10.11607/jomi.4142
231. Hussein, L. A., & Hassan, T. A. L. (2017). The eﬀecBveness of oxidized regenerated cellulose AS a graF material in transalveolar osteotome sinus liF procedure. Journal of Craniofacial Surgery, 28(7), 1766-1771. h5ps://doi.org/10.1097/SCS.0000000000003943
232. Iglesias-Velázquez, Ó., Zamora, R. S., López-Pintor, R. M., Tresguerres, F. G. F., Berrocal, I. L., García, C. M., Tresguerres, I. F., & García-Denche, J. T. (2022). Periosteal Pocket Flap technique for lateral ridge augmentaBon. A comparaBve pilot study versus guide bone regeneraBon. Anatomischer Anzeiger [Annals of anatomy], 243, 151950. h5ps://doi.org/10.1016/j.aanat.2022.151950
233. Jabbari, N., Shooshtari, Z., Mohammadi, S., Ghazi, N., & Kazemian, M. (2024). Does Collagen Membrane Coverage Oﬀer any Advantage for Lateral Window Sinus AugmentaBon? A Histologic and Histomorphometric Analysis. World journal of plasBc surgery, 13(3), 49-56. h5ps://doi.org/10.61186/wjps.13.3.49
234. Jain, D., Mohan, R., & Singh, V. D. (2019). Comparison of microsurgical and macrosurgical technique using bioacBve syntheBc bone graF and collagen membrane for an implant site development: A randomized controlled clinical trial. Journal of Indian Society of Periodontology, 23(5), 448-460. h5ps://doi.org/10.4103/jisp.jisp_738_18
235. Jiang, X., Zhang, Y., Di, P., & Lin, Y. (2018). Hard Bssue volume stability of guided bone regeneraBon during the healing stage in the anterior maxilla: A clinical and radiographic study. Clinical Implant DenBstry and Related Research, 20(1), 68-75. h5ps://doi.org/10.1111/cid.12570
236. Jing, W., Yilin, L., Dingming, H., Huangpha5arakul, V., & Yi, M. (2023). Implant Placement with Simultaneous Guided Bone RegeneraBon in the Anterior Region Close to the Periapical Lesion of Adjacent Teeth: a Combined Treatment Strategy to Prevent ComplicaBons. InternaBonal journal of oral & maxillofacial implants, 38(3), 533-

546. h5ps://doi.org/10.11607/jomi.9839

237. Jung, R. E., Fenner, N., Hammerle, C. H., & Zitzmann, N. U. (2013). Long-term outcome of implants placed with guided bone regeneraBon (GBR) using resorbable and non-resorbable membranes aFer 12-14 years. Clinical Oral Implants Research, 24(10), 1065-1073. h5ps://doi.org/10.1111/j.1600-0501.2012.02522.x
238. Kaigler, D., Avila-OrBz, G., Travan, S., Taut, A. D., Padial-Molina, M., Rudek, I., Wang, F., Lanis, A., & Giannobile,

W. V. (2015). Bone Engineering of Maxillary Sinus Bone Deﬁciencies Using Enriched CD90+ Stem Cell Therapy: A Randomized Clinical Trial. Journal of Bone and Mineral Research, 30(7), 1206-1216. h5ps://doi.org/10.1002/jbmr.2464

239. Kauﬀmann, F., Fickl, S., Sculean, A., Fischer, K. R., & Friedmann, A. (2023). Alveolar ridge alteraBons aFer lateral guided bone regeneraBon with and without hyaluronic acid: a prospecBve randomized trial with morphometric and histomorphometric evaluaBon. Quintessence Int, 54(9), 712-722. h5ps://doi.org/10.3290/j.qi.b4171703
240. Khalifah, M. A., Elgendy, A. M. A., & Elgendy, E. (2024). Untreated Mineralized DenBn GraFs (UMDGs) vs XenograFs Around Immediately Placed Dental Implants in the Mandibular Anterior Region: A Randomized Controlled Clinical Trial. Int J Oral Maxillofac Implants(3), 381-388. h5ps://doi.org/10.11607/jomi.10584
241. Khorshed, K. A. A. E., Elgamal, M., Haggag, M. A., & Elsyad, M. A. (2024). Closed sinus liF and ﬁxed prostheses versus implant-assisted overdentures in management of atrophied distal extension maxillary ridges: A one-year randomized clinical trial. The Journal of ProstheBc DenBstry. h5ps://doi.org/10.1016/j.prosdent.2024.01.035
242. Kim, Y. K., Kim, S. G., Lim, S. C., Lee, H. J., & Yun, P. Y. (2010). A clinical study on bone formaBon using a demineralized bone matrix and resorbable membrane. Oral Surg Oral Med Oral Pathol Oral Radiol Endod, 109(6), e6-11. h5ps://doi.org/10.1016/j.tripleo.2010.01.012
243. Cordaro, L., Torsello, F., Accorsi Ribeiro, C., Liberatore, M., & Mirisola di Torresanto, V. (2010). Inlay-onlay graFing for three-dimensional reconstrucBon of the posterior atrophic maxilla with mandibular bone. *Int J Oral Maxillofac Surg*, 39(4), 350–357. h5ps://doi.org/10.1016/j.ijom.2010.02.016
244. Laass, A., Eisner, B. M., Hammerle, C. H. F., Jung, R. E., Thoma, D. S., & Benic, G. I. (2020). Histologic outcomes aFer guided bone regeneraBon of peri-implant defects comparing individually shaped block versus parBculate bone subsBtutes. InternaBonal journal of periodonBcs & restoraBve denBstry, 40(4), 519-527. h5ps://doi.org/10.11607/prd.4575
245. Laino, L., Iezzi, G., Pia5elli, A., Lo Muzio, L., & Cicciù, M. (2014). VerBcal ridge augmentaBon of the atrophic posterior mandible with sandwich technique: Bone block from the chin area versus corBcocancellous bone block allograF - Clinical and histological prospecBve randomized controlled study. BioMed Research InternaBonal, 2014. h5ps://doi.org/10.1155/2014/982104
246. Li, X., Jin, Q., Xu, H., Zhang, S., Wang, W., & Zhao, B. (2023). Eﬀect of PolylacBc Acid Membrane on Guided Bone RegeneraBon in Anterior Maxillary ImplantaBon. Medical Science Monitor, 29, e938566. h5ps://doi.org/10.12659/MSM.938566
247. Li, Y., Qiao, S. C., Gu, Y. X., Zhang, X. M., Shi, J. Y., & Lai, H. C. (2019). A novel semiautomaBc segmentaBon protocol to evaluate guided bone regeneraBon outcomes: A pilot randomized, controlled clinical trial. Clin Oral Implants Res, 30(4), 344-352. h5ps://doi.org/10.1111/clr.13420
248. Majzoub, Z., Cordioli, G., Aramouni, P. K., Vigolo, P., & Pia5elli, A. (1999). Guided bone regeneraBon using demineralized laminar bone sheets versus GTAM membranes in the treatment of implant-associated defects. A clinical and histological study. Clin Oral Implants Res, 10(5), 406-414. h5ps://doi.org/10.1034/j.1600-0501.1999.100507.x
249. Malmström, J., Slo5e, C., Adolfsson, E., Norderyd, O., & Thomsen, P. (2009). Bone response to free form-fabricated hydroxyapaBte and zirconia scaﬀolds: a histological study in the human maxilla. Clin Oral Implants Res, 20(4), 379-385. h5ps://doi.org/10.1111/j.1600-0501.2008.01595.x
250. Marx, R. E., Armentano, L., Olavarria, A., & Samaniego, J. (2013). rhBMP-2/ACS graFs versus autogenous cancellous marrow graFs in large verBcal defects of the maxilla: an unsponsored randomized open-label clinical trial. Int J Oral Maxillofac Implants, 28(5), e243-251. h5ps://doi.org/10.11607/jomi.te04
251. Ma5out, P., Maddalozzo, D., Robinson, P. J., Hramiec, S. R., McLey, L., & Zahradnik, R. (1994). Clinical evaluaBon of guided bone regeneraBon at exposed parts of Btanium dental implants with and without bone allograFs. Journal of Dental Research, 65(Special Issue IADR Abstracts - Sea5le March 10-13 1994). h5ps://[www.cochranelibrary.com/central/doi/10.1002/central/CN-00229170/full](http://www.cochranelibrary.com/central/doi/10.1002/central/CN-00229170/full)
252. MeshkatAlsadat, M., Hassani, A., Bitaraf, T., & Salmasi, S. C. (2022). Dimensional changes of peri-implant Bssue following immediate ﬂapless implant placement and provisionalizaBon with or without xenograF in the anterior maxilla: a study protocol for a randomized controlled trial. Trials, 23(1). h5ps://doi.org/10.1186/s13063-022-06918-1
253. Naenni, N., Schneider, D., Jung, R. E., Hüsler, J., Hämmerle, C. H. F., & Thoma, D. S. (2017). Randomized clinical study assessing two membranes for guided bone regeneraBon of peri-implant bone defects: clinical and histological outcomes at 6 months. Clin Oral Implants Res, 28(10), 1309-1317. h5ps://doi.org/10.1111/clr.12977
254. Nahid, R., Bansal, M., & Pandey, S. (2022). Horizontal bone augmentaBon using two membranes at dehisced implant sites: a randomized clinical study. Journal of oral biology and craniofacial research, 12(5), 487-491. h5ps://doi.org/10.1016/j.jobcr.2022.06.003
255. Nasrabadi, N., Forouzanfar, A., Farsi, M. R., Babaei, K., & Ahrari, F. (2023). Eﬀect of Bone GraFing on Hard and SoF Tissue Changes Following Immediate Implant InstallaBon in Mandibular Molar Sites: A Double-Blind Randomized Clinical Trial. J Long Term Eﬀ Med Implants, 33(3), 87-96. h5ps://doi.org/10.1615/JLongTermEﬀMedImplants.2022044833
256. Ovcharenko, N., Greenwell, H., Katwal, D., Patel, A., Hill, M., Shumway, B., & Cockerham, B. L., 3rd. (2020). A Comparison of the Eﬀect of Barrier Membranes on Clinical and Histologic Hard and SoF Tissue Healing with Ridge PreservaBon. Int J PeriodonBcs RestoraBve Dent, 40(3), 365-371. h5ps://doi.org/10.11607/prd.4120
257. Pappalardo, S., Baglio, O. A., Frasca, M., & Grassi, F. R. (2004). Alveolar ridge augmentaBon by means of onlay graFs harvested from mandibular symphysis. Minerva Stomatologica, 53(4), 143-150. h5ps://[www.embase.com/search/results?subacBon=viewrecord&id=L38909699&from=export](http://www.embase.com/search/results?subacBon=viewrecord&id=L38909699&from=export)
258. Park, S., Lee, J. Y., Park, J. Y., Song, Y. W., Cha, J. K., Strauss, F. J., Shin, S. Y., Lim, H. C., Jung, R. E., Jung, U. W., & Thoma, D. S. (2025). Ridge AugmentaBon Using a Self-Retaining Block Bone Material in Damaged ExtracBon Sockets: A MulB-Centre Randomized Controlled Clinical Trial. Journal of Clinical Periodontology. h5ps://doi.org/10.1111/jcpe.14135
259. Payer, M., Tan, W. C., Han, J., Ivanovski, S., Ma5heos, N., Pjetursson, B. E., Zhuang, L., Fokas, G., Wong, M. C. M., Acham, S., & et al. (2020). The eﬀect of systemic anBbioBcs on clinical and paBent-reported outcome measures of oral implant therapy with simultaneous guided bone regeneraBon. Clinical Oral Implants Research, 31(5), 442-451. h5ps://doi.org/10.1111/clr.13580
260. PisBlli, R., Felice, P., Pia5elli, M., Gessaroli, M., Soardi, E., Barausse, C., BuB, J., & Corvino, V. (2013). Posterior atrophic jaws rehabilitated with prostheses supported by 5 x 5 mm implants with a novel nanostructured calcium-incorporated Btanium surface or by longer implants in augmented bone. One-year results from a randomised controlled trial. Eur J Oral Implantol, 6(4), 343-357.
261. Pongse5akul, A., Leepong, N., & Su5apreyasri, S. (2024). Repairing Dehiscence Defects at Implant Sites using β-Tricalcium Phosphate/Calcium Sulfate versus XenograF Combined With Membrane: A Randomized Clinical Trial. The Journal of oral implantology. h5ps://doi.org/10.1563/aaid-joi-D-24-00048
262. Puisys, A., Auzbikaviciute, V., Vindasiute-Narbute, E., Pranskunas, M., Razukevicus, D., & Linkevicius, T. (2022). Immediate implant placement vs. early implant treatment in the estheBc area. A 1-year randomized clinical trial. Clin Oral Implants Res, 33(6), 634-655. h5ps://doi.org/10.1111/clr.13924
263. Raghoebar, G. M., Liem, R. S., Bos, R. R., Van Der Wal, J. E., & Vissink, A. (2006). Resorbable screws for ﬁxaBon of autologous bone graFs. Clinical Oral Implants Research, 17(3), 288-293. h5ps://doi.org/10.1111/j.1600-0501.2005.01200.x
264. Rajanna, H., Chaudhry, K., Pandey, A. K., Somvanshi, S., Singh, S., Kumar, P., & Chugh, A. (2024). Eﬃcacy of Oral Magnesium on Bone RegeneraBon and Implant Stability in Immediate Dental Implants: A Randomized Controlled Trial. The InternaBonal journal of oral & maxillofacial implants, 1-31. h5ps://doi.org/10.11607/jomi.11017
265. Santana, R. B., Santana, C. M., & Dibart, S. (2015). Platelet-Derived Growth Factor-Mediated Guided Bone RegeneraBon in Immediate Implant Placement in Molar Sites with Buccal Bone Defects. InternaBonal journal of periodonBcs & restoraBve denBstry, 35(6), 825-833. h5ps://doi.org/10.11607/prd.2330
266. Shibly, O., Patel, N., Albandar, J. M., & Kutkut, A. (2010). Bone regeneraBon around implants in periodontally compromised paBents: a randomized clinical trial of the eﬀect of immediate implant with immediate loading. J Periodontol, 81(12), 1743-1751. h5ps://doi.org/10.1902/jop.2010.100162
267. Shido, R., Ohba, S., Tominaga, R., Sumita, Y., & Asahina, I. (2023). A ProspecBve Study of the Assessment of the Eﬃcacy of a Biodegradable Poly(l-lacBc acid/ε-caprolactone) Membrane for Guided Bone RegeneraBon. Journal of Clinical Medicine, 12(18). h5ps://doi.org/10.3390/jcm12185994
268. Shiezadeh, F., Arab, H. R., Khoshkam, V., Moeintaghavi, A., Forouzanfar, A., & Khodadadifard, L. (2023). Comparison of Guided Bone RegeneraBon with Periosteal Pocket Flap Technique Versus Autogenous Bone Block GraF for Horizontal Bone AugmentaBon: A Clinical Trial Study. InternaBonal Journal of PeriodonBcs and RestoraBve DenBstry, 43(4), 479-488. h5ps://doi.org/10.11607/prd.5691
269. Shomurodov, K. E., & Mirkhusanova, R. S. K. (2023). APPLICATION OF THE SOFT TISSUE EXPANDING TECHNIQUE

BEFORE GUIDED BONE REGENERATION OF AN ATROPHIC ALVEOLAR RIDGE. Journal of health and translaBonal medicine, 26(1), 170-178. h5ps://doi.org/10.22452/jummec.vol26no1.23

270. Sinha, S., Kumar, S., Sonoo, P., Kumar, U., Siddhartha, R., & Singh, S. (2021). EvaluaBon of bone regeneraBon around implants with and without ﬂap elevaBon. Journal of Pharmacy and Bioallied Sciences, 13(5), S699-S705. h5ps://doi.org/10.4103/jpbs.JPBS_691_20
271. Spin-Neto, R., Stavropoulos, A., Colea, F. L., Faeda, R. S., Pereira, L. A., & Marcantonio, E. (2014). GraF incorporaBon and implant osseointegraBon following the use of autologous and fresh-frozen allogeneic block bone graFs for lateral ridge augmentaBon. Clinical Oral Implants Research, 25(2), 226-233. h5ps://doi.org/10.1111/clr.12107
272. Thoma, D. S., Bienz, S. P., Lim, H. C., Lee, W. Z., Hämmerle, C. H. F., & Jung, R. E. (2020). ExploraBve randomized controlled study comparing soF Bssue thickness, contour changes, and soF Bssue handling of two ridge preservaBon techniques and spontaneous healing two months aFer tooth extracBon. Clinical Oral Implants Research, 31(6), 565-574. h5ps://doi.org/10.1111/clr.13594
273. Urban, I. A., Serroni, M., Dias, D. R., Baráth, Z., Forster, A., Araújo, T. G., Saleh, M. H. A., Cucchi, A., & Ravidà, A. (2025). Impact of Collagen Membrane in VerBcal Ridge AugmentaBon Using Ti-Reinforced PTFE Mesh: A Randomised Controlled Trial. Journal of Clinical Periodontology. h5ps://doi.org/10.1111/jcpe.14129
274. Urban, I. A., Wessing, B., Alández, N., Meloni, S., González-MarBn, O., Polizzi, G., Sanz-Sanchez, I., Montero, E., & Zechner, W. (2019). A mulBcenter randomized controlled trial using a novel collagen membrane for guided bone regeneraBon at dehisced single implant sites: Outcome at prostheBc delivery and at 1-year follow-up. Clin Oral Implants Res, 30(6), 487-497. h5ps://doi.org/10.1111/clr.13426
275. Wang, J., Chen, W., Huang, M., Zhong, Z., Wang, P., & Wu, R. (2024). ComparaBve Analysis of GelaBn/PolylacBc Acid and Commercial PLA Membranes for Guided Bone RegeneraBon: A Randomized Clinical Trial. Medical Science Monitor, 30. h5ps://doi.org/10.12659/MSM.944713
276. Wang, Y., Fu, G., Zhang, J., Xu, Y., Shen, M., Yi, Z., Lan, J., Li, Q., Zhao, Y., Wu, R., & et al. (2025). Bioceramics for Guided Bone RegeneraBon: a MulBcenter Randomized Controlled Trial. Clinical Implant DenBstry and Related Research, 27(1), e13437. h5ps://doi.org/10.1111/cid.13437
277. Watzinger, F., Luksch, J., Millesi, W., Schopper, C., Neugebauer, J., Moser, D., & Ewers, R. (2000). Guided bone regeneraBon with Btanium membranes: a clinical study. Br J Oral Maxillofac Surg, 38(4), 312-315. h5ps://doi.org/10.1054/bjom.1999.0228
278. Wessels, R., Vervaeke, S., Seyssens, L., Eghbali, A., & Cosyn, J. (2020). A 5-year cohort study on early implant placement with guided bone regeneraBon or alveolar ridge preservaBon with connecBve Bssue graF. Clinical Implant DenBstry and Related Research, 22(6), 697-705. h5ps://doi.org/10.1111/cid.12948
279. Yagihara, K., Okabe, S., Ishii, J., Amagasa, T., Yamashiro, M., Yamaguchi, S., Yokoya, S., Yamazaki, T., & Kinoshita,

Y. (2013). Mandibular reconstrucBon using a poly(L-lacBde) mesh combined with autogenous parBculate cancellous bone and marrow: a prospecBve clinical study. Int J Oral Maxillofac Surg, 42(8), 962-969. h5ps://doi.org/10.1016/j.ijom.2013.03.010

280. Zazou, N., Diab, N., Bahaa, S., El Arab, A. E., Aziz, O. A., & El Nahass, H. (2021). Clinical comparison of diﬀerent ﬂap advancement techniques to periosteal releasing incision in guided bone regeneraBon: A randomized controlled trial. Clin Implant Dent Relat Res, 23(1), 107-116. h5ps://doi.org/10.1111/cid.12960
281. Zhu, L., Du, X., Fu, G., Wang, L., Huang, H., Wu, X., & Xu, B. (2025). Eﬃcacy of diﬀerent forms of concentrated growth factors combined with deproteinized bovine bone minerals in guided bone regeneraBon: a randomized clinical trial. BMC Oral Health, 25(1), 320. h5ps://doi.org/10.1186/s12903-025-05698-9
282. Zuercher, A. N., Mühlemann, S., Ruales-Carrera, E., Hjerppe, J., Jung, R. E., & Thoma, D. S. (2024). Comparing Small Buccal Dehiscence Defects Treated with or without Guided Bone RegeneraBon. A Sub-Analysis of a RCT. The InternaBonal journal of periodonBcs & restoraBve denBstry, 1-17. h5ps://doi.org/10.11607/prd.7138
283. Zuercher, A. N., Paque, N., Hämmerle, C., Jung, R. E., & Thoma, D. S. (2022). Randomized controlled pilot study comparing small buccal dehiscence defects around dental implants treated with autogenous connecBve Bssue graF (SCTG) or standard guided bone regeneraBon (GBR). Journal of Clinical Periodontology, 49, 139-140. h5ps://doi.org/10.1111/jcpe.13635
284. Raes, F., Cosyn, J., & De Bruyn, H. (2013). Clinical, aestheBc, and paBent-related outcome of immediately loaded single implants in the anterior maxilla: a prospecBve study in extracBon sockets, healed ridges, and graFed sites. Clinical Implant DenBstry and Related Research, 15(6), 819-835. h5ps://doi.org/10.1111/j.1708-8208.2011.00438.x
285. Sivolella, S., Boacelli, D., Prasad, S., Ricci, S., Bressan, E., & Prasad, H. (2020). EvaluaBon and comparison of histologic changes and implant survival in extracBon sites immediately graFed with two diﬀerent xenograFs: a randomized clinical pilot study. Clinical Oral Implants Research, 31(9), 825-835. h5ps://doi.org/10.1111/clr.13626
286. Cucchi, A., Vignudelli, E., Lunardi, L., Bortolini, S., Bianchi, A., GiannaBempo, J., & Corinaldesi, G. (2019). Guided bone regeneraBon with customized meshes with and without collagen membrane. 98(Spec Iss A). h5ps://[www.cochranelibrary.com/central/doi/10.1002/central/CN-02361960/full](http://www.cochranelibrary.com/central/doi/10.1002/central/CN-02361960/full)
287. Assessment of clinical and radiographic outcomes of guided bone regeneraBon with dehydrothermally cross-linked collagen membrane around peri-implant dehiscence defects: results from a 3-year randomized clinical trial. (2019). 43(1), 8-16. h5ps://doi.org/10.21851/obr.43.01.201903.8
288. Chiapasco, M., Romeo, E., AbaB, S., & Vogel, G. (1997). A comparaBve study between guided bone regeneraBon and autogenous bone blocks for the reconstrucBon of narrow edentulous ridges. 24(11), 870. h5ps://[www.cochranelibrary.com/central/doi/10.1002/central/CN-00763320/full](http://www.cochranelibrary.com/central/doi/10.1002/central/CN-00763320/full)
289. Janyaphadungpong, R., Serichetaphongse, P., & Pimkhaokham, A. (2019). A clinical resonance frequency analysis of implants placed at dehiscence-type defects with simultaneous guided bone regeneraBon during early healing. 34(3), 772–777. h5ps://doi.org/10.11607/jomi.6834
290. Zitzmann, N. U., Scharer, P., & Marinello, C. P. (2001). Long-term results of implants treated with guided bone regeneraBon: a 5-year prospecBve study. 16(3). h5ps://[www.cochranelibrary.com/central/doi/10.1002/central/CN-00387535/full](http://www.cochranelibrary.com/central/doi/10.1002/central/CN-00387535/full)
291. Eskan, M. A., Greenwell, H., Hill, M., Morton, D., Vidal, R., Shumway, B., & Girouard, M. E. (2014). Platelet-rich plasma-assisted guided bone regeneraBon for ridge augmentaBon: a randomized, controlled clinical trial. 85(5), 661-668. h5ps://doi.org/10.1902/jop.2013.130260
292. Nowzari, H., & Slots, J. (1995). Microbiologic and clinical study of polytetraﬂuoroethylene membranes for guided bone regeneraBon around implants. 10(1). h5ps://[www.cochranelibrary.com/central/doi/10.1002/central/CN-00228676/full](http://www.cochranelibrary.com/central/doi/10.1002/central/CN-00228676/full)
293. Tony, J. B., Parthasarathy, H., Tadepalli, A., Ponnaiyan, D., Alamoudi, A., Kamil, M. A., Alzahrani, K. J., Alsharif, K. F., Halawani, I. F., Alnﬁai, M. M., & et al. (2022). CBCT EvaluaBon of SBcky Bone in Horizontal Ridge AugmentaBon with and without Collagen Membrane-A Randomized Parallel Arm Clinical Trial. 13(4). h5ps://doi.org/10.3390/jö13040194
294. Luongo, R., Tallarico, M., Canciani, E., Graziano, D., Dellavia, C., Gargari, M., Ceruso, F. M., Melodia, D., & Canullo, L. (2022). Histomorphometry of Bone aFer IntenBonally Exposed Non-Resorbable d-PTFE Membrane or Guided Bone RegeneraBon for the Treatment of Post-ExtracBve Alveolar Bone Defects with Implant-Supported RestoraBons: a Pilot Randomized Controlled Trial. 15(17). h5ps://doi.org/10.3390/ma15175838
295. Jung, R. E., Glauser, R., Schärer, P., Hämmerle, C. H., Sailer, H. F., & Weber, F. E. (2003). Eﬀect of rhBMP-2 on guided bone regeneraBon in humans. 14(5), 556-568. h5ps://doi.org/10.1034/j.1600-0501.2003.00921.x
296. Dhondt, R. A. L., Quirynen, M., Lahoud, P., Cortellini, S., & Temmerman, A. (2025). Horizontal Guided Bone RegeneraBon: L-PRF Bone-Block Vs A Mixture of Autogenous Bone with Deproteinized Bovine Bone Mineral. A Split-Mouth RCT Study with 25 Months Follow-Up. 0(0), 1-30. h5ps://doi.org/10.11607/jomi.11095
297. Nagaraj, A., She5y, M., Thomas, B., & Hegde, R. (2024). RIDGE AUGMENTATION USING AUTOGRAFT AND XENOGRAFT VERSUS XENOGRAFT ALONE WITH SIMULTANEOUS IMPLANT PLACEMENT: a RANDOMISED CLINICAL TRIAL. 51, 83-89. h5ps://doi.org/10.2478/AMB-2024-0057
298. Wang, J., Chen, W., Huang, M., Zhong, Z., Wang, P., & Wu, R. (2025). ComparaBve Analysis of GelaBn/PolylacBc Acid and Commercial PLA Membranes for Guided Bone RegeneraBon: a Randomized Clinical Trial. 31, e944713. h5ps://doi.org/10.12659/MSM.944713
299. Ashurko, I., Samsonov, A., Galyas, A., Petukhova, M., Tarasenko, S., & Unkovskiy, A. (2024). RehabilitaBon Using Implants with Sloped Plaõorm Edge vs. Standard Plaõorm with Guided Bone RegeneraBon: a Randomized Control Clinical Trial. 12(7). h5ps://doi.org/10.3390/dj12070205
300. De Angelis, P., CavalcanB, C., Manicone, P. F., Liguori, M. G., Rella, E., De Rosa, G., Palmieri, A., & D'Addona, A. (2024). A Comparison of Guided Bone RegeneraBon vs. the Shell Technique Using Xenogeneic Bone Blocks in Horizontal Bone Defects: a Randomized Clinical Trial. 12(5). h5ps://doi.org/10.3390/dj12050137
301. Tsai, Y. L., Tsao, J. P., Wang, C. L., Grodin, E., Lin, J. J., Chen, C. J., Ho, C. H., Cochran, D., & Mau, J. L. P. (2022).

Stability of contour augmentaBon of implant-supported single crowns in the estheBc zone: one-year cone-beam computed tomography results of a comparaBve, randomized, prospecBve, two-center clinical study using two diﬀerent bone graFing techniques in. 93(11), 1661-1670. h5ps://doi.org/10.1002/JPER.21-0588

302. Simion, M., Jovanovic, S. A., Trisi, P., Scarano, A., & Pia5elli, A. (1998). VerBcal ridge augmentaBon around dental implants using a membrane technique and autogenous bone or allograFs in humans. 18(1), 8-23. h5ps://[www.cochranelibrary.com/central/doi/10.1002/central/CN-00149889/full](http://www.cochranelibrary.com/central/doi/10.1002/central/CN-00149889/full)
303. Schwarz, F., Hazar, D., Becker, K., Sader, R., & Becker, J. (2018). Eﬃcacy of autogenous tooth roots for lateral alveolar ridge augmentaBon and staged implant placement. A prospecBve controlled clinical study. 45(8), 996-1004. h5ps://doi.org/10.1111/jcpe.12977
304. Zumstein, T., & Sennerby, L. (2016). A 1-year clinical and radiographic study on hydrophilic dental implants placed with and without bone augmentaBon procedures. 18(3), 498-506. h5ps://doi.org/10.1111/cid.12329
305. Merli, M., Marioa, G., Pagliaro, U., Mazzoni, A., Moscatelli, M., & Nieri, M. (2020). The Fence Technique: 100% Autogenous Bone GraF vs 50% Deproteinized Bovine Bone Matrix and 50% Autogenous Bone GraF. A Histologic Randomized Controlled Trial. 40(2), 181-190. h5ps://doi.org/10.11607/prd.4116
306. Felice, P., Marchea, C., Iezzi, G., Pia5elli, A., Worthington, H., Pellegrino, G., & Esposito, M. (2009). VerBcal ridge augmentaBon of the atrophic posterior mandible with interposiBonal bloc graFs: bone from the iliac crest vs. bovine anorganic bone. Clinical and histological results up to one year aFer loading from a randomized-controlled clinical tri. 20(12), 1386-1393. h5ps://doi.org/10.1111/j.1600-0501.2009.01765.x
307. Asahina, I., Kagami, H., Agata, H., Honda, M. J., Sumita, Y., Inoue, M., Nagamura-Inoue, T., & Tojo, A. (2021). Clinical outcome and 8-year follow-up of alveolar bone Bssue engineering for severely atrophic alveolar bone using autologous bone marrow stromal cells with platelet-rich plasma and β-tricalcium phosphate granules. Journal of Clinical Medicine, 10(22). h5ps://doi.org/10.3390/jcm10225231
308. Bawankar, P., Kolte, A., & Kolte, R. (2023). PaBent-centered outcome measures comparing the autogenous and allogenic bone blocks in the augmentaBon of deﬁcient alveolar ridges: A pilot study. Journal of Indian Society of Periodontology, 27(1), 87-94. h5ps://doi.org/10.4103/jisp.jisp_733_21
309. Benic, G. I., Ge, Y., Gallucci, G. O., Jung, R. E., Schneider, D., & Hämmerle, C. H. (2017). Guided bone regeneraBon and abutment connecBon augment the buccal soF Bssue contour: 3-year results of a prospecBve comparaBve clinical study. Clinical Oral Implants Research, 28(2), 219-225. h5ps://doi.org/10.1111/clr.12786
310. Be5er, H., Chaushu, L., Nissan, J., Xavier, S., Tallarico, M., & Chaushu, G. (2018). The Feasibility of Flapless Approach to Sinus AugmentaBon Using an Implant Device Designed According to Residual Alveolar Ridge Height. The InternaBonal journal of periodonBcs & restoraBve denBstry, 38(4), 601–606. h5ps://doi.org/10.11607/prd.2950
311. Bose, B. B., Natarajan, P. M., Kannan, A. L., Jebaraj, J. C., Jagannathan, R., & Balaji, T. M. (2022). EvaluaBon of Block AllograF Eﬃcacy in Lateral Alveolar Ridge AugmentaBon. The journal of contemporary dental pracBce, 23(8), 807-812. h5ps://doi.org/10.5005/jp-journals-10024-3377
312. Braun, D., Chappuis, V., Raabe, C., Suter, V. G. A., Fonseca, M., & Couso-Queiruga, E. (2025). PaBent-Reported Outcome Measures Following Implant Placement With Simultaneous Horizontal Bone AugmentaBon. Clinical Implant DenBstry and Related Research, 27(1), e70005. h5ps://doi.org/10.1111/cid.70005
313. Buser, D., Chappuis, V., Bornstein, M. M., Wi5neben, J. G., Frei, M., & Belser, U. C. (2013). Long-term stability of contour augmentaBon with early implant placement following single tooth extracBon in the estheBc zone: A prospecBve, cross-secBonal study in 41 paBents with a 5-to 9-year follow-up. Journal of Periodontology, 84(11), 1517-1527. h5ps://doi.org/10.1902/jop.2013.120635
314. Cansiz, E., Haq, J., Manisali, M., Cakarer, S., & Gultekin, B. A. (2020). Long-term evaluaBon of three-dimensional volumetric changes of augmented severely atrophic maxilla by anterior iliac crest bone graFing. Journal of stomatology, oral and maxillofacial surgery, 121(6), 665-671. h5ps://doi.org/10.1016/j.jormas.2019.11.004
315. Canullo, L., Del Fabbro, M., Colantonio, F., Iacono, R., Raﬀone, C., Pede5a, A., Khijmatgar, S., & Shapira, L. (2023). Sinus ﬂoor augmentaBon using crestal approach in conjuncBon with hydroxyapaBte/cross-linked collagen sponge: A pilot study. Clinical Implant DenBstry and Related Research, 25(5), 974-983. h5ps://doi.org/10.1111/cid.13236
316. Cardaropoli, D., Gaveglio, L., & Cardaropoli, G. (2013). VerBcal ridge augmentaBon with a collagen membrane, bovine bone mineral and ﬁbrin sealer: clinical and histologic ﬁndings. The InternaBonal journal of periodonBcs & restoraBve denBstry, 33(5), 583-589. h5ps://doi.org/10.11607/prd.1208
317. Ciocca, L., Lizio, G., Baldissara, P., Sambuco, A., Scoa, R., & Corinaldesi, G. (2018). ProstheBcally CAD-CAM-Guided Bone AugmentaBon of Atrophic Jaws Using Customized Titanium Mesh: Preliminary Results of an Open ProspecBve Study. The Journal of oral implantology, 44(2), 131-137. h5ps://doi.org/10.1563/aaid-joi-D-17-00125
318. Cucchi, A., Bianchi, A., Calamai, P., Rinaldi, L., Mangano, F., Vignudelli, E., & Corinaldesi, G. (2020). Clinical and volumetric outcomes aFer verBcal ridge augmentaBon using computer-aided-design/computer-aided manufacturing (CAD/CAM) customized Btanium meshes: a pilot study. BMC Oral Health, 20(1), 219. h5ps://doi.org/10.1186/s12903-020-01205-4
319. Daoud, S., Zoabi, A., Kasem, A., Totry, A., Oren, D., Redenski, I., Srouji, S., & Kablan, F. (2024). Computer-Assisted EvaluaBon Conﬁrms Spontaneous Healing of Donor Site One Year following Bone Block HarvesBng from Mandibular Retromolar Region—A Cohort Study. DiagnosBcs, 14(5). h5ps://doi.org/10.3390/diagnosBcs14050504
320. Deliberador, T. M., Vieira, J. S., Bonacin, R., Storrer, C. L., Santos, F. R., & Giovanini, A. F. (2015). ConnecBve Bssue graF combined with autogenous bone graF in the treatment of peri-implant soF and hard Bssue defect. Quintessence internaBonal (Berlin, Germany : 1985), 46(2), 139-144. h5ps://doi.org/10.3290/j.qi.a32824
321. Deluiz, D., Oliveira, L., Fletcher, P., Pires, F. R., Nunes, M. A., & Tinoco, E. M. (2016). Fresh-Frozen Bone AllograFs in Maxillary Alveolar AugmentaBon: Analysis of ComplicaBons, Adverse Outcomes, and Implant Survival. Journal of Periodontology, 87(11), 1261-1267. h5ps://[www.embase.com/search/results?subacBon=viewrecord&id=L621753867&from=export](http://www.embase.com/search/results?subacBon=viewrecord&id=L621753867&from=export)
322. Ersanli, S., Arısan, V., & Bedeloğlu, E. (2016). EvaluaBon of the autogenous bone block transfer for dental implant placement: Symphysal or ramus harvesBng? BMC Oral Health, 16, 4. h5ps://doi.org/10.1186/s12903-016-0161-8
323. Gan, L. M., Zhou, Q. R., Zhang, Y., Yu, Y. C., Yu, Z. Z., Sun, Y., Li, R. X., Wu, X. W., & Yang, F. (2024). Alveolar Bone

Morphologic Predictors for Guided Bone RegeneraBon Outcome in Anterior Maxilla. InternaBonal dental journal, 74(1), 102-109. h5ps://doi.org/10.1016/j.identj.2023.07.007

324. Hartmann, A., Hildebrandt, H., Younan, Z., Al-Nawas, B., & Kämmerer, P. W. (2022). Long-term results in three-dimensional, complex bone augmentaBon procedures with customized Btanium meshes. Clinical Oral Implants Research, 33(11), 1171-1181. h5ps://doi.org/10.1111/clr.14000
325. Isaksson, S., & Alberius, P. (1992). Maxillary alveolar ridge augmentaBon with onlay bone-graFs and immediate endosseous implants. Journal of Cranio-Maxillo-Facial Surgery, 20(1), 2-7. h5ps://[www.embase.com/search/results?subacBon=viewrecord&id=L22125586&from=export](http://www.embase.com/search/results?subacBon=viewrecord&id=L22125586&from=export)
326. Jiménez-Guerra, Á., Velasco-Ortega, E., Matos-Garrido, N., OrBz-García, I., Moreno-Muñoz, J., Núñez-Márquez, E., Rondón-Romero, J. L., Kewalramani, N., Salgado-Peralvo, Á. O., & Monsalve-Guil, L. (2025). Long-Term Clinical Outcomes of Transalveolar Maxillary Sinus Floor ElevaBon with Rotatory Instruments: An 8-Year Follow-Up ProspecBve Clinical Study. Journal of Clinical Medicine, 14(2). h5ps://doi.org/10.3390/jcm14020365
327. Koga, T., Nakatani, Y., Ohba, S., Hara, M., Sumita, Y., Nagai, K., & Asahina, I. (2021). Clinical safety assessment of autologous freeze-drying platelet-rich plasma for bone regeneraBon in maxillary sinus ﬂoor augmentaBon: A pilot study. Journal of Clinical Medicine, 10(8). h5ps://doi.org/10.3390/jcm10081678
328. Kolerman, R., Nissan, J., & Tal, H. (2014). Combined osteotome-induced ridge expansion and guided bone regeneraBon simultaneous with implant placement: a biometric study. Clinical Implant DenBstry and Related Research, 16(5), 691-704. h5ps://doi.org/10.1111/cid.12041
329. Kosanić, I., Andrić, M., Brković, B., Koković, V., & Jurišić, M. (2017). Unprotected autogenous bone block graFs in the anterior maxilla: ResorpBon rates and clinical outcomes. Vojnosanitetski Pregled, 74(4), 305-310. h5ps://doi.org/10.2298/VSP150429188K
330. Ku, J. K., Kim, J. Y., Kim, B. I., & Huh, J. K. (2021). EvaluaBon of wound dehiscence aFer verBcal bone graF by using quanBtaBve light-induced ﬂuorescence. Photodiagnosis and Photodynamic Therapy, 36. h5ps://doi.org/10.1016/j.pdpdt.2021.102470
331. Le, B., Hayashi, N., & Lee, U. (2024). InnovaBve Approach to Severe EstheBc Zone Trauma: IntegraBng Root Submergence With Guided Bone RegeneraBon. Journal of EstheBc and RestoraBve DenBstry. h5ps://doi.org/10.1111/jerd.13337
332. Leonard, J. F., Taxel, P., Kuo, C. L., Da Cunha Godoy, L., & Freilich, M. (2025). Dental implant and bone augmentaBon treatment in bone-compromised paBents: Oral health-related quality of life outcomes. The Journal of ProstheBc DenBstry, 133(1), 152-161. h5ps://doi.org/10.1016/j.prosdent.2023.01.011
333. Mayﬁeld, L., Nobréus, N., A5ström, R., & Linde, A. (1997). Guided bone regeneraBon in dental implant treatment using a bioabsorbable membrane. Clinical Oral Implants Research, 8(1), 10-17. h5ps://doi.org/10.1111/j.1600-0501.1997.tb00002.x
334. Merli, M., AquilanB, L., Merli, M., Marioa, G., & Rappelli, G. (2025). The TACOS Technique: A Stepwise Protocol for Alveolar Ridge AugmentaBon Using Customized Titanium Mesh. Medicina (Kaunas, Lithuania), 61(1). h5ps://doi.org/10.3390/medicina61010058
335. Mertens, C., Büsch, C., Ristow, O., Hoﬀmann, J., Wang, H. L., & Hoﬀmann, K. J. (2024). Iliac crest verBcal block graFs -placing outside or inside the bone contour: A cohort study. Clinical Implant DenBstry and Related Research, 26(6), 1069-1085. h5ps://doi.org/10.1111/cid.13370
336. Mu5anahally, K. S., Yadav, S., Freilich, M. A., & Tadinada, A. (2024). Does the Outcome of GraF Materials at Dental Implant Sites Diﬀer Between PaBents With Normal and Compromised Bone Health? The Journal of oral implantology, 50(3), 238-244. h5ps://doi.org/10.1563/aaid-joi-D-23-00078
337. Neira, P., Moreno, X., Caric, Y., Strauss, F. J., & Cavalla, F. (2024). Two-Step Periosteal Releasing Incision for Tension-Free Flap Closure AFer Implant Placement With Simultaneous Guided Bone RegeneraBon. The Journal of oral implantology, 50(4), 317-321. h5ps://doi.org/10.1563/aaid-joi-D-24-00032
338. Nissan, J., Ghelfan, O., Mardinger, O., Calderon, S., & Chaushu, G. (2011). Eﬃcacy of cancellous block allograF augmentaBon prior to implant placement in the posterior atrophic mandible. Clinical Implant DenBstry and Related Research, 13(4), 279-285. h5ps://doi.org/10.1111/j.1708-8208.2009.00219.x
339. Nord, T., Yüksel, O., Grimm, W. D., & Giesenhagen, B. (2019). One-Stage VerBcal Ridge AugmentaBon and Dental ImplantaBon With AllograF Bonerings: Results 1 Year AFer Surgery. The Journal of oral implantology, 45(6), 457-463. h5ps://doi.org/10.1563/aaid-joi-D-18-00257
340. Papi, P., Pranno, N., Di Murro, B., & Pompa, G. (2021). Early implant placement and peri-implant augmentaBon with a porcine-derived acellular dermal matrix and syntheBc bone in the aestheBc area: a 2-year follow-up prospecBve cohort study. InternaBonal Journal of Oral and Maxillofacial Surgery, 50(2), 258-266. h5ps://doi.org/10.1016/j.ijom.2020.07.002
341. Ramos-Murguialday, M., Caubet, J., Ramis, J. M., & Monjo, M. (2015). EvaluaBon of the Ideal Implant InserBon Time in Human Bone Biopsies AFer Sinus ElevaBon Using a CombinaBon of Autologous Bone and GraF SubsBtute. The InternaBonal journal of oral & maxillofacial implants, 30(4), 891-899. h5ps://doi.org/10.11607/jomi.3945
342. Silva, E. R., Ferraz, E. P., Neto, E. C., Chaushu, G., Chaushu, L., & Xavier, S. P. (2017). Volumetric Stability of Fresh Frozen Bone Blocks in Atrophic Posterior Mandible AugmentaBon. The Journal of oral implantology, 43(1), 25-

32. h5ps://doi.org/10.1563/aaid-joi-D-16-00095

343. Tang, Y., Zhai, S., Yu, H., & Qiu, L. (2023). Clinical feasibility evaluaBon of a digital workﬂow of prostheBcally oriented onlay bone graFing for horizontal alveolar augmentaBon: a prospecBve pilot study. BMC Oral Health, 23(1), 824. h5ps://doi.org/10.1186/s12903-023-03556-0
344. Vargas, S. M., Johnson, T. M., Pfaﬀ, A. S., Bumpers, A. P., Wagner, J. C., Retrum, J. K., Colamarino, A. N., BunBng,

M. E., Wilson, J. P., McDaniel, C. R., Herold, R. W., Stancoven, B. W., & Lincicum, A. R. (2023). Clinical protocol selecBon for alveolar ridge augmentaBon at sites exhibiBng slight, moderate, and severe horizontal ridge deﬁciencies. Clinical Advances in PeriodonBcs, 13(3), 174-196. h5ps://doi.org/10.1002/cap.10239

345. Wang, L., Wei, J. H., Yang, X., Yang, Z. H., Sun, M. Y., Cheng, X. B., Xu, L. Q., Lei, D. L., & Zhang, C. P. (2017).

PrevenBng Early-Stage GraF Bone ResorpBon by Simultaneous InnervaBon: Innervated Iliac Bone Flap for Mandibular ReconstrucBon. PlasBc and ReconstrucBve Surgery, 139(5), 1152e-1161e. h5ps://doi.org/10.1097/PRS.0000000000003263

346. Wu, V., Klein-Nulend, J., Bravenboer, N., Ten Bruggenkate, C. M., Helder, M. N., & Schulten, E. A. J. M. (2023). Long-Term Safety of Bone RegeneraBon Using Autologous Stromal Vascular FracBon and Calcium Phosphate Ceramics: A 10-Year ProspecBve Cohort Study. Stem Cells TranslaBonal Medicine, 12(9), 617-630. h5ps://doi.org/10.1093/stcltm/szad045
347. Yu, H., Chen, L., Zhu, Y., & Qiu, L. (2016). Bilamina corBcal tenBng graFing technique for three-dimensional reconstrucBon of severely atrophic alveolar ridges in anterior maxillae: A 6-year prospecBve study. Journal of Cranio-Maxillofacial Surgery, 44(7), 868-875. h5ps://doi.org/10.1016/j.jcms.2016.04.018
348. Yuce, M. O., Adali, E., Turk, G., Isik, G., & Gunbay, T. (2019). Three-dimensional bone graFing in dental implantology using autogenous bone ring transplant: Clinical outcomes of a one-stage technique. Nigerian journal of clinical pracBce, 22(7), 977-981. h5ps://doi.org/10.4103/njcp.njcp_652_18
349. Aaboe, M., Schou, S., HjørBng-Hansen, E., Helbo, M., & Vikjær, D. (2000). OsseointegraBon of subperiosteal implants using bovine bone subsBtute and various membranes. Clinical Oral Implants Research, 11(1), 51-58. h5ps://doi.org/10.1034/j.1600-0501.2000.011001051.x
350. Abbas, A. M., Bede, S. Y., & Alnumay, S. H. (2019). EvaluaBon of the eﬀecBveness of using platelet rich ﬁbrin (PRF) as a sole graFing material and membrane in augmentaBon of dehiscence and fenestraBon defects encountered during dental implant surgery. Journal of Baghdad College of DenBstry, 31(2), 44-51. h5ps://doi.org/10.26477/jbcd.v31i2.2623
351. Al-Khaldi, N., Sleeman, D., & Allen, F. (2011). Stability of dental implants in graFed bone in the anterior maxilla: Longitudinal study. BriBsh Journal of Oral and Maxillofacial Surgery, 49(4), 319-323. h5ps://doi.org/10.1016/j.bjoms.2010.05.009
352. Andersson, B., Ödman, P., Widmark, G., & Waas, A. (1993). Anterior tooth replacement with implants in paBents with a narrow alveolar ridge form. A clinical study using guided Bssue regeneraBon. Clinical Oral Implants Research, 4(2), 90-98. h5ps://doi.org/10.1034/j.1600-0501.1993.040205.x
353. Angermair, J., Bosshardt, D. D., Nelson, K., Flügge, T. V., Stricker, A., & Fretwurst, T. (2020). Horizontal bone graFing using equine-derived cancellous bone blocks is associated with severe complicaBons: A prospecBve clinical and histological pilot study. Clinical Oral Implants Research, 31(11), 1149-1158. h5ps://doi.org/10.1111/clr.13661
354. Arora, R., Verma, U. P., Dixit, J., & Lal, N. (2017). SynergisBc eﬀects of growth factor, bone graF, and resorbable barrier membrane in management of dehiscence and fenestraBon of dental implants. World Journal of DenBstry, 8(3), 177-182. h5ps://doi.org/10.5005/jp-journals-10015-1434
355. Artzi, Z., Nemcovsky, C. E., & Dayan, D. (2002). Bovine-HA spongiosa blocks and immediate implant placement in sinus augmentaBon procedures: Histopathological and histomorphometric observaBons on diﬀerent histological stainings in 10 consecuBve paBents. Clinical Oral Implants Research, 13(4), 420-427. h5ps://doi.org/10.1034/j.1600-0501.2002.130411.x
356. Bahat, O., & Fontanessi, R. V. (2001). Implant Placement in Three-Dimensional GraFs in the Anterior Jaw. InternaBonal Journal of PeriodonBcs and RestoraBve DenBstry, 21(4), 357-365. h5ps://[www.scopus.com/inward/record.uri?eid=2-s2.0-](http://www.scopus.com/inward/record.uri?eid=2-s2.0-)0035431157&partnerID=40&md5=ebc2215e40b00dcd8393e66a23f37d70
357. Bahat, O., & Fontanessi, R. V. (2001). Eﬃcacy of Implant Placement aFer Bone GraFing for Three-Dimensional ReconstrucBon of the Posterior Jaw. InternaBonal Journal of PeriodonBcs and RestoraBve DenBstry, 21(3), 221-

231. h5ps://[www.scopus.com/inward/record.uri?eid=2-s2.0-](http://www.scopus.com/inward/record.uri?eid=2-s2.0-)0035375937&partnerID=40&md5=a0c3306722ddf900f2d8f8facdc8027e

358. Bahat, O., Sullivan, R. M., & Smidt, A. (2012). Placement of Brånemark Mk IV implants in compromised and graFed bone: Radiographic outcome of 61 sites in 27 paBents with 3- to 7-year follow-ups. Quintessence InternaBonal, 43(4), 293-303. h5ps://[www.scopus.com/inward/record.uri?eid=2-s2.0-](http://www.scopus.com/inward/record.uri?eid=2-s2.0-)84891475859&partnerID=40&md5=fa109da1fa23b6ec8e9c1ad98cafd05c
359. Becker, W., Hujoel, P., & Becker, B. E. (2002). Eﬀect of barrier membranes and autologous bone graFs on ridge width preservaBon around implants. Clinical Implant DenBstry and Related Research, 4(3), 143-149. h5ps://doi.org/10.1111/j.1708-8208.2002.tb00165.x
360. Beitlitum, I., Artzi, Z., & Nemcovsky, C. E. (2010). Clinical evaluaBon of parBculate allogeneic with and without autogenous bone graFs and resorbable collagen membranes for bone augmentaBon of atrophic alveolar ridges. Clinical Oral Implants Research, 21(11), 1242-1250. h5ps://doi.org/10.1111/j.1600-0501.2010.01936.x
361. Beitlitum, I., Sebaoun, A., Nemcovsky, C. E., & Slutzkey, S. (2018). Lateral bone augmentaBon in narrow posterior mandibles, descripBon of a novel approach, and analysis of results. Clinical Implant DenBstry and Related Research, 20(2), 96-101. h5ps://doi.org/10.1111/cid.12580
362. Beltrán, V., Engelke, W., Fuentes, R., Decco, O., Prieto, R., Wilckens, M., & Borie, E. (2014). Bone augmentaBon with occlusive barriers and corBcal parBculate allograF in transverse maxillary defects: A pilot study. InternaBonal Journal of Morphology, 32(1), 364-368. h5ps://doi.org/10.4067/S0717-95022014000100057
363. Benic, G. I., Bernasconi, M., Jung, R. E., & Hämmerle, C. H. F. (2017). Clinical and radiographic intra-subject comparison of implants placed with or without guided bone regeneraBon: 15-year results. Journal of Clinical Periodontology, 44(3), 315-325. h5ps://doi.org/10.1111/jcpe.12665
364. Benic, G. I., Joo, M. J., Yoon, S. R., Cha, J. K., & Jung, U. W. (2017). Primary ridge augmentaBon with collagenated xenogenic block bone subsBtute in combinaBon with collagen membrane and rhBMP-2: a pilot histological invesBgaBon. Clinical Oral Implants Research, 28(12), 1543-1552. h5ps://doi.org/10.1111/clr.13024
365. Benić, G. I., Jung, R. E., Siegenthaler, D. W., & Hämmerle, C. H. F. (2009). Clinical and radiographic comparison of implants in regenerated or naBve bone: 5-year results. Clinical Oral Implants Research, 20(5), 507-513. h5ps://doi.org/10.1111/j.1600-0501.2008.01583.x
366. Bolot, M. Y. A., Ghoraba, S. M., & Badr, A. M. (2024). Horizontal bone augmentaBon with simultaneous implant placement using ridge spliang with sBcky bone. Tanta Dental Journal, 21(4), 479-489. h5ps://doi.org/10.4103/tdj.tdj_21_24
367. Bormann, K. H., Suarez-Cunqueiro, M. M., von See, C., Kokemüller, H., Schumann, P., & Gellrich, N. C. (2010). Sandwich osteotomy for verBcal and transversal augmentaBon of the posterior mandible. InternaBonal Journal of Oral and Maxillofacial Surgery, 39(6), 554-560. h5ps://doi.org/10.1016/j.ijom.2010.03.002
368. Cooper, L. F., Reside, G., Raes, F., Garriga, J. S., Tarrida, L. G., Wilõang, J., Kern, M., & De Bruyn, H. (2014). Immediate provisionalizaBon of dental implants in graFed alveolar ridges in the estheBc zone: A 5-year evaluaBon. InternaBonal Journal of PeriodonBcs and RestoraBve DenBstry, 34(4), 476-486. h5ps://doi.org/10.11607/prd.2022
369. Dawar, A., Yadav, V. S., Nanda, A., & Gupta, V. (2025). Short-term paBent-reported outcomes correlate with clinical parameters in guided bone regeneraBon: Dental implants. Evidence-Based DenBstry, 26(1), 32-33. h5ps://doi.org/10.1038/s41432-025-01115-w
370. Dias, R. R., Sehn, F. P., de Santana Santos, T., Silva, E. R., Chaushu, G., & Xavier, S. P. (2016). CorBcocancellous fresh-frozen allograF bone blocks for augmenBng atrophied posterior mandibles in humans. Clinical Oral Implants Research, 27(1), 39-46. h5ps://doi.org/10.1111/clr.12509
371. El-Khoury, N., Menassa, G., Chakar, C., Kassir, A. R., & Naaman, N. (2023). CLINICAL AND RADIOGRAPHIC EVALUATION OF BONE REMODELING AROUND IMPLANTS PLACED IN HORIZONTALLY AUGMENTED BONE: A

PILOT STUDY. InternaBonal Arab Journal of DenBstry, 14(2), 112-119. h5ps://[www.scopus.com/inward/record.uri?eid=2-s2.0-](http://www.scopus.com/inward/record.uri?eid=2-s2.0-)85177555802&partnerID=40&md5=a2b71808e16496e3a93345ef2f77aö4

372. Erdogan, O., Uçar, Y., Tatli, U., Sert, M., Benlidayi, M. E., & Evlice, B. (2015). A clinical prospecBve study on alveolar bone augmentaBon and dental implant success in paBents with type 2 diabetes. Clinical Oral Implants Research, 26(11), 1267-1275. h5ps://doi.org/10.1111/clr.12450
373. Esposito, M., Pellegrino, G., PisBlli, R., & Felice, P. (2011). RehabilitaBon of posterior atrophic edentulous jaws: Prostheses supported by 5 mm short implants or by longer implants in augmented bone? One-year results from a pilot randomised clinical trial. European Journal of Oral Implantology, 4(1), 21-30. h5ps://[www.scopus.com/inward/record.uri?eid=2-s2.0-](http://www.scopus.com/inward/record.uri?eid=2-s2.0-)79958261339&partnerID=40&md5=4def874d4036a33279b004b2c25ö492
374. Falisi, G., Galli, M., Vi5orini-Velasquez, P., Gallegos-Rivera, J. C., Minasi, R., De Biase, A., & Di Paolo, C. (2013). Use of 3D carBlage scaﬀolds for the stabilizaBon of implants and bone regeneraBon with the ﬁt-lock technique. Acta odontológica laBnoamericana : AOL, 26(3), 167-172. h5ps://[www.scopus.com/inward/record.uri?eid=2-](http://www.scopus.com/inward/record.uri?eid=2-)s2.0-84910014706&partnerID=40&md5=d2b35a17ad0dfdcaf2a96e51b8cd8d26
375. Fanai, J. V., Dhuvad, J., Anchlia, S., Bha5, U., Trivedi, B., & Jogani, H. (2024). QuanBtaBve EvaluaBon of ZygomaBc Bu5ress, Ramal, and Symphysis Onlay GraFs in Ridge AugmentaBon Prior to Implant Placement. Journal of Maxillofacial and Oral Surgery. h5ps://doi.org/10.1007/s12663-024-02192-7
376. Fekry, Y. E. S., & Mahmoud, N. R. (2023). VerBcal ridge augmentaBon of atrophic posterior mandible with corBcocancellous onlay symphysis graF versus sandwich technique: clinical and radiographic analysis. Odontology, 111(4), 993-1002. h5ps://doi.org/10.1007/s10266-023-00794-0
377. Felice, P., Marchea, C., Pia5elli, A., Pellegrino, G., Checchi, V., Worthington, H., & Esposito, M. (2008). VerBcal ridge augmentaBon of the atrophic posterior mandible with interposiBonal block graFs: Bone from the iliac crest versus bovine anorganic bone. Results up to delivery of the ﬁnal prostheses from a split-mouth, randomised controlled clinical trial. European Journal of Oral Implantology, 1(3), 183-198.

h5ps://[www.scopus.com/inward/record.uri?eid=2-s2.0-](http://www.scopus.com/inward/record.uri?eid=2-s2.0-)70449410304&partnerID=40&md5=5a6920a2569a4f2211620a1a42adb708

378. Felice, P., PisBlli, R., Lizio, G., Pellegrino, G., Nisii, A., & Marchea, C. (2009). Inlay versus onlay iliac bone graFing in atrophic posterior mandible: A prospecBve controlled clinical trial for the comparison of two techniques. Clinical Implant DenBstry and Related Research, 11(SUPPL. 1), e69-e82. h5ps://doi.org/10.1111/j.1708-8208.2009.00212.x
379. Fenlon, M. R., Lyons, A., Farrell, S., Bavisha, K., Banerjee, A., & Palmer, R. M. (2012). Factors AﬀecBng Survival and Usefulness of Implants Placed in Vascularized Free Composite GraFs Used in Post-Head and Neck Cancer ReconstrucBon. Clinical Implant DenBstry and Related Research, 14(2), 266-272. h5ps://doi.org/10.1111/j.1708-8208.2009.00250.x
380. Friedmann, A., Strietzel, F. P., Maretzki, B., Pitaru, S., & Bernimoulin, J. P. (2002). Histological assessment of augmented jaw bone uBlizing a new collagen barrier membrane compared to a standard barrier membrane to protect a granular bone subsBtute material: A randomized clinical trial. Clinical Oral Implants Research, 13(6), 587-594. h5ps://doi.org/10.1034/j.1600-0501.2002.130603.x
381. Hakobyan, G. (2005). Bone griFing procedures for osseous defects associated with dental implants. The Journal of oral implantology, 31(3), 145-152. h5ps://doi.org/10.1563/1548-1336(2005)31[145:BGPFOD]2.0.CO;2
382. Hämmerle, C. H. F., & Lang, N. P. (2001). Single stage surgery combining transmucosal implant placement with guided bone regeneraBon and bioresorbable materials. Clinical Oral Implants Research, 12(1), 9-18. h5ps://doi.org/10.1034/j.1600-0501.2001.012001009.x
383. Huang, L. R., Zhong, Y. J., Zhang, X. Q., Feng, Z. R., Lai, Y. C., Wu, H. K., & Mo, A. C. (2025). ComparaBve evaluaBon of allograF parBculate bone and corBcal bone blocks combined with xenograF bone for labial bone defects in the aestheBc zone: a prospecBve cohort study. BMC Oral Health, 25(1). h5ps://doi.org/10.1186/s12903-025-05443-2
384. Jaquiéry, C., Rohner, D., Kunz, C., Bucher, P., Peters, F., Schenk, R. K., & Hammer, B. (2004). ReconstrucBon of maxillary and mandibular defects using prefabricated microvascular ﬁbular graFs and osseointegrated dental implants - A prospecBve study. Clinical Oral Implants Research, 15(5), 598-606. h5ps://doi.org/10.1111/j.1600-0501.2004.01065.x
385. Jensen, S. S., Bosshardt, D. D., Gruber, R., & Buser, D. (2014). Long-term stability of contour augmentaBon in the estheBc zone: Histologic and histomorphometric evaluaBon of 12 human biopsies 14 to 80 months aFer augmentaBon. Journal of Periodontology, 85(11), 1549-1556. h5ps://doi.org/10.1902/jop.2014.140182
386. Jepsen, K., Jepsen, S., Laine, M. L., Anssari Moin, D., Pilloni, A., Zeza, B., Sanz, M., OrBz-Vigon, A., Roos-Jansåker, A. M., & Renvert, S. (2016). ReconstrucBon of Peri-implant Osseous Defects: A MulBcenter Randomized Trial. Journal of Dental Research, 95(1), 58-66. h5ps://doi.org/10.1177/0022034515610056
387. Kawasaki, T., Ohba, S., Nakatani, Y., & Asahina, I. (2018). Clinical study of guided bone regeneraBon with resorbable polylacBde-co-glycolide acid membrane. Odontology, 106(3), 334-339. h5ps://doi.org/10.1007/s10266-018-0349-2
388. Kim, E. S. (2015). Autogenous fresh demineralized tooth graF prepared at chairside for dental implant. Maxillofacial PlasBc and ReconstrucBve Surgery, 37(1). h5ps://doi.org/10.1186/s40902-015-0009-1
389. Kim, H. S., Park, J. C., Yun, P. Y., & Kim, Y. K. (2017). EvaluaBon of bone healing using rhBMP-2 soaked hydroxyapaBte in ridge augmentaBon: a prospecBve observaBonal study. Maxillofacial PlasBc and ReconstrucBve Surgery, 39(1). h5ps://doi.org/10.1186/s40902-017-0138-9
390. Koﬁna, V., Monfaredzadeh, M., Rawal, S. Y., DenBno, A. R., Singh, M., & Tatakis, D. N. (2023). PaBent-reported outcomes following guided bone regeneraBon: CorrelaBon with clinical parameters. Journal of DenBstry, 136. h5ps://doi.org/10.1016/j.jdent.2023.104605
391. Lee, E. A., Prasad, H., & Lynch, S. (2024). SequenBal Human Histology Results of the Subperiosteal Minimally Invasive AestheBc Ridge AugmentaBon Technique (SMART): A Chronologic Wound Healing Proof-of-Principle Study. InternaBonal Journal of PeriodonBcs and RestoraBve DenBstry, 44(1), 38-49. h5ps://doi.org/10.11607/prd.6559
392. Leiva-Gea, L., Sánchez-Palomino, P., Lendínez-Jurado, A., Corte-Torres, M. D., Leiva-Gea, I., & Leiva-Gea, A. (2025). EﬀecBveness of Titanium Occlusive Barriers in Guided Bone RegeneraBon: A ProspecBve Analysis of VerBcal and Horizontal Bone AugmentaBon. BiomimeBcs, 10(3). h5ps://doi.org/10.3390/biomimeBcs10030165
393. Lenssen, O., Barbier, L., & De Clercq, C. (2011). Immediate funcBonal loading of provisional implants in the reconstructed atrophic maxilla: Preliminary results of a prospecBve study aFer 6 months of loading with a

provisional bridge. InternaBonal Journal of Oral and Maxillofacial Surgery, 40(9), 907-915. h5ps://doi.org/10.1016/j.ijom.2011.05.001

394. Liang, C., Wang, Y., Li, J., & Geng, W. (2025). EvaluaBon of the eﬀects of the small intesBne submucosa biological membrane on guided bone regeneraBon and early loading of dental implants in the maxillary anterior region: A randomized controlled trial. Journal of ProstheBc DenBstry. h5ps://doi.org/10.1016/j.prosdent.2025.02.030
395. Lizio, G., Pellegrino, G., Corinaldesi, G., Ferri, A., Marchea, C., & Felice, P. (2022). Guided bone regeneraBon using Btanium mesh to augment 3-dimensional alveolar defects prior to implant placement. A pilot study. Clinical Oral Implants Research, 33(6), 607-621. h5ps://doi.org/10.1111/clr.13922
396. Lorenz, J., GhanaaB, S., Aleksic, Z., Milinkovic, I., Lazic, Z., Magić, M., Wessing, B., Grotenclos, R. S., Merli, M., Marioa, G., Bressan, E., De Stavola, L., & Sader, R. (2025). Horizontal Guided Bone RegeneraBon of the Posterior Mandible to Allow Implant Placement: 1-Year ProspecBve Study Results. Clinical Oral Implants Research, 36(1), 100-116. h5ps://doi.org/10.1111/clr.14363
397. Lorenzoni, M., Pertl, C., Keil, C., & Wegscheider, W. A. (1998). Treatment of Peri-implant Defects with Guided Bone RegeneraBon: A ComparaBve Clinical Study with Various Membranes and Bone GraFs. InternaBonal Journal of Oral and Maxillofacial Implants, 13(5), 639-646. h5ps://[www.scopus.com/inward/record.uri?eid=2-](http://www.scopus.com/inward/record.uri?eid=2-)s2.0-0032160624&partnerID=40&md5=634c68c854f51879841102aa99e89963
398. Maiorana, C., Manfredini, M., Bere5a, M., Signorino, F., Bovio, A., & Poli, P. P. (2020). Clinical and radiographic evaluaBon of simultaneous alveolar ridge augmentaBon by means of preformed Btanium meshes at dehiscence-type peri-implant defects: A prospecBve pilot study. Materials, 13(10). h5ps://doi.org/10.3390/ma13102389
399. Maiorana, C., Santoro, F., RabagliaB, M., & Salina, S. (2001). EvaluaBon of the Use of Iliac Cancellous Bone and Anorganic Bovine Bone in the ReconstrucBon of the Atrophic Maxilla with Titanium Mesh: A Clinical and Histologic InvesBgaBon. InternaBonal Journal of Oral and Maxillofacial Implants, 16(3), 427-432. h5ps://[www.scopus.com/inward/record.uri?eid=2-](http://www.scopus.com/inward/record.uri?eid=2-)
400. s2.0-0035349227&partnerID=40&md5=21941567cdd5db8182bd0577f1a4e796
401. Malchiodi, L., Quaranta, A., D'Addona, A., Scarano, A., & Quaranta, M. (2006). Jaw ReconstrucBon With GraFed Autologous Bone: Early InserBon of Osseointegrated Implants and Early ProstheBc Loading. Journal of Oral and Maxillofacial Surgery, 64(8), 1190-1198. h5ps://doi.org/10.1016/j.joms.2006.04.015
402. Mannai, C. (2006). Early Implant Loading in Severely Resorbed Maxilla Using XenograF, AutograF, and Platelet-Rich Plasma in 97 PaBents. Journal of Oral and Maxillofacial Surgery, 64(9), 1420-1426. h5ps://doi.org/10.1016/j.joms.2006.05.028
403. Marques, E., Cosmo, L. A. M., Teixeira, M. L., de Macedo, L. G. S., Aloise, A. C., & Pelegrine, A. A. (2023). Horizontal Bone AugmentaBon With Autogenous and Collagenated Xenogeneic Bone Blocks: A Split-Mouth ProspecBve Clinical, Tomographic, and Histological Pilot Study. Journal of Oral Implantology, 49(3), 253-261. h5ps://doi.org/10.1563/aaid-joi-D-22-00115
404. Meloni, S. M., Jovanovic, S. A., Pisano, M., De Riu, G., Baldoni, E., & Tallarico, M. (2018). One-stage horizontal guided bone regeneraBon with autologous bone, anorganic bovine bone and collagen membranes: Follow-up of a prospecBve study 30 months aFer loading. European Journal of Oral Implantology, 11(1), 89-95. h5ps://[www.scopus.com/inward/record.uri?eid=2-s2.0-](http://www.scopus.com/inward/record.uri?eid=2-s2.0-)85044113838&partnerID=40&md5=0274a5e070d3b111b44dfd9ba34dcd89
405. Mertens, C., Thiele, O., Engel, M., Seeberger, R., Hoﬀmann, J., & Freier, K. (2015). The use of self-inﬂaBng soF Bssue expanders prior to bone augmentaBon of atrophied alveolar ridges. Clinical Implant DenBstry and Related Research, 17(1), 44-51. h5ps://doi.org/10.1111/cid.12093
406. Minea, E., Celko, M., Contessi, M., Carini, F., Gambardella, U., Giacomea, E., SanBllana, J., Campoy, T. B., Schmitz, J. H., Libertucci, M., Ho, H., Haan, S., & Mastrangelo, F. (2021). Implants survival rate in regenerated sites with innovaBve graF biomaterials: 1 year follow-up. Materials, 14(18). h5ps://doi.org/10.3390/ma14185292
407. Mohammadi, H., Shirmohammadi, A., Babaloo, A., Roshangar, L., Torab, Z., & Mojtahedinia, M. (2022). EvaluaBon of the eﬀect of autologous condiBoned serum on the radiographic characterisBcs of hard Bssue aFer horizontal bone augmentaBon in implant denBstry. Journal of Advanced Periodontology and Implant DenBstry, 14(2), 62-68. h5ps://doi.org/10.34172/japid.2022.015
408. Monje, A., Monje, F., Suárez, F., González-García, R., Villanueva-Alcojol, L., García-Nogales, A., Galindo-Moreno, P., & Wang, H. L. (2013). Comparison of implant primary stability between maxillary edentulous ridges receiving intramembranous origin block graFs. Medicina Oral, Patologia Oral y Cirugia Bucal, 18(3), e449-e454. h5ps://doi.org/10.4317/medoral.18732
409. Naldini, P., Torassa, D., Calvo-Guirado, J. L., & Bodereau, E. F. (2022). EvaluaBon of Contour AugmentaBon in Immediate Single-Tooth Implants With and Without Flaps in the Anterior Maxilla: A 1-Year ProspecBve Study. InternaBonal Journal of PeriodonBcs and RestoraBve DenBstry, 42(3), 331-339. h5ps://doi.org/10.11607/prd.4619
410. Nissan, J., Mardinger, O., Strauss, M., Peleg, M., Sacco, R., & Chaushu, G. (2011). Implant-supported restoraBon of congenitally missing teeth using cancellous bone block-allograFs. Oral Surgery, Oral Medicine, Oral Pathology, Oral Radiology and Endodontology, 111(3), 286-291. h5ps://doi.org/10.1016/j.tripleo.2010.04.042
411. Nyström, E., Ahlqvist, J., Kahnberg, K. E., & Rosenquist, J. B. (1996). Autogenous onlay bone graFs ﬁxed with screw implants for the treatment of severely resorbed maxillae: Radiographic evaluaBon of preoperaBve bone dimensions, postoperaBve bone loss, and changes in soF-Bssue proﬁle. InternaBonal Journal of Oral and Maxillofacial Surgery, 25(5), 351-359. h5ps://doi.org/10.1016/S0901-5027(06)80029-9
412. Nyström, E., Ahlqvist, J., Legrell, P. E., & Kahnberg, K. E. (2002). Bone graF remodelling and implant success rate in the treatment of the severely resorbed maxilla: A 5-year longitudinal study. InternaBonal Journal of Oral and Maxillofacial Surgery, 31(2), 158-164. h5ps://doi.org/10.1054/ijom.2001.0197
413. Orsini, G., Stacchi, C., VisinBni, E., Di Iorio, D., PuBgnano, A., Breschi, L., & Di Lenarda, R. (2011). Clinical and histologic evaluaBon of fresh frozen human bone graFs for horizontal reconstrucBon of maxillary alveolar ridges. InternaBonal Journal of PeriodonBcs and RestoraBve DenBstry, 31(5), 535-544. h5ps://[www.scopus.com/inward/record.uri?eid=2-s2.0-](http://www.scopus.com/inward/record.uri?eid=2-s2.0-)84857393018&partnerID=40&md5=b7db6f2cbc8ee348bdc36ﬀ3e4769a6c
414. Palmer, R. M., Floyd, P. D., Palmer, P. J., Smith, B. J., Johansson, C. B., & Albrektsson, T. (1994). Healing of implant dehiscence defects with without expanded polytetraﬂuoroethylene membranes: a controlled clinical and histological study and histological study. Clinical Oral Implants Research, 5(2), 98-104. h5ps://doi.org/10.1034/j.1600-0501.1994.050206.x
415. Parma-BenfenaB, S., TinB, C., Albrektsson, T., & Johansson, C. (1999). Histologic EvaluaBon of Guided VerBcal Ridge AugmentaBon Around Implants in Humans. InternaBonal Journal of PeriodonBcs and RestoraBve DenBstry, 19(5), 425-437. h5ps://[www.scopus.com/inward/record.uri?eid=2-s2.0-](http://www.scopus.com/inward/record.uri?eid=2-s2.0-)0033201588&partnerID=40&md5=041066cae0c4763524b8a053d983d704
416. Pieri, F., Aldini, N. N., Marchea, C., & Corinaldesi, G. (2013). EstheBc outcome and Bssue stability of maxillary anterior single-tooth implants following reconstrucBon with mandibular block graFs: A 5-year prospecBve study. InternaBonal Journal of Oral and Maxillofacial Implants, 28(1), 270-280. h5ps://doi.org/10.11607/jomi.2560
417. Rabha, P. K., Debnath, S. C., Chishi, A. N., Nath, P., & Dhanushya, A. (2025). Guided Bone RegeneraBon (GBR) Using a Collagen Membrane Barrier Technique Along With Full Thickness Buccal Mucoperiosteal Flap For the

PreservaBon of the Alveolar Ridge Following Tooth ExtracBon. Journal of Maxillofacial and Oral Surgery. h5ps://doi.org/10.1007/s12663-024-02419-7

418. Roccuzzo, M., Savoini, M., Dalmasso, P., & Ramieri, G. (2017). Long-term outcomes of implants placed aFer verBcal alveolar ridge augmentaBon in parBally edentulous paBents: a 10-year prospecBve clinical study. Clinical Oral Implants Research, 28(10), 1204-1210. h5ps://doi.org/10.1111/clr.12941
419. Smolka, W., Bosshardt, D. D., Mericske-Stern, R., & Iizuka, T. (2006). ReconstrucBon of the severely atrophic mandible using calvarial split bone graFs for implant-supported oral rehabilitaBon. Oral Surgery, Oral Medicine, Oral Pathology, Oral Radiology, and EndodonBcs, 101(1), 35-42. h5ps://doi.org/10.1016/j.tripleo.2005.03.022
420. Soni, R., Priya, A., Agrawal, R., Bhatnagar, A., & Kumar, L. (2020). EvaluaBon of eﬃcacy of platelet-rich ﬁbrin membrane and bone graF in coverage of immediate dental implant in estheBc zone: An in vivo study. NaBonal Journal of Maxillofacial Surgery, 11(1), 67-75. h5ps://doi.org/10.4103/njms.NJMS_26_19
421. Spin-Neto, R., Stavropoulos, A., De Freitas, R. M., Pereira, L. A. V. D., Carlos, I. Z., & Marcantonio, E. (2013). Immunological aspects of fresh-frozen allogeneic bone graFing for lateral ridge augmentaBon. Clinical Oral Implants Research, 24(9), 963-968. h5ps://doi.org/10.1111/j.1600-0501.2012.02510.x
422. Steigmann, M. (2006). Pericardium membrane and xenograF parBculate graFing materials for horizontal alveolar ridge defects. Implant DenBstry, 15(2), 186-191. h5ps://doi.org/10.1097/01.id.0000202481.96644.f4
423. Tabrizi, R., Aboul-Hosn Centenero, S., HazraB, P., & Azadi, A. (2024). Comparison of the success rate and marginal bone loss of implants placed simultaneously with either bone expansion or ridge spliang in maxillary sites: a prospecBve non-randomized study. InternaBonal Journal of Oral and Maxillofacial Surgery, 53(9), 787-794. h5ps://doi.org/10.1016/j.ijom.2024.04.008
424. Verhamme, L. M., Meijer, G. J., Bergé, S. J., Soehardi, R. A., Xi, T., de Haan, A. F. J., Schutyser, F., & Maal, T. J. J. (2015). An Accuracy Study of Computer-Planned Implant Placement in the Augmented Maxilla Using Mucosa-Supported Surgical Templates. Clinical Implant DenBstry and Related Research, 17(6), 1154-1163. h5ps://doi.org/10.1111/cid.12230
425. Wang, W., Jiang, Y., Wang, D., Mei, D., Xu, H., & Zhao, B. (2022). Clinical eﬃcacy of autogenous denBn graFs with guided bone regeneraBon for horizontal ridge augmentaBon: a prospecBve observaBonal study. InternaBonal Journal of Oral and Maxillofacial Surgery, 51(6), 837-843. h5ps://doi.org/10.1016/j.ijom.2021.06.012
426. Widmark, C., Andersson, B., Andrup, B., Carlsson, G. E., Ivanoﬀ, C. J., & Lindvall, A. M. (1998). RehabilitaBon of PaBents with Severely Resorbed Maxillae by Means of Implants with or Without Bone GraFs. A 1-Year Follow-up Study. InternaBonal Journal of Oral and Maxillofacial Implants, 13(4), 474-482. h5ps://[www.scopus.com/inward/record.uri?eid=2-s2.0-](http://www.scopus.com/inward/record.uri?eid=2-s2.0-)0032112632&partnerID=40&md5=4d6c74aa9cd5bbb8bebe50ea7f388115
427. Wi5enberg, J. M., & Small, I. A. (1995). Five-year follow-up of mandibular reconstrucBon with hydroxylapaBte and the mandibular staple bone plate. Journal of Oral and Maxillofacial Surgery, 53(1), 19-22. h5ps://doi.org/10.1016/0278-2391(95)90491-3
428. Wu, Y. Q., Huang, W., Zhang, Z. Y., Zhang, Z. Y., Zhang, C. P., & Sun, J. (2008). Clinical outcome of dental implants placed in ﬁbula-free ﬂaps for orofacial reconstrucBon. Chinese Medical Journal, 121(19), 1861-1865. h5ps://doi.org/10.1097/00029330-200810010-00002

429 Yu, H., & Qiu, L. (2018). Eﬃcacy of the split-thickness labial ﬂap method for soF Bssue management in anterior ridge horizontal augmentaBon procedures: A clinical prospecBve study in the anterior maxilla. Journal of Cranio-Maxillofacial Surgery, 46(2), 323-328. h5ps://doi.org/10.1016/j.jcms.2017.11.01
